# Enhancing mutation impact prediction in protein-protein interactions through interpretable graph-based multi-level feature interactions

**DOI:** 10.1093/bioinformatics/btag150

**Published:** 2026-03-27

**Authors:** Shiwei Wu, Nan Xu, Xiaohui Xin, Min Zhang, Haoliang Liu, Hongjia Zhu, Zhenyu Wei, Chengkui Zhao, Lei Yu, Weixing Feng

**Affiliations:** College of Intelligent Systems Science and Engineering, Harbin Engineering University, Harbin, China; Institute of Biomedical Engineering and Technology, Shanghai Engineering Research Center of Molecular Therapeutics and New Drug Development, School of Chemistry and Molecular Engineering, East China Normal University, Shanghai, China; Shanghai Unicar-Therapy Bio-medicine Technology Co., Ltd, Shanghai, China; College of Intelligent Systems Science and Engineering, Harbin Engineering University, Harbin, China; College of Intelligent Systems Science and Engineering, Harbin Engineering University, Harbin, China; College of Intelligent Systems Science and Engineering, Harbin Engineering University, Harbin, China; Institute of Biomedical Engineering and Technology, Shanghai Engineering Research Center of Molecular Therapeutics and New Drug Development, School of Chemistry and Molecular Engineering, East China Normal University, Shanghai, China; Shanghai Unicar-Therapy Bio-medicine Technology Co., Ltd, Shanghai, China; College of Intelligent Systems Science and Engineering, Harbin Engineering University, Harbin, China; College of Intelligent Systems Science and Engineering, Harbin Engineering University, Harbin, China; Shanghai Unicar-Therapy Bio-medicine Technology Co., Ltd, Shanghai, China; Institute of Biomedical Engineering and Technology, Shanghai Engineering Research Center of Molecular Therapeutics and New Drug Development, School of Chemistry and Molecular Engineering, East China Normal University, Shanghai, China; Shanghai Unicar-Therapy Bio-medicine Technology Co., Ltd, Shanghai, China; College of Intelligent Systems Science and Engineering, Harbin Engineering University, Harbin, China

## Abstract

**Motivation:**

Protein–protein interactions (PPIs) are central to cellular functions, and predicting mutation-induced changes in binding affinity (ΔΔG) remains challenging. Although existing computational methods integrate sequence- and structure-derived features and thus implicitly capture certain sequence–structure relationships, they typically fuse these modalities through simple concatenation, without explicitly modeling their multidimensional and multiscale interdependencies.

**Results:**

Here, we introduce IGMI, an interpretable graph-based model that explicitly encodes multi-level feature interactions across 1D sequences, 2D contact maps, 3D structures, and residue- and atom-level representations. By recalibrating cross-dimensional and cross-scale dependencies, IGMI enables more accurate estimation of both local and long-range mutation effects. Across multiple benchmark datasets, IGMI consistently outperforms state-of-the-art methods in accuracy, robustness, and interpretability. Macro- and micro-level analyses further reveal biologically plausible patterns, distinguishing direct interface perturbations from indirect structural reorganizations. Complementary analyses under different data splitting strategies indicate that the model learns generalizable affinity-related interaction patterns, rather than relying on split-specific information. IGMI provides a reliable and interpretable framework for modeling mutation-induced affinity changes, supporting applications in protein engineering and therapeutic design.

**Availability and implementation:**

IGMI is implemented in PyTorch and released under an open-source license. The full codebase, training scripts, and evaluation utilities are available at https://github.com/ShiweiWu-545/IGMI.git. An archival snapshot containing all source code, pre-trained weights, processed datasets, and reproducibility scripts is available on Zenodo (https://doi.org/10.5281/zenodo.17563574).

**Contact:**

fengweixing@hrbeu.edu.cn; yulei@nbic.ecnu.edu.cn; zhaochengkui@hrbeu.edu.cn

**Supplementary information:**

Supplementary data are available at Bioinformatics online.

## 1 Introduction

Protein–protein interactions (PPIs) are central to numerous cellular processes—including immune regulation, signal transduction, and apoptosis—and their dysregulation is strongly associated with diseases such as cancer and drug resistance (Pawson and Nash 2000, Huang 2002, Lee and Yaffe 2016, Bertaux *et al.* 2017, Tsuchiya *et al.* 2022, Zhang *et al.* 2024). Amino-acid substitutions can perturb PPIs by altering interfacial stability or inducing broader conformational shifts, and these effects are commonly quantified by changes in binding free energy (ΔΔG) (Fry and Vassilev 2005, Goncearenco *et al.* 2017, Friedman 2022, Kugler *et al.* 2023). Accurate ΔΔG prediction is therefore critical for understanding disease mechanisms and supporting rational protein and drug design (Cournia *et al.* 2021, King *et al.* 2021, Zhao *et al.* 2022, Zhuravleva *et al.* 2023).

Protein function is jointly governed by sequence and structure (Hegyi and Gerstein 1999, Alberts *et al.* 2002, Sadowski and Jones 2009, Lin *et al.* 2024). While the amino-acid sequence encodes folding and evolutionary constraints, the three-dimensional conformation determines molecular recognition and binding specificity (Jumper *et al.* 2021, Abramson *et al.* 2024). Mutations can disrupt local interface contacts or propagate long-range structural changes that reshape binding affinity (Guerois *et al.* 2002, Dobson 2003, Mahase *et al.* 2024). Conversely, structural constraints exert evolutionary pressure on sequences, particularly at functional interfaces (Kuhlman and Baker 2000, Manhart and Morozov 2015). Capturing these bidirectional sequence–structure dependencies are essential for accurately predicting mutation-induced affinity changes.

Existing ΔΔG prediction strategies fall into two broad categories: energy-based and data-driven approaches. Energy-based models such as BeAtMuSic (2013) and FoldX (Schymkowitz *et al.* 2005) compute energetic perturbations using predefined physical potentials, but often struggle to account for subtle, context-dependent structural effects. Data-driven approaches—including TopGBT (Wang *et al.* 2020), TopNetTree (Wang *et al.* 2020), GeoPPI (Liu *et al.* 2021), MpbPPI (Yue *et al.* 2023), and DGCddg (Jiang *et al.* 2023)—leverage sequence-, distance-, and structure-derived features and thus implicitly reflect sequence–structure relationships. However, these multimodal features are typically fused as loosely coupled input channels, without explicit modeling of cross-dimensional (1D/2D/3D) and cross-scale (residue/atom) interdependencies. Such explicit modeling is critical for capturing the heterogeneous conformational consequences of mutations (Zanzoni *et al.* 2019, Korshunov *et al.* 2023, Tang *et al.* 2023).

Here, we introduce IGMI, an interpretable graph-based framework that explicitly models multi-level dependencies across multidimensional and multiscale protein features, providing a biologically grounded representation of mutation effects. IGMI organizes protein information into a unified graph representation that enables the model to learn how mutation effects propagate across spatial and sequential contexts. Unlike previous approaches that implicitly combine multimodal features, IGMI incorporates explicit modeling of cross-dimensional and cross-scale relationships, allowing the resulting representations to remain biologically grounded and mechanistically coherent. This modeling strategy provides a principled basis for improving mutation-impact prediction while maintaining interpretability.

We extensively evaluate IGMI across multiple benchmark datasets, where it consistently outperforms state-of-the-art data-driven predictors in both single- and multi-mutation scenarios. IGMI exhibits strong robustness under split-by-structure cross-validation and generalizes effectively in blind external validation, demonstrating its ability to model sequence–structure and residue–atom dependencies in novel complexes. Ablation studies confirm the complementary contributions of ProteoMAE and BackSideAttention, and interpretability analyses show that IGMI highlights mutation-proximal residues, interfacial regions, and long-range perturbed sites consistent with established biophysical mechanisms. Additional analyses under different data independence constraints further characterize biologically relevant patterns learned by IGMI for mutation-induced binding affinity changes. Overall, IGMI provides an accurate, interpretable, and biologically grounded framework for predicting mutation-induced changes in protein–protein binding affinity, offering broad utility for protein engineering, antibody design, and therapeutic development.

## 2 Methods

### 2.1 Datasets

**Figure 1:**
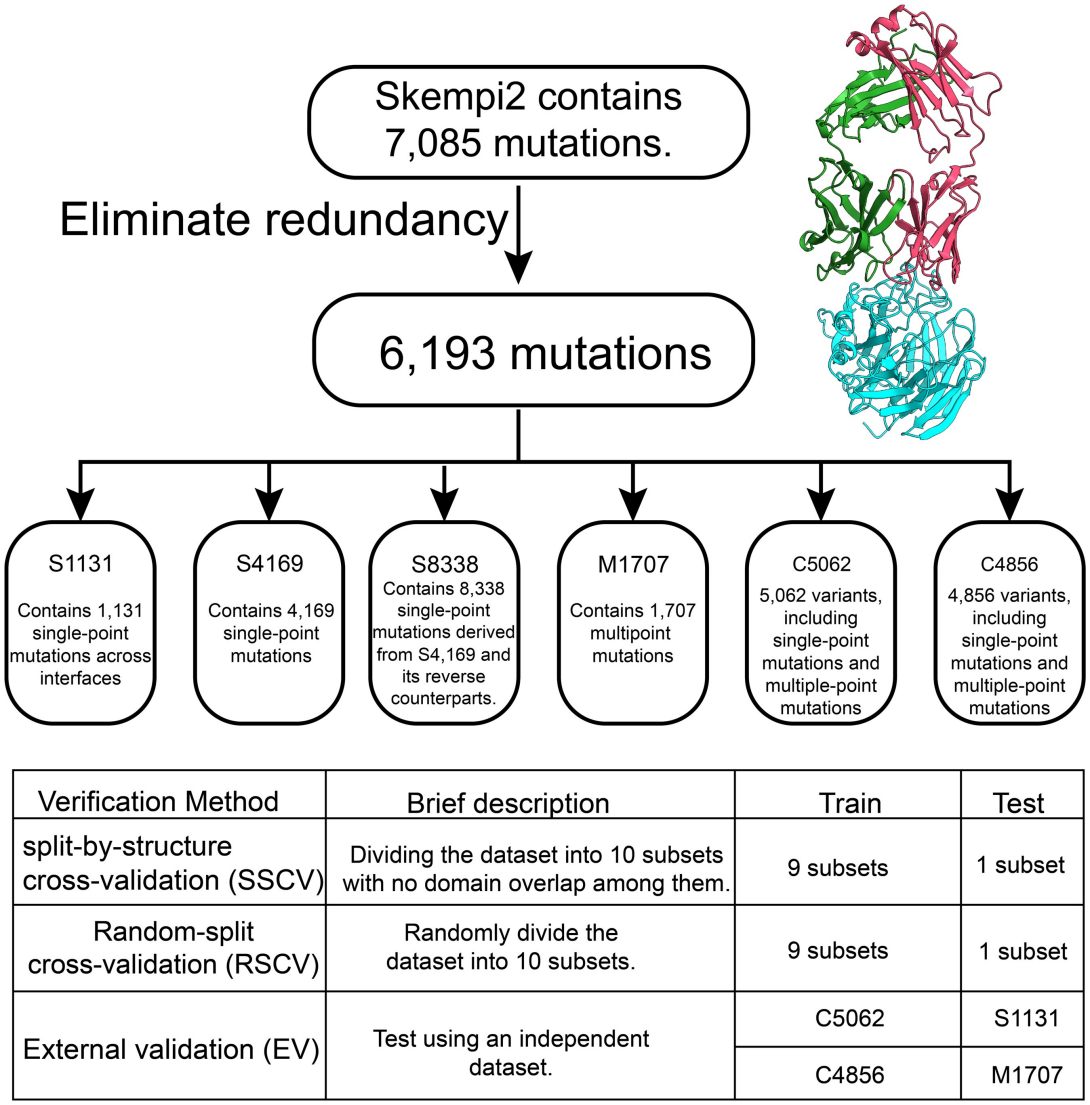
Overview of dataset construction and evaluation protocols.

The four benchmark datasets S1131, S4169, S8338, and M1707 (numbers denote mutation counts) were derived from the SKEMPI 2.0 database (Jankauskaitė *et al.* 2019) (Fig. 1), the largest curated collection of mutation-induced binding affinity changes. SKEMPI 2.0 contains 7,085 mutations across 345 protein complexes, including 6,193 unique variants; for duplicated entries, the averaged ΔΔG was used as ground truth. S1131 comprises 1,131 non-redundant interface single-point mutations (Xiong *et al.* 2017); S4169 contains 4,169 single mutants from 319 complexes (Rodrigues *et al.* 2019); and adding their reverse mutations produces S8338. For multiple mutations, 1,337 variants and their reverses form M1707 (1,707 entries) (Zhang *et al.* 2020).

For external validation, we removed the test entries of S1131 and M1707 from the redundancy-reduced SKEMPI 2.0 set to construct the complementary training datasets C5062 (5,062 variants) and C4856 (4,856 variants).

Each data point includes the wild-type structure, mutation specification, and experimental ΔΔG. Mutant structures were generated via Rosetta Cartesian ΔΔG (Park *et al.* 2016). IGMI takes both wild-type and mutant structures, along with mutation positions, as input for ΔΔG prediction.

### 2.2 Data preprocessing

#### 2.2.1 Dynamic residues selection

To provide a structurally informative yet computationally tractable input, IGMI does not operate on the full protein complex. Instead, we extract a fixed-size subgraph containing 128 residues (denoted as $N_{\mathrm{res}}=128$**)** that

captures the mutation-centered local and semi-global structural context. This subgraph is generated by a dynamic residue selection module that adaptively identifies the region most relevant to mutation-induced affinity changes while maintaining a stable computational footprint with an attention complexity of O ($N_{\mathrm{res}}^{2}$**)**. This design allows IGMI to focus on a consistent and biologically meaningful structural neighborhood across complexes of varying sizes. Details and pseudocode are provided in the Supplementary Materials (Section 1.2.1 and Algorithm 1, available as supplementary data at *Bioinformatics* online).

#### 2.2.2 Feature extraction

We extract multi-level features from each PDB structure by integrating sequence information, residue-level structural descriptors, and atomic-level geometry. Sequence features include residue type and positional encoding. Local structural features are obtained by mapping heavy-atom coordinates into a residue-specific local coordinate system to ensure rotation- and translation-invariance. Global structure is represented using inter-residue Euclidean distances, while atomic-level side-chain geometry is captured using simplified CB-based descriptors. Full definitions and formulas are provided in the Supplementary Materials (Section 1.2.2, available as supplementary data at *Bioinformatics* online).

### 2.3 Model architecture

#### 2.3.1 Protein representation as a graph

We represent each wild-type and mutant protein complex as a graph whose nodes encode residue-level features—including residue type, local heavy-atom geometry, ProtTrans embeddings—and whose edges encode both 1D sequences relationships and 2D contact maps. Atomic-level descriptors are incorporated as bias terms to capture side-chain–backbone geometry. Detailed feature definitions are provided in the Supplementary Materials (Section 1.3.1, available as supplementary data at *Bioinformatics* online).

#### 2.3.2 Protein feature coding

We employ a Transformer-based framework to encode protein representations by integrating 1D sequence information, 2D contact maps, and 3D structural coordinates. Each residue node is characterized using three types of features: amino acid identity, pretrained ProtTrans embeddings, and local heavy-atom geometry. In practice, the ProtTrans embeddings are fused with the learned local geometric descriptor to form a composite residue feature vector, which is subsequently projected into the model’s hidden dimension through a multi-layer perceptron. Global spatial structure is incorporated by introducing distance-based relative positional biases into the attention scores, while 1D sequence distances and residue-pair types are included as additional bias terms. Together, these multidimensional encodings modulate each attention head and allow IGMI to jointly integrate sequence-derived and structure-derived signals during message passing:


(1) $$A_{ij}^{h}=\alpha_{1ij}^{h}-\alpha_{2ij}^{h}+\alpha_{3ij}^{h}$$

Where $\alpha_{1}^{h},\alpha_{2}^{h},\alpha_{3}^{h}\in R^{N_{\mathrm{res}}\times N_{\mathrm{res}}}$ represent the 3D protein structures encoding matrix, 2D contact maps encoding matrix, and 1D sequences encoding matrix of the h-head, respectively. Details and pseudocode are provided in the Supplementary Materials (Section 1.3.2 and Algorithm 2–3, available as supplementary data at *Bioinformatics* online).

#### 2.3.3 ProteoMAE: Multidimensional residue feature aggregation

Protein Multidimensional Residue Feature Aggregation and Excitation Attention (ProteoMAE) is a message-passing unit that operates on the set of correlation-weight matrices derived from the 1D/2D/3D encodings. Given an input set $\alpha =[\alpha_{1},\alpha_{2},\ldots ,\alpha_{C}]$, ProteoMAE learns a transformation to $\alpha^{\prime }=[\alpha_{1}^{\prime },\alpha_{2}^{\prime },\ldots ,\alpha_{C}^{\prime }]$, where $\alpha_{c},\alpha_{c}^{\prime }\in R^{N_{\mathrm{res}}\times N_{\mathrm{res}}\times H}$ denote the original and recalibrated correlation tensors for channel c, respectively. In the baseline formulation (Equation 1), the final attention weights are obtained by element-wise summation of all $\alpha_{c}$, which implicitly encodes inter-matrix dependencies but only in a local, element-wise manner. ProteoMAE instead explicitly models these interdependencies by coupling a global messaging step with an adaptive recalibration step, and then feeding the recalibrated weights into the subsequent graph attention layers. Pseudocode is available in the supplementary materials Algorithm 4, available as supplementary data at *Bioinformatics* online.

##### 2.3.3.1 Global messaging

In Equation 1, each element $A_{\mathrm{ij}}^{h}$ is computed as the sum of the corresponding entries from the correlation matrices of head h, and thus depends only on the residue pair (i,j). As a result, each matrix element is unaware of broader context beyond this pair (Fig. S1a, available as supplementary data at *Bioinformatics* online). To inject global information, we aggregate all edges incident to each residue and compress the spatial dimension of each $\alpha_{c}$into a residue-wise descriptor $z_{c}\in R^{N_{\mathrm{res}}}$. Formally, we compute:


(2) $$z_{c}=F_{sv}(\alpha_{c})=\sum_{i=1}^{N_{\mathrm{res}}}\sum_{h=1}^{H}\alpha_{c}{\;}_{i}^{h}\in R^{N_{\mathrm{res}}}$$

(3) $$z=[z_{1},z_{2},\ldots ,z_{C}]\in R^{C\times N_{\mathrm{res}}}$$

**Figure 2:**
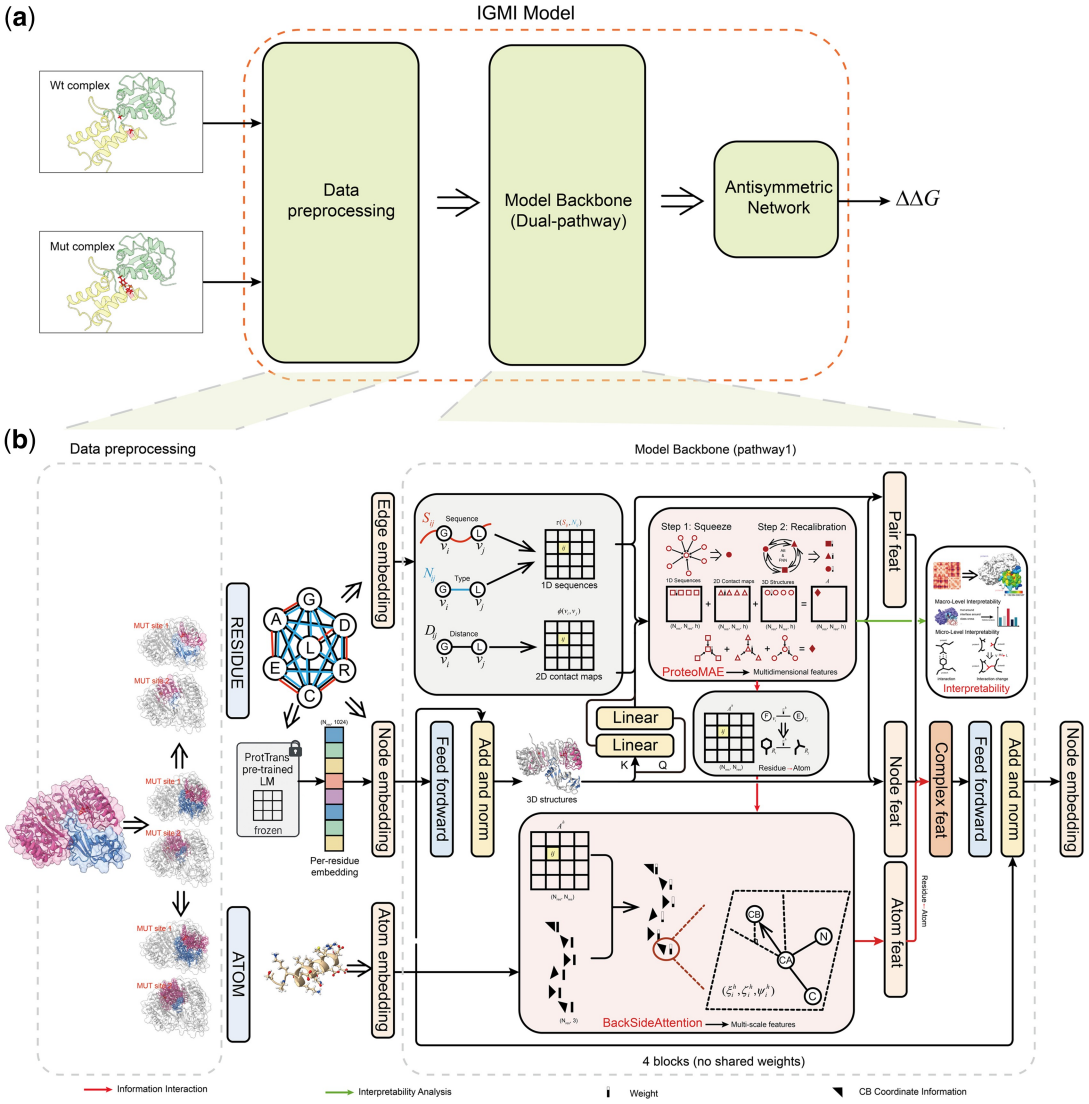
Overview of the IGMI framework. a, Workflow of IGMI, including data preprocessing, ProtTrans-based sequence embedding, dual-pathway encoding of wild-type and mutant complexes, and ΔΔG prediction via an antisymmetric network. b, the model backbone consists of two identical pathways, each integrating multidimensional features (1D sequences, 2D contact maps, and 3D structures augmented with ProtTrans-derived residue embeddings) and multi-scale features (residue- and atomic-level representations) through graph-based encoding. Two key modules further enhance the architecture: ProteoMAE captures global residue dependencies across spatial dimensions, whereas BackSideAttention encodes fine-grained residue–atom interactions, enabling explicit cross-scale information flow.

Where the summations run over residue indices and attention heads. The resulting $z_{c}$summarizes, for each residue, the global correlation pattern encoded in channel c (Fig. 2b).

##### 2.3.3.2 Adaptive recalibration

To exploit the global descriptors z and explicitly model cross-channel dependencies, we introduce an adaptive recalibration mechanism guided by four design goals: (i) the ability to learn nonlinear interactions among correlation matrices; (ii) non–mutually-exclusive weighting (multiple channels can be emphasized simultaneously); (iii) preserving the one-to-one correspondence between channels and learned weights; and (iv) maintaining non-negative weights so as not to invert the logical relationships among encodings.

To this end, we apply a self-attention–based mixing over z, followed by a lightweight gating network:


(4) $$z_{\mathrm{Att}}^{h_{z}}=F_{\mathrm{fusion}}(z)=\mathrm{softmax}\left(\frac{(zW_{q_{z}}^{h_{z}}){\left(zW_{k_{z}}^{h_{z}}\right)}^{T}}{\sqrt{D_{k_{z}}}}\right)zW_{v_{z}}^{h_{z}}\in R^{C\times D_{k_{z}}}$$

(5) $$z_{\mathrm{Att}}={⊕}_{h_{z}=1}^{H_{z}}z_{\mathrm{Att}}^{h_{z}}\in R^{C\times H_{z}\cdot D_{k_{z}}}$$

(6) $$s_{c}=\varsigma \left(\delta \left(z_{\mathrm{Att}}{\;}_{c}W_{1}\right)W_{2}\right)>0$$

(7) $$s=[s_{1},s_{1},\ldots ,s_{C}]\in R^{C\times 1}$$

Where δ(⋅) denotes ReLU, ς(⋅) denotes Softplus, ⊕ denotes concatenation, and $W_{q_{z}},W_{k_{z}},W_{v_{z}}\in R^{N_{\mathrm{res}}\times D_{k_{z}}}$, $W_{1}\in R^{H_{z}\cdot D_{k_{z}}\times N_{\mathrm{res}}}$, $W_{2}\in R^{N_{\mathrm{res}}\times 1}$ are trainable parameters. The self-attentive module with $H_{z}$-heads mixes information across channels, and the two-layer fully connected projection with ReLU and Softplus ensures that the resulting gates are nonlinear and strictly positive.

Finally, the recalibrated correlation weight matrixes are obtained by:


(8) $$\alpha_{c}^{\prime }=F_{\mathrm{scale}}\left(\alpha_{c},s_{c}\right)=s_{c}\alpha_{c}$$

(9) $$A_{ij}^{h}=\alpha_{1}^{\prime }{\;}_{ij}^{h}-\alpha_{2}^{\prime }{\;}_{ij}^{h}+\alpha_{3}^{\prime }{\;}_{ij}^{h}$$

(10) $$\left\{ \begin{array}{c} \alpha_{1}^{\prime }{\;}_{ij}^{h}=F_{\mathrm{scale}}\left(\alpha_{1ij}^{h},s_{1}\right)\\ \alpha_{2}^{\prime }{\;}_{ij}^{h}=F_{\mathrm{scale}}\left(\alpha_{2ij}^{h},\sigma (s_{2})\right)\\ \alpha_{3}^{\prime }{\;}_{ij}^{h}=F_{\mathrm{scale}}\left(\alpha_{3ij}^{h},s_{3}\right) \end{array}\right.$$

Where σ(⋅) denotes the sigmoid function. The gating term prevents uncontrolled amplification of specific channels (e.g. $\alpha_{2}$) and stabilizes training. Taken together, ProteoMAE transforms the original element-wise fusion of correlation matrices into a globally informed, adaptively recalibrated weighting scheme, enabling the graph attention layer to encode richer multidimensional dependencies.

#### 2.3.4 BackSideAttention: Backbone–Sidechain coupled attention

The Backbone-Sidechain Attention Mechanism (BackSideAttention) is designed to establish informative, explicit interactions between residue-based embeddings and side-chain atoms. The module proceeds in two steps: (i) constructing residue-local geometric descriptors through a multi-head 3D β-skeleton, and (ii) externally biasing atom-level attention using residue-level attention weights to enforce cross-scale coupling (Fig. 2b). Pseudocode is available in the supplementary materials Algorithm 5, available as supplementary data at *Bioinformatics* online.

##### *2.3.4.1 3D* β*-skeleton structure*

Side-chain conformations can be described using geometric relationships—orientation, distance, and positional features—defined in a residue-local coordinate frame (e.g., N-Cα-C). Compact or extended $C_{\alpha }-C_{\beta }$ distances reflect rotameric states, while orientation is determined by the backbone direction and secondary-structure context (Fig. S1c, d). These considerations motivate the construction of a residue-local geometric representation, which is later integrated into the attention mechanism.

##### 2.3.4.2 Side chain externally biased attention

To encode side-chain geometry and fuse atomic cues with residue-level context, BackSideAttention maps residue embeddings into Hsubspaces and computes direction (ψ), distance (ζ), and positional (ξ) descriptors for each residue. These quantities are derived using the following operations:


(11) $$f_{\mathrm{MDim}}=\mathrm{softmax}(A)f\in R^{H\times N_{\mathrm{res}}\times 3}$$

(12) $$f_{i}^{h}=R_{i}^{T}(f_{\mathrm{MDim}}{\;}_{i}^{h}-x_{iC_{\alpha }}),h\in \{1,\ldots ,H\},i\in \{1,\ldots ,N_{\mathrm{res}}\}$$

(13) $$\{\begin{array}{c} \xi_{i}^{h}=f_{i}^{h}\in R^{1\times 3}\\ \zeta_{i}^{h}=||f_{i}^{h}||\in R,\;h\in \{1,\ldots ,H\},i\in \{1,\ldots ,N_{\mathrm{res}}\}\\ \psi_{i}^{h}=\frac{f_{i}^{h}}{\left||f_{i}^{h}|\right|}\in R^{1\times 3} \end{array}$$

(14) $$\mathrm{BSA}(f_{i})={⊕}_{h=1}^{H}\left(\xi_{i}^{h}⊕\zeta_{i}^{h}⊕\psi_{i}^{h}\right),i\in \{1,\ldots ,N_{\mathrm{res}}\}$$

where $\mathrm{BSA}(f)\in R^{N_{\mathrm{res}}\times 7H}$ denotes the assembled side-chain geometric code, $R_{i}$is the residue-local Euclidean transformation matrix, $x_{iC_{\alpha }}$and $x_{iC_{\beta }}$ denote backbone and side-chain coordinates, ∥⋅∥ is the vector modulus, and ⊕ denotes concatenation. The resulting geometric descriptors across the Hsubspaces are concatenated to form the final side-chain encoding BSA(f).

Crucially, residue-level attention acts as an external bias to weight these atomic-level features, allowing residue context to modulate side-chain geometry and achieving explicit cross-scale coupling. This ensures bidirectional information flow between residue- and atom-level levels and enables the model to capture mutation-driven structural adjustments.

#### 2.3.5 Antisymmetric network

After feature aggregation, the updated residue, edge, and side-chain features (Equation 14) are concatenated to form a complex-level representation. This representation is processed through a feed-forward block and a residual unit, and the update is repeated four times without parameter sharing. The wild-type and mutant complexes are finally encoded as $u_{\mathrm{wt}},u_{\mathrm{mut}}\in R^{N_{\mathrm{res}}\times 128}$, which are passed to an antisymmetric prediction head to ensure physically consistent ΔΔG estimation.

For residue i, the mutation-induced affinity contribution is computed as:


(15) $$\Delta \Delta G_{i}=\left(\mathrm{FFN}\left(u_{wt}{\;}_{i}⊕u_{\mathrm{mut}}{\;}_{i}\right)-\mathrm{FFN}\left(u_{\mathrm{mut}}{\;}_{i}⊕u_{wt}{\;}_{i}\right)\right)W_{\Delta \Delta G}$$

(16) $$\mathrm{FFN}(p_{i})=\delta \left(\delta \left(\delta \left(p_{i}W_{1}+b_{1}\right)W_{2}+b_{2}\right)W_{3}+b_{3}\right)$$

Where ⊕ denotes concatenation, and $W_{1},W_{2},W_{3},W_{\Delta \Delta G}$ and $b_{1},b_{2},b_{3}$ are trainable parameters (dimensions listed in the Supplementary Materials Section 1.3.3, available as supplementary data at *Bioinformatics* online). The prediction module consists of a four-layer feed-forward network with ReLU activations, applied identically to both $u_{\mathrm{wt}}$ and $u_{\mathrm{mut}}$.

The final complex-level ΔΔG is obtained by aggregating residue-level contributions:


(17) $$\Delta \Delta G=\sum_{i=1}^{N_{\mathrm{res}}}\Delta \Delta G_{i}$$

This antisymmetric design introduces a natural sign-equivariance: exchanging the wild-type and mutant encodings leads to a corresponding sign change in the predicted ΔΔG, reflecting the expected physical relationship between forward and reverse mutations.

### 2.4 Training and evaluation

For split-by-structure cross-validation (SSCV) and random-split cross-validation (RSCV), we adopted a unified 8:1:1 data-splitting strategy, dividing each dataset into training, validation, and test subsets. For external validation (EV), the test sets S1131 and M1707 were fixed a priori, and their complementary subsets in SKEMPI 2.0 (C5062 and C4856, respectively) were used as the training data. Each EV training set was then partitioned into a 9:1 ratio to form the training and validation subsets. Hyperparameters were selected via grid search over dropout∈{0, 0.3, 0.5, 0.7}, batch size∈{32, 64, 128}, and $\mathrm{learning}\;\mathrm{rate}\in \{5\times 10^{-5},\;1\times 10^{-5},\;1\times 10^{-4}\}$. Each configuration was trained for 500 epochs, and the combination achieving the lowest validation loss was used for subsequent training.

After hyperparameter selection, the IGMI model was trained for 20,000 epochs, and the epoch achieving the lowest validation loss was identified. The model was then retrained from scratch on the full training dataset (combining both the training and validation subsets) up to the selected epoch. This inner validation split procedure prevents information leakage while leveraging all available data. Training used MSE loss and the Adam optimizer, with the learning rate halved when no improvement in training loss was observed over ten consecutive epochs (checked every 100 epochs). All parameters were trained from scratch, and ProtTrans was used only to provide fixed residue-wise embeddings (Details are provided in the Supplementary Materials Section 1.3.2.1, available as supplementary data at *Bioinformatics* online). To ensure comparability within each training session, a fixed seed was used during individual runs.

To enhance data diversity, we applied reverse-mutation augmentation: for each training sample, a paired sample was generated by swapping wild-type and mutant structures and negating the corresponding ΔΔG. This strategy doubled the training set while preserving structural geometry.

IGMI was evaluated under three protocols (Fig. 1). (i) RSCV: random partitioning into ten folds, each used once as the test set. (ii) SSCV: following the evaluation protocol adopted in GeoPPI, the dataset was partitioned into ten non-overlapping structure-based folds, such that each protein complex appears in exactly one fold and no Evolutionary Classification of Protein Domains (ECOD) (Cheng *et al.* 2014)-defined structural domains are shared between the training and test sets. Fold sizes were balanced using a greedy partitioning algorithm (Supplementary Materials, Algorithm 6, available as supplementary data at *Bioinformatics* online). (iii) EV: for S1131 and M1707, all corresponding test mutations were removed from SKEMPI 2.0, leaving 5,062 and 4,856 variants, respectively, for training (C5062 and C4856). Detailed fold assignments are provided in the Supplementary materials S4 and S5 Table.

All experiments were performed in PyTorch 2.9 (CUDA 12.6) on Ubuntu 22.04.2 LTS with a single NVIDIA RTX 4090 GPU. Training each model required approximately two weeks. Performance was assessed using Pearson correlation (Rp), root-mean-square error (RMSE), and mean absolute error (MAE). Statistical significance and confidence intervals were obtained from ten repeated runs. Definitions, formulas, and implementation details are provided in the Supplementary materials Section 1.4, available as supplementary data at *Bioinformatics* online.

To ensure fair and directly comparable evaluation, all baseline methods were reproduced under the same computational environment as IGMI (CUDA 12.6, NVIDIA RTX 4090 GPU, Intel i9 CPU, Ubuntu 22.04.2 LTS). Data-driven baselines—including GeoPPI, TopGBT, TopNetTree, MpbPPI, DGCddg and MutaBind2—were executed using their official implementations, with identical dataset splits, and evaluation metrics. For classical energy-based tools that do not support model retraining (e.g., FoldX and BeAtMuSic), we used the official binary distributions and applied them to the same processed structural inputs. This unified reproduction protocol eliminates variability introduced by hardware, software, or data-handling differences, ensuring that all reported results are directly comparable. The reproduction code for baseline methods has been organized and uploaded to Google Drive (https://drive.google.com/drive/folders/1CYxd-utnrIKLUtyZ-EfTCUX6WbGRRn8E? usp=sharing). Detailed reproduction procedures, environment configurations, and execution scripts are provided in the Supplementary Materials Section 3, available as supplementary data at *Bioinformatics* online.

## 3 Results

### 3.1 Model performance for PPIs

**Figure 3:**
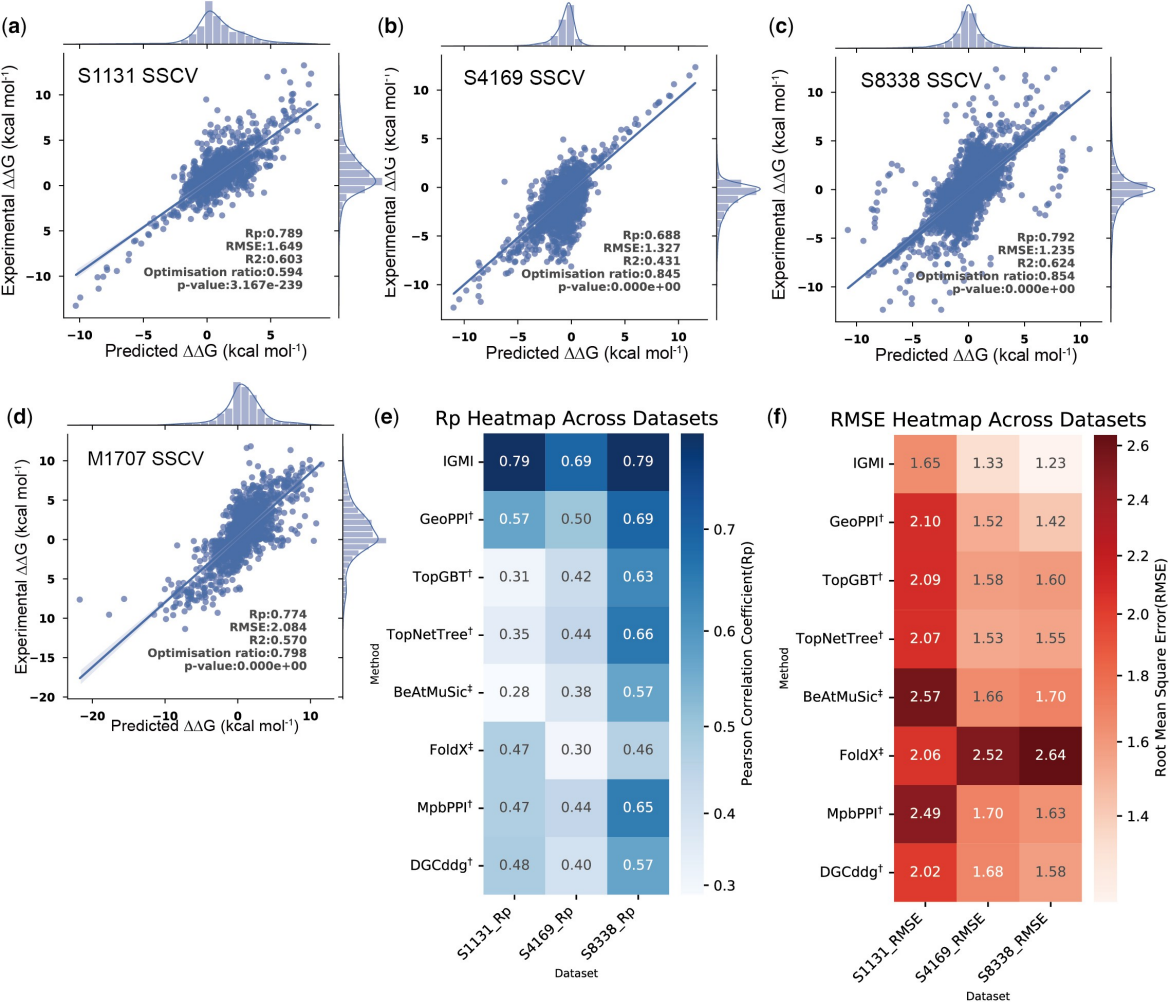
IGMI performance on protein complex affinity prediction. (a–d) Predicted vs experimental ΔΔG and Pearson correlation (Rp) on S1131/S4169/S8338/M1707 under split-by-structure cross-validation (SSCV; ECOD-based, 10 non-overlapping folds; no shared protein domains). The Optimization ratio refers to the proportion of samples for which the increase in binding affinity is correctly predicted, relative to all samples predicted to show an increase in binding affinity (See ablation study). (e, f) Method comparison on S1131/S4169/S8338 in terms of Rp and RMSE under the same SSCV protocol. ^**†**^Results were obtained based on the released source code. ^**‡**^Results were obtained via the released tool.

We first compared IGMI with competitive methods under the SSCV protocol on the single-mutation datasets S1131, S4169, and S8338 (Fig. 3e and f). Empirical energy-based approaches such as BeAtMuSiC and FoldX yielded the lowest correlations and highest RMSE values, consistent with their limited ability to model context-dependent structural effects. Data-driven methods, including TopGBT and TopNetTree, achieved improved performance by learning from mutational features. Deep learning–based models (GeoPPI, MpbPPI (Yue, *et al.* 2023), DGCddG(Jiang, *et al.* 2023), and IGMI) further improved predictive accuracy across datasets, reflecting the advantage of data-driven architectures in capturing nonlinear patterns in PPIs. Across all datasets, IGMI achieved the highest performance. Compared with the previous state-of-the-art method GeoPPI, IGMI improved the Pearson correlation coefficients by 38.6% (from 0.57 to 0.79) and 38% (from 0.50 to 0.69) on S1131 and S4169, respectively. Prior deep learning models generally integrate sequence-derived and structure-derived features and can therefore implicitly capture certain sequence–structure relationships. However, these modalities are typically processed independently or fused through simple concatenation, without explicitly modeling their multidimensional and multiscale interdependencies. In contrast, IGMI explicitly couples 1D sequences, 2D contact maps, and 3D structures through global message aggregation and adaptive recalibration, enabling it to capture mutation-induced perturbations more comprehensively (Fig. 3a–c).

We also evaluated IGMI on the multi-mutation dataset M1707, where it consistently outperformed GeoPPI, MutaBind2, and FoldX in both Pearson correlation and RMSE (Table 1; Fig. 3d). Interestingly, GeoPPI performed better on multi-mutation than on single-mutation datasets (0.73 on M1707 vs. 0.57 on S1131), whereas IGMI maintained strong performance across both settings (0.77 on M1707 vs. 0.79 on S1131). This pattern underscores the importance of explicitly modeling multi-level feature interdependencies, particularly when representing subtle or higher-order mutational effects. To ensure completeness, we also report results under conventional RSCV, in which training and test sets may share highly similar protein complexes. Under this setting, IGMI remains competitive, achieving top-tier performance on several datasets and performance comparable to leading methods on others (Tables S1–S2), thereby demonstrating its effectiveness across different evaluation protocols.

**Table 1:** Performance comparison on the multi-mutation dataset M1707 (SSCV protocol).

Method	M1707	
	Rp	RMSE
IGMI	**0.77**	**2.08**
GeoPPI^†^	0.73	2.15
MutaBind2^†^	0.71	2.31
FoldX^**‡**^	0.51	2.95

Rp: Pearson correlation; RMSE: root-mean-square error. ECOD-based SSCV ensures that test complexes share no domains with the training data. ^†^Results were obtained based on the released source code. ^‡^Results were obtained via the released tool. Bold values indicate the best performance among the compared models.

**Figure 4:**
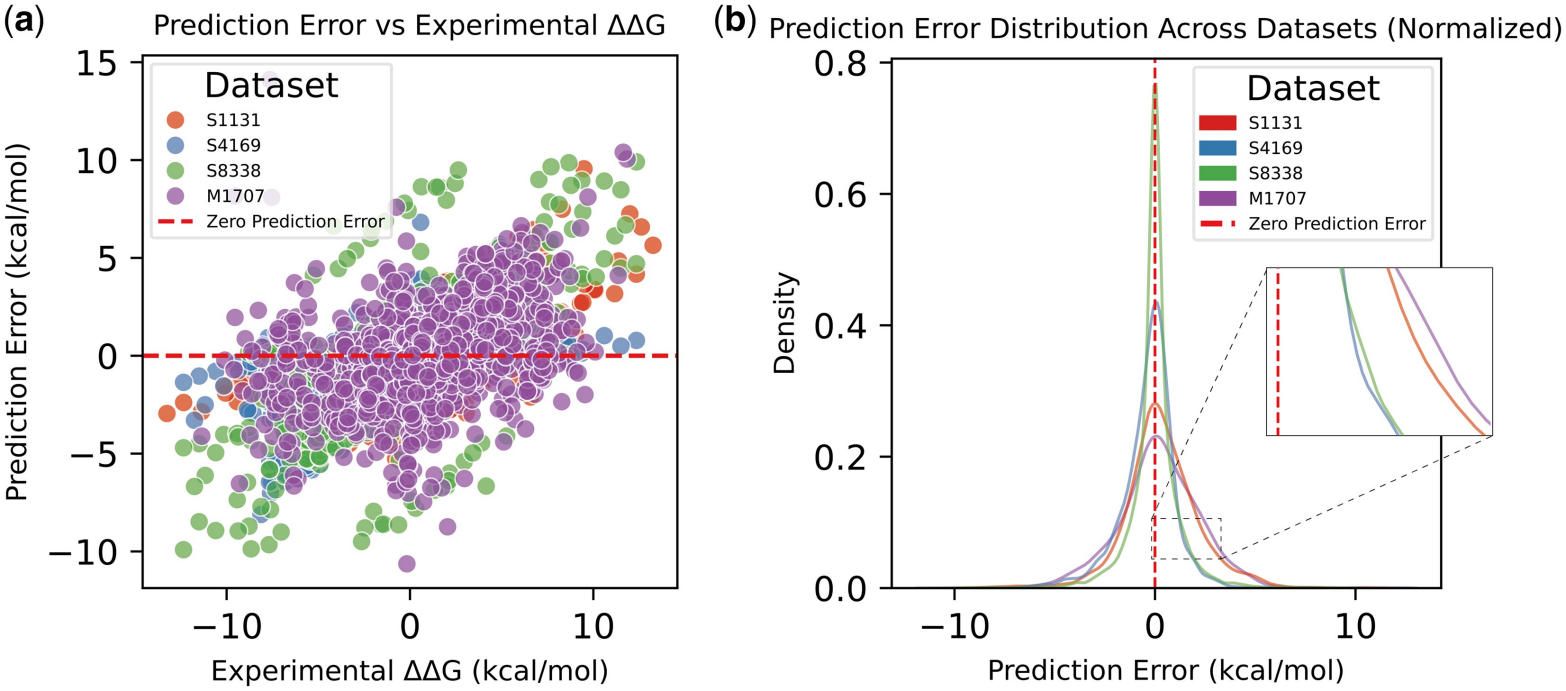
Prediction error analysis of IGMI across different datasets. (a) Prediction error versus experimental ΔΔG for S1131, S4169, S8338, and M1707. (b) Normalized error distributions for each dataset.

Finally, we analyzed prediction-error distributions across all datasets (Fig. 4a and b). Most predictions lie close to the zero-error line, and normalized error distributions are centered near zero with no systematic bias. Single-mutation datasets (S1131, S4169, S8338) exhibit narrower peaks, indicating higher stability, whereas M1707 shows broader variability, reflecting the increased complexity of multi-mutation patterns. Overall, IGMI demonstrates robust and consistent behavior across evaluation settings.

### 3.2 External validation

**Figure 5:**
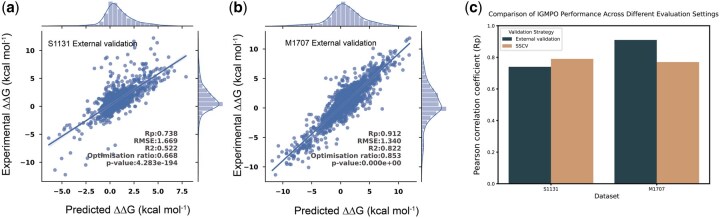
Performance of IGMI in external validation. (a, b) Predicted vs experimental ΔΔG on S1131 and M1707 as blind test sets. (c) Comparison of evaluation settings.

To further assess generalization, we performed blind prediction on two SKEMPI-derived subsets: S1131 (single-point) and M1707 (multi-point). For each test set, all corresponding mutations were removed from SKEMPI 2.0, and IGMI was trained on the remaining entries (Fig. 1). This protocol evaluates robustness under broader and more heterogeneous conditions than SSCV, which enforces domain-level separation (Fig. 5).

Distinct trends emerged across the two datasets. On S1131, IGMI achieved Rp = 0.74—slightly lower than the SSCV score (0.79)—likely reflecting reduced specificity for interface-centered single-point mutations when the training set contains mixed mutation types. In contrast, on M1707, IGMI reached Rp = 0.91, markedly higher than under SSCV (0.77). The broader and more diverse training set in the blind-test setting appears to better capture the higher-order, non-additive effects characteristic of multi-point mutations, enabling stronger generalization to complex mutation patterns.

Together, these results underscore the importance of evaluating ΔΔG predictors under both structurally controlled (SSCV) and real-world (blind testing) scenarios. SSCV enforces strict structural independence, whereas external validation probes practical robustness across heterogeneous mutation distributions. IGMI’s strong performance across both settings highlights the advantage of explicit multi-level feature–interaction modeling, which is particularly valuable for applications such as protein design, drug-resistance prediction, and antibody optimization.

### 3.3 Ablation study

We conducted an ablation study to quantify the contribution of IGMI’s key components. To ensure robustness, all variants were evaluated on an independent blind test set (S1131), following the external validation protocol.

**Figure 6:**
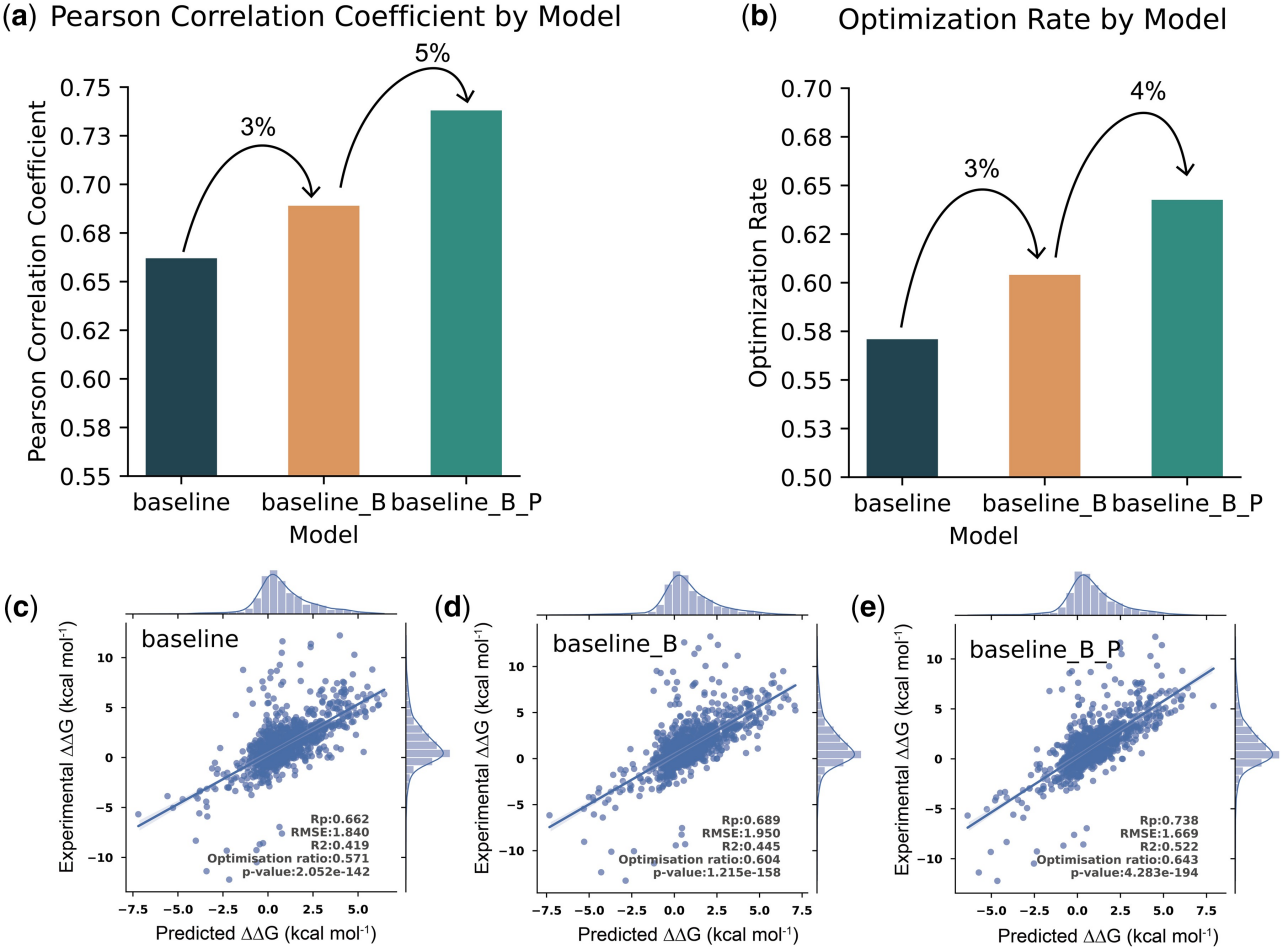
Ablation results of IGMI. (a) Bar chart of Pearson correlation (Rp) as modules are added sequentially to the baseline: +3% with BackSideAttention, then +5% with ProteoMAE. (b) Bar chart of Optimization Ratio under the same sequence: +3% with BackSideAttention, then +4% with ProteoMAE. (c–e) Scatter plots for each ablation model corresponding to the configurations in a–b.

Integrating BackSideAttention and ProteoMAE yields clear and incremental performance gains (Fig. 6). Relative to the baseline (Rp = 0.66), adding BackSideAttention improves correlation to 0.69, and incorporating both modules further boosts performance to Rp = 0.74 with an increase in R^2^ from 0.42 to 0.52. Error metrics similarly improve: RMSE decreases from 1.83 to 1.67, and MAE from 1.14 to 1.06 (Table 2), indicating more accurate mutation-effect prediction.

**Table 2:** Summarizes the performance of three model configurations: the baseline, baseline with BackSideAttention (baseline_B), and baseline with both BackSideAttention and ProteoMAE (baseline_B_P).

Method	Rp	R2	RMSE	MSE	MAE	P-value	Rp-confidence interval (95%)
baseline	0.66	0.42	1.84	3.38	1.14	2E-142	(0.63,0.69)
baseline_B	0.69	0.44	1.95	3.80	1.18	1E-158	(0.66,0.72)
baseline_B_P	**0.74**	**0.52**	**1.67**	**2.79**	**1.06**	**4E-194**	**(0.71, 0.76)**

Bold values indicate the best performance among the compared models.

To assess optimization ability, we introduced the Optimization Ratio, which measures the proportion of correctly predicted affinity-enhancing mutations among those predicted to increase affinity. As shown in Fig. 6b, BackSideAttention improves the Optimization Ratio by 3%, and adding ProteoMAE provides an additional 4% gain.

These results highlight the complementary roles of the two modules. ProteoMAE enhances performance by modeling interdependencies among 1D sequence, 2D contact maps, and 3D structures through structured multidimensional feature recalibration. BackSideAttention strengthens residue–atom coupling via cross-scale attention, allowing finer-grained geometric cues to influence residue-level representations. Together, these mechanisms enable IGMI to capture critical determinants of protein binding affinity across spatial scales and hierarchical levels.

Overall, the ablation study demonstrates that explicitly modeling multi-level feature interactions substantially improves predictive accuracy and the ability to identify affinity-enhancing mutations, reinforcing the practical value of IGMI for protein design and affinity optimization.

### 3.4 Interpretability and visualization of the model

**Figure 7:**
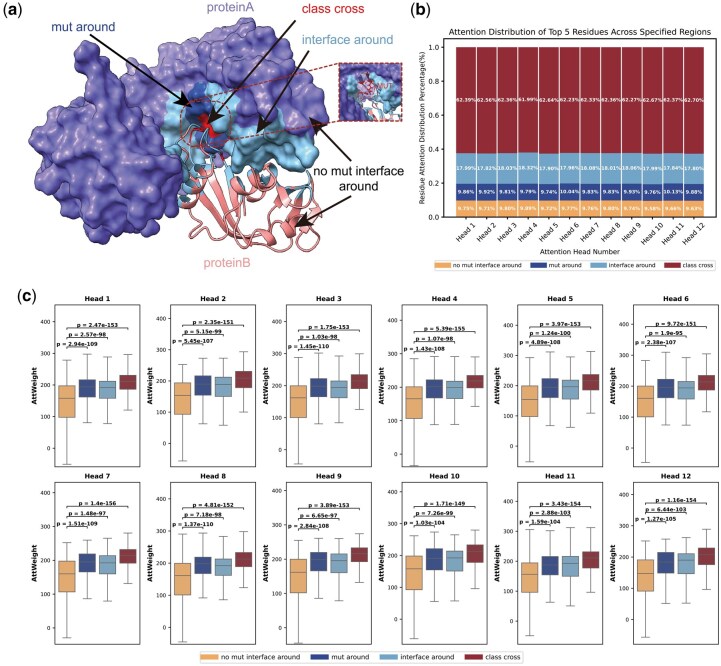
Interpretability analysis of attention weights at the macro level. (a) Protein residues are categorized into four different types based on the form of their interactions. (b) Distribution of the number of attentional weights Top5 residues in each attention head across regions. (c) Distribution of weight values of attentional weight Top5 residues in different regions in each attention header.

IGMI predicts mutation-induced affinity changes by modeling multi-level feature interactions within 3D conformations. Since attention weights can reflect residue-level contributions in PPIs (Liu, *et al.* 2023), we analyzed IGMI’s attention distributions to examine whether it captures known biophysical mechanisms, including (i) direct interface effects, where mutations alter local contacts (Ammar, *et al.* 2023, Grassmann, *et al.* 2023), and (ii) long-range effects, where perturbations propagate through the structure to affect global stability (LiCata and Ackers, 1995, Bigman and Levy, 2018). Residues were grouped into four mutually exclusive regions (Fig. 7a):

- Mut Around: Residues within 5 Å of the mutation site (Ovchinnikov *et al*. 2014)
- Interface Around: Interface residues defined following Levy et al. (Levy 2010)
- Class Cross: Residues belonging to both categories above
- No Mut Interface Around: Remaining residues

#### 3.4.1 Macro-level interpretability: affinity-relevant regions prioritized by IGMI

Across S1131 and M1707, we mapped the top-5 attention residues from each complex into the four regions (28,380 residues total). As shown in Fig. 7b, IGMI strongly prioritizes interface-related areas: on average, 62.40% of high-attention residues fall in Class Cross, while 17.98% fall in Interface Around. Attention to Class Cross is more than threefold higher than Interface Around, indicating high sensitivity to interaction changes occurring directly at the mutated interface. Meanwhile, 9.88% of high-attention residues lie in Mut Around, indicating that IGMI may also captures mutation-propagated, non-interface effects.

Beyond counts, t-tests and box plots (Fig. 7c) show significantly higher attention in Class Cross and Interface Around compared to No Mut Interface Around across all attention heads, with Class Cross consistently highest. Mut Around also exhibits higher weights than No Mut Interface Around, further supporting the model’s ability to detect indirect structural perturbations.

#### 3.4.2 Micro-level interpretability: IGMI captures interaction reorganization after mutation

We next analyzed IGMI’s attention within individual complexes.

**Figure 8:**
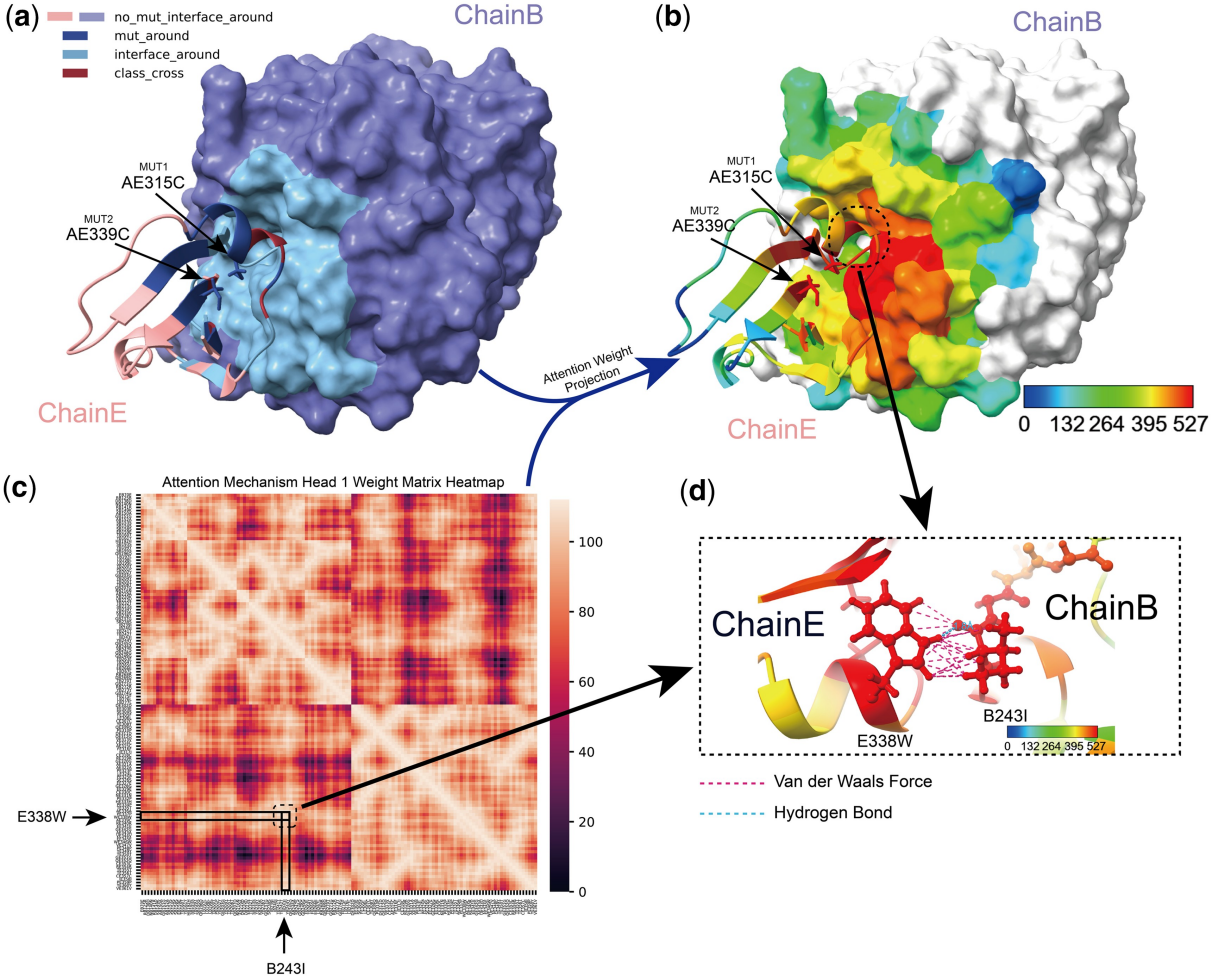
IGMI can capture interactions between proteins at the micro level. (a) Residue region classification of the Carboxypeptidase A1 (Chain B) - Metallocarboxypeptidase Inhibitor (Chain E) complex. (b) Head-1 attention weights mapped onto the 3D structure. (c) Head-1 attention weight matrix heatmap; residues are ordered by 1D sequence with Chain E concatenated to Chain B. Quadrants II/IV show intra-protein correlations; quadrants I/III show inter-protein correlations. d, Zoom-in of model-focused regions: strong hydrogen bonds and multiple van der Waals contacts form between the two highest-attention residues on Chains E and B via side chains. Note: AE315C denotes Chain E residue 315 mutates Alanine (Ala)→Cysteine (Cys); E338W denotes Chain E residue 338 is Tryptophan (Trp).

For the Carboxypeptidase A1–Metallocarboxypeptidase Inhibitor complex with non-interface mutations AE339C and AE315C, IGMI’s highest attention values (∼527) concentrate at the interface and mutation-proximal regions (Fig. 8), consistent with macro-level trends. Inspection of the two top-ranked residues (E338W, B243I) shows that, although distant along the backbone, they form hydrogen bonds and van der Waals contacts via side chains—indicating that IGMI captures fine-grained, side-chain–mediated interactions, likely via BackSideAttention. Heatmap ordering along the concatenated 1D sequences (Fig. 8c) reveals stronger correlations near the diagonal, suggesting heightened attention to sequence-proximal residue pairs.

**Figure 9:**
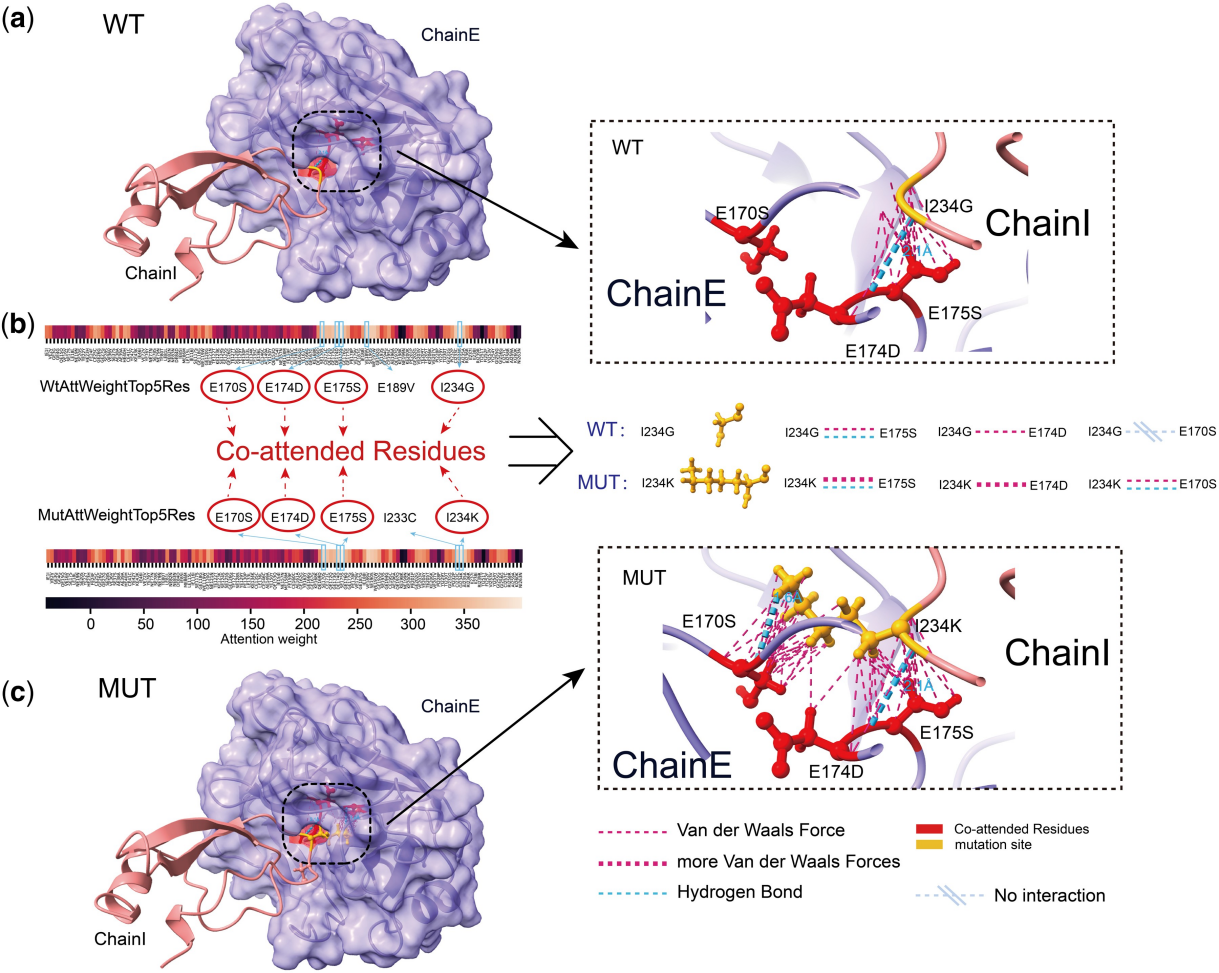
IGMI can capture changes in interactions between side chain atoms before and after mutation at the microscopic level. (a, c) Visualization of Co-attended Residues in Wild-Type and Mutant Attention-Weighted Top5 Residues on Protein Structures. (b) Co-attended Residues are all altered to interact with the surrounding residues to varying degrees upon mutation. Note: I234G indicates that residue id234 on ChainI is Glycine (Gly).

To examine mutation-induced changes, we compared attention patterns before and after mutation in the Trypsin–Pancreatic Trypsin Inhibitor complex (GI234K). The co-attended residues (E170S, E174D, E175S, I234G/I234K) show clear shifts: substitution G→K at residue 234 strengthens van der Waals interactions with E174S and E175D and introduces new hydrogen bonds with E170S (Fig. 9). These observations confirm that IGMI detects local and long-range interaction reorganization driven by mutation, especially within side-chain atomic contacts, consistent with the bidirectional residue–atom information flow enabled by BackSideAttention.

Overall, IGMI effectively highlights interface and mutation-proximal residues at the macro level and detects mutation-induced side-chain interaction rearrangements at the micro level—including hydrogen-bond and van der Waals reorganization. This interpretability likely arises from ProteoMAE’s multidimensional feature recalibration and BackSideAttention’s cross-scale coupling, enabling the model to selectively amplify biologically informative signals and accurately predict mutation-driven affinity changes.

### 3.5 What does the model actually learn? Insights from data splitting strategies

Previous studies have shown that random interaction-level splitting can substantially overestimate model performance in protein–protein interaction research due to information leakage, particularly when highly similar protein sequences or closely related protein complex structures appear in both training and test sets (Bernett *et al.* 2024). This issue is especially relevant for structure-based prediction tasks. In our study, each data point corresponds to a mutation within a specific protein–protein complex, and the model explicitly leverages complex-level structural information. As a result, information leakage may arise not only from protein identity overlap, but also from structural similarity between protein complexes, particularly at the domain level.

Based on these considerations, we adopt SSCV as our primary evaluation protocol. SSCV partitions data at the protein-complex level and explicitly minimizes structural-domain similarity between training and test folds using the ECOD database. This protocol, originally proposed in GeoPPI to mitigate structural overlap in structure-based protein interaction modeling, effectively reduces complex-level structural leakage and ensures fold independence with respect to structural information, which is essential for fair evaluation in structure-based ΔΔG prediction.

From a complementary perspective, overlap of protein partners between training and test sets may also introduce information leakage (Bernett *et al.* 2024). To assess model behavior under a protein-partner–level independence constraint, we additionally performed a protein-partner independent splitting experiment following the C3 definition (Park and Marcotte 2012) (C3CV), in which no protein partners appearing in the test set are present in the training set; different variants involving the same protein partners were treated as identical.

**Figure 10:**
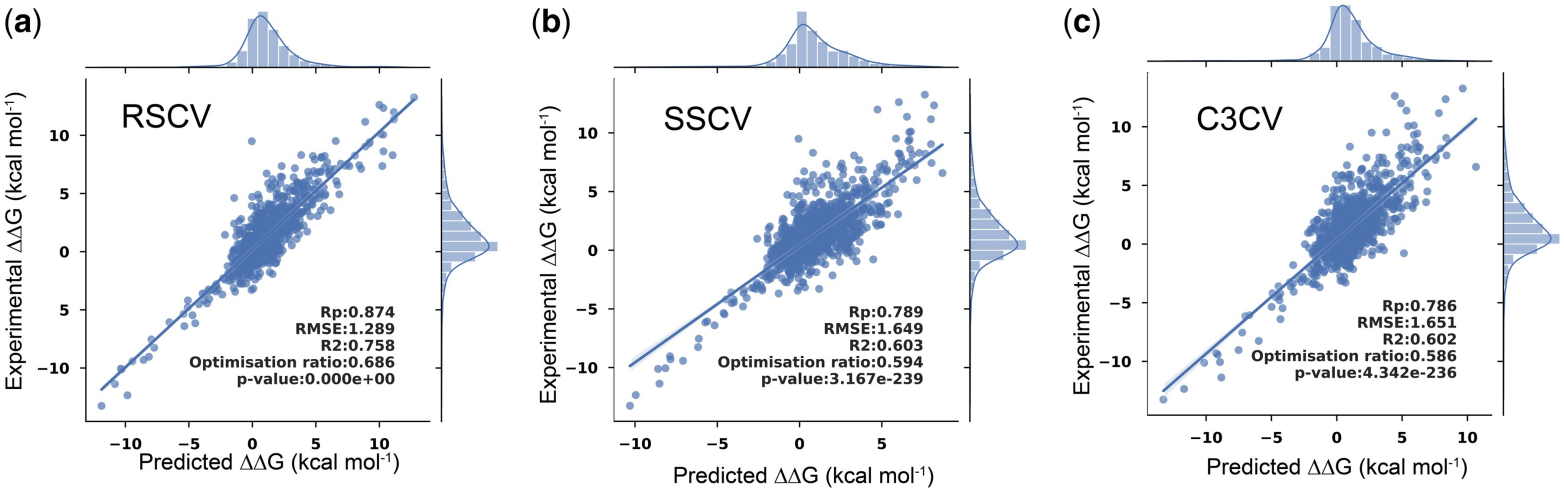
Performance comparison of the proposed model under different data splitting strategies on the S1131 dataset. (a) RSCV. (b) SSCV. (c) C3CV.

Using the S1131 dataset as an illustrative example (Fig. 10), RSCV yields the highest apparent performance (Rp = 0.87), whereas performance decreases under both SSCV (Rp = 0.79) and C3CV (Rp = 0.79). The comparable performance under SSCV and C3CV indicates that enforcing independence at either the structural-domain level or the protein-partner level yields consistent performance estimates, in contrast to the inflated results obtained under random splitting.

Taken together, these results indicate that the predictive performance of our model is not driven by data partition–specific information leakage. Instead, the robustness of performance across distinct independence constraints suggests that the model learns generalizable and biologically meaningful patterns underlying mutation-induced binding affinity changes. Notably, SSCV imposes a stricter evaluation criterion than C3, as structurally similar protein complexes may still exist even when protein partners are entirely distinct. Therefore, we consider SSCV to provide a more rigorous and fair assessment of model performance for structure-based ΔΔG prediction. When considered alongside the interpretability analyses presented earlier, these findings further support that the model’s effectiveness arises from its ability to capture affinity-related interactions within protein complexes, rather than exploiting dataset-specific shortcut strategies.

## 4 Discussion

Mutation-induced changes in protein affinity are jointly determined by both sequence and structure, as mutations may alter local interactions at the interface or trigger broader conformational adjustments (Chi and Liberles, 2016). Therefore, accurately predicting ΔΔG requires capturing the bidirectional dependencies between sequence and structure. However, many existing methods treat sequence and structure as independent inputs (Wang, *et al.* 2020, Liu, *et al.* 2021, Jiang, *et al.* 2023, Yue, *et al.* 2023), limiting their ability to model the biophysical mechanisms underlying mutation effects.

IGMI’s architecture explicitly models cross-dimensional dependencies by enforcing structured message passing across sequence-, distance-, and coordinate-derived representations, enabling the integration of local atomic geometry with global spatial context. IGMI integrates two key modules: ProteoMAE, which recalibrates multidimensional residue features to model sequence–structure dependencies, and BackSideAttention, which couple’s residue- and atom-level representations through cross-scale attention. Together, these mechanisms enable IGMI to learn long-range and fine-grained geometric signals relevant to binding affinity.

Across multiple benchmark datasets, IGMI achieves consistent and robust performance in both single- and multi-mutation settings. Under the stringent SSCV protocol—which enforces domain-level separation to emulate unseen structures—IGMI significantly outperforms existing deep learning and machine learning baselines, demonstrating strong generalization. Its stable performance in external validation further suggests that IGMI captures biologically meaningful patterns beyond data-specific biases.

Ablation experiments confirm the importance of IGMI’s architectural components: removing either ProteoMAE or BackSideAttention leads to clear declines in accuracy and optimization ratio. Their combined contributions highlight the necessity of explicitly modeling multi-level feature interactions to capture the energetic and structural determinants of PPIs, particularly for identifying affinity-enhancing mutations.

Crucially, IGMI provides interpretable insights aligned with biophysical principles. At the macro level, it prioritizes interface and mutation-proximal regions and identifies long-range perturbations in non-interface residues. At the micro level, IGMI captures mutation-induced reorganization of hydrogen bonds and van der Waals contacts at the side-chain level. These interpretable behaviors differentiate IGMI from existing black-box ΔΔG predictors (Wang, *et al.* 2020, Jiang, *et al.* 2023, Yue, *et al.* 2023) and demonstrate that the model’s decisions are grounded in structural mechanisms.

Beyond predictive accuracy and interpretability, an important question is what the model learns from protein complex data. Analyses under different data independence constraints show consistent behavior across structurally constrained and protein-partner–independent settings, suggesting that IGMI captures interaction patterns intrinsic to mutation-induced binding affinity changes rather than partition-specific correlations. This observation is particularly relevant for structure-based ΔΔG prediction, where structural similarity between complexes can otherwise lead to overly optimistic evaluation.

In summary, IGMI offers a unified and interpretable framework for modeling mutation impacts by incorporating explicit multidimensional and multiscale feature interactions. Beyond ΔΔG prediction, the representations learned by IGMI hold potential for broader applications, including macromolecular docking, antibody engineering, and rational protein design. This work advances structure-aware machine learning for PPIs and enhances our ability to model mutation-induced perturbations with both accuracy and interpretability.

## Supplementary Material

btag150_Supplementary_Data

## Data Availability

The source code for IGMI is publicly available at our GitHub repository: https://github.com/ShiweiWu-545/IGMI.git This repository contains the full implementation of the IGMI framework, including model architectures, training scripts, and evaluation utilities. To ensure complete reproducibility of all results presented in this study, we additionally provide an archived collection on Zenodo: https://doi.org/10.5281/zenodo.17563574 The Zenodo archive includes three ZIP packages: IGMI Full Version.zip: the full reproducibility package containing all source code, pre-trained model weights, processed datasets, and example scripts required to reproduce all experiments and figures in the manuscript. prottrans.zip: supplementary ProtTrans weight files used for initializing the sequence-embedding component of IGMI (excluded from GitHub due to size constraints). Skempi2_ddg_useful.zip: the processed SKEMPI 2.0–derived dataset used for model training and evaluation, including Rosetta-generated mutant and wild-type complex structures.
