# Supplementary material for: Enhancing mutation impact prediction in protein-protein interactions through interpretable graph-based multi-level feature interactions
Source: Bioinformatics. 2026 Mar 27;42(4):btag150. doi: 10.1093/bioinformatics/btag150 (PMC13070472; doi:10.1093/bioinformatics/btag150)
Supplement: btag150_Supplementary_Data [file btag150_supplementary_data.zip › 03-Apr-2026_090732_Supporting_Information.pdf]

Supporting Information for

Enhancing Mutation Impact Prediction in Protein-Protein Interactions through Interpretable Graph-Based Multi-Level Feature Interactions

Shiwei Wu[1], Nan Xu[2,3], Xiaohui Xin[1], Min Zhang[1], HaoLiang Liu[1], Hongjia Zhu[2,3], Zhenyu Wei[1], Chengkui Zhao[1,3*], Lei Yu[2,3*], Weixing Feng[1*]

[1]College of Intelligent Systems Science and Engineering, Harbin Engineering University, Harbin, China, [2]Institute of Biomedical Engineering and Technology, Shanghai Engineering Research Center of Molecular Therapeutics and New Drug Development, School of Chemistry and Molecular Engineering, East China Normal University, Shanghai, China, [3]Shanghai Unicar-Therapy Bio-medicine Technology Co., Ltd, Shanghai, China

*To whom correspondence should be addressed.

This PDF file includes:

1 Detail Methods
2 Pseudocode
3 Baseline Reproduction Details
4 Supplementary Tables S1 to S7
5 Supplementary Figures S1 to S3
6 SI References

1 Detail Methods

1.1 The model overview

This section introduces the architecture of IGMI (Fig.2), a novel interpretable graph-based multi-level feature interaction model for mutation impact prediction. IGMI integrates multi-level features, including the 1D sequences layer, 2D contact maps layer, 3D structures layer, and atomic-based side-chain geometric features, to effectively capture the inherent complex dependencies in PPI data, thereby improving the accuracy of $\Delta\Delta G$ predictions. The core of the IGMI architecture lies in two variant network modules based on attention mechanisms. These modules are designed to model the interdependencies and information flow across multidimensional features, including 1D sequences, 2D contact maps, and 3D structures features, as well as multi-scale features such as residue- and atomic-based information. By integrating these multidimensional and multi-scale features, IGMI effectively models the intricate relationships between sequence and structural characteristics.

1.2 Data Preprocessing

1.2.1 Dynamic residues selection

Most existing studies suggest that information related to binding affinity changes is primarily concentrated in the contact surface region of protein complexes[1]. However, as mutation sites in datasets become more diverse, it has been observed that mutations occurring in non-contact surface regions can also significantly impact binding affinity. Levy et al.[2] partitioned the three-dimensional structure of protein complexes into distinct regions and identified the core region as the most critical area directly involved in protein–protein interactions at the binding interface (see Fig. S2).

Building on these findings, we propose a dynamic residue selection strategy designed to capture the structural context most relevant to affinity changes while maintaining computational tractability (Fig. S3). Instead of processing the entire protein complex, our method adaptively extracts a 128-residue subgraph centered around the mutation site (denoted as $N_{res} = 128$). This subgraph serves as the input to IGMI and represents a fixed-size, mutation-focused structural neighborhood that preserves both local and semi-global geometric context. Using this subgraph ensures a stable computational footprint across complexes of different sizes and constrains the transformer attention complexity to $O(128^2)$.

Specifically, we first compute the geometric center C of the interface core region. Given the target core-region occupancy threshold G, the movement step size lr_d, and the maximum number of movement steps $N_{Maxstep}$, we initialize a virtual centroid V and define the 128 residues nearest to V as the dynamic region. The virtual centroid V moves from the mutation site toward the geometric center C in steps of size lr_d, up to $N_{Maxstep}$ iterations. The process terminates when the selected 128-residue region achieves the target coverage threshold G of the core residues or when the movement limit is reached. In this study, we set $G = 75\%$, $lr_d = 2$, and $N_{Maxstep} = 20$.

This dynamic selection mechanism ensures that the model consistently receives a structurally meaningful and size-controlled representation of each complex, enabling efficient training and stable performance across datasets.

1.2.2 Feature Extraction

For the input PDB structures, we consider multi-level features. At the residue-based, we focus on three dimensions of features: sequence-level, local structural-level, and global spatial structure-level features. At the sequence dimension, we capture the position of each residue in the primary sequence and encode the residue types using one-hot encoding, as the name may contain valuable physicochemical information. At the local structural level, we account for structural invariance, meaning the extracted features remain unchanged under translation and rotation of the protein complex. To achieve this, we construct a local coordinate system for each residue and map the heavy atoms within each residue from the global coordinate system to the local coordinate system. Taking the local coordinate mapping of residue i as an example, $x_{ia} \in \mathbb{R}^3$

denotes the coordinates of atom a in the global coordinate system. Based on the geometric relationships between the C, CA, and N atoms, we construct a Euclidean coordinate system $T_i = (R_i, O_i)$. Here, R_i represents the Euclidean transformation matrix for residue i, and O_i denotes the coordinate origin. Thus, we have:

$$R_i = \begin{bmatrix} e_{ix} & e_{iy} & e_{iz} \end{bmatrix} \in \mathbb{R}^{3\times3}, \quad O_i = x_{iC_\alpha} \tag{1}$$

$$\boldsymbol{e}_{ix} = \frac{x_{iC} - x_{iC_\alpha}}{| x_{iC} - x_{iC_\alpha} |}, \quad \boldsymbol{e}_{iy} = \frac{x_{iN} - (x_{iN} - x_{iC_\alpha})^T \cdot \boldsymbol{e}_{ix} \cdot \boldsymbol{e}_{ix}}{| x_{iN} - (x_{iN} - x_{iC_\alpha})^T \cdot \boldsymbol{e}_{ix} \cdot \boldsymbol{e}_{ix} |}, \quad \boldsymbol{e}_{iz} = \boldsymbol{e}_{ix} \times \boldsymbol{e}_{iy} \tag{2}$$

Next, we can compute the mapping of each atomic residue in the local coordinate system:

$$x_{ia} = R_i^T R_i (x_{ia} - O_i) \ , a \in \{C, N, O, C_\alpha, C_\beta, \ldots\} \tag{3}$$

Additionally, at the local structural level, we also incorporate residue types information in the form of one-hot encoding to enrich the feature set. At the global spatial structure level, we consider the Euclidean distance between residues to represent global structural information. On the atomic-based, we focus on the geometric relationship between the side chains and the backbone of the residues, simplifying this by using CB to represent the side chain group.

1.3 Model Architecture

1.3.1 Protein representation as a graph

We input pre- and post-mutated protein complexes containing N_{res} residues, indexed by i and j. The input whole protein complex is represented by the residue sequence as $\{V_i\}_{i=1\ldots N_{res}}$, the relationship between two residues is denoted by $\{e_{ij}\}_{i,j=1\ldots N_{res}}$, and the side-chain geometry conformation of each residue is characterized at the atomic-based using $\{b_i\}_{i=1\ldots N_{res}}$ as a bias term. The input to our network can be represented as a graph with bias terms, as shown in Equation 4:

$$\mathcal{G} = \left[\left(\{v_i\}_{i=1..N_{res}}, \{e_{ij}\}_{i,j=1..N_{res}} \right), \{b_i\}_{i=1\ldots N_{res}} \right] \tag{4}$$

In this study, we leverage three key perspectives for feature representation: multidimensional, multi-scale, and multi-level. These perspectives allow us to capture the full complexity of protein structures and interactions, which is crucial for predicting the effects of mutations.

- Multidimensional: Refers to the spatial dimensions in protein data representation. Specifically, the 1D sequences features capture the linear sequence relationships, 2D contact maps features represent the global spatial configuration of the protein, and 3D structures features encode the local interactions between residues (for further details, see Feature Extraction).
- Multi-scale: Focuses on the granularity of protein features, from atomic-based precision to residue-based abstraction, enabling the representation of both fine-grained and coarse-grained information.
- Multi-level: Combines features across these dimensions and granularity levels to provide hierarchical and comprehensive feature integration for downstream tasks.

Node residue features integrate local structural information derived from the heavy atoms of each residue together with residue type representations. To further enrich residue-level semantics, we incorporate contextual embeddings from the ProtTrans(Elnaggar, et al., 2021) protein language model as an additional node-wise feature source. These pretrained embeddings provide complementary biochemical and evolutionary information that is not captured by geometric descriptors alone. For residue-pair features, we employ both 1D sequences features and 2D contact maps features. The 1D sequence features encode relative positional relationships and residue-pair types along the primary sequence, whereas the 2D contact maps features store Euclidean distances between residue pairs at the global structural level. In parallel, atomic-based features represent the relative spatial geometry between each residue's side chain and backbone. Detailed formulations of these feature components are provided in the corresponding sections of the Methods.

1.3.2 Protein feature coding

The Transformer architecture has been successfully applied in the field of natural language processing, where its core function is to understand and process input text for subsequent tasks. In macromolecular encoding tasks, the Transformer is required to understand and process input structures, which is conceptually very similar to natural language processing tasks. Its potential has been demonstrated in Graphormer(Ying, et al., 2021).

1.3.2.1 3D Structures Coding

The node features $\{V_i\}_{i=1\ldots N_{res}}$ integrate three sources of information: amino-acid identity, local heavy-atom geometry, and ProtTrans-derived sequence embeddings. For each residue, the encoder takes four inputs: (i) an amino-acid index, (ii) the 3D coordinates of 14 heavy atoms, (iii) a 1024-dimensional ProtTrans embedding, and (iv) an atom-existence mask. A local coordinate frame is first constructed from the $N - CA - C$ atoms, and all heavy-atom coordinates are transformed from global to local space; nonexistent atoms are masked out. The resulting local coordinates are combined with residue identity to form a $21 \times \text{Atom\_num} \times 3$ tensor, which is flattened into an 882-dimensional geometric feature vector (crd_feat). This geometric descriptor is then concatenated with the ProtTrans embedding to produce a 1906-dimensional input, which is passed through a four-layer multilayer perceptron (Linear - ReLU - Linear - ReLU - Linear - ReLU - Linear). The output is a D_v-dimensional per-residue node embedding (N_{res}, D_v), serving as the initial node representation for downstream graph-based modeling. These node embeddings are then processed by multi-head attention. For the h-th attention head, we have:

$$A_{ij}^h = \frac{(v_i W_q^h)(v_j W_k^h)^T}{\sqrt{D_k}} \quad (5)$$

Where $v, v_j \in \mathbb{R}^{1\times D_v}$ is the encoding of node i and j, $W_q^h, W_k^h \in \mathbb{R}^{D_v \times D_k}$ are trainable parameters.

1.3.2.2 2D Contact maps Coding

The Transformer's global perception of input allows each output to focus on any location, but in protein structure coding, aligning residue nodes in three-dimensional space presents a challenge due to their multidimensional positioning and interconnections.

For this purpose, we use a simple spatial relative position coding matrix for this purpose. Specifically, for protein 3D structure, we consider a simple metric function $\phi(v_i, v_j): V \times V \to R^+$. It measures the distance scale information between v_i and v_j in the global 3D spatial structure of the protein. As nodes v_i and v_j are further apart in space the value of $\phi(v_i, v_j)$ increases, indicating that the correlation between v_i and v_j is weaker; The closer nodes v_i and v_j are in space the smaller the value of $\phi(v_i, v_j)$ becomes, indicating a stronger correlation between v_i and v_j, which is consistent with biological understanding. We use the output value as a bias term in the self-attention module. Denote by A_{ij}^h the $(i，j)$ element of the query-key product matrix in the h-header, we have：

$$A_{ij}^h = \frac{(v_i W_q^h)(v_j W_k^h)^T}{\sqrt{D_k}} - \phi(v_i, v_j) \quad (6)$$

Where $v_i, v_j \in \mathbb{R}^{1\times D_v}$ is the encoding of node i and j, $W_q^h, W_k^h \in \mathbb{R}^{D_v \times D_k}$ are trainable parameters.

1.3.2.3 1D Sequences Coding

At the 1D sequences level, for each pair of nodes, we consider the following two aspects: the distances S_{ij} between residues at the 1D sequences level, and residue pair types N_{ij}. Specifically, in terms of one-dimensional edges S_{ij}, for each node pair v_i and v_j we compute the relative sequence distance of the cuts made in a chain and encode it as a 64-dimensional vector. Since we cut the maximum value of 32, any distances that are larger on the same chain will not

be distinguished by this feature. This inductive bias de-emphasizes distances in primary sequences. Compared to the more traditional approach of encoding positions in the frequency space, this relative encoding scheme empirically allows the network to be evaluated without quality degradation on much longer sequences than it was trained on. A related structure is used in transformer-xl. For the edge type Information N_{ij} (20 residues are connected two by two, for a total of 210 connection types), we first perform two separate linear transformations to map the one-hot vectors representing the residue names into 64 dimensions, and then outer product the two. Finally, we fuse the information of these two aspects to get the 1D Sequences Coding. Specifically, we construct a function $\tau(S_{ij}, N_{ij})$: $\mathbb{R} \times \mathbb{R} \rightarrow \mathbb{R}$, which merges edge features into the attention module via bias terms. We modify the (i, j) element of A^h in Equation 7. We have:

$$A_{ij}^h = \underbrace{\frac{(v_i W_q^h)(v_j W_k^h)^T}{\sqrt{D_k}}}_{\alpha_{1ij}^h} - \underbrace{\phi(v_i, v_j)}_{\alpha_{2ij}^h} + \underbrace{\tau(S_{ij}, N_{ij})}_{\alpha_{3ij}^h} \quad (7)$$

Where we define the three terms on the right-hand side of the equation as α_{1ij}^h, α_{2ij}^h and α_{3ij}^h.

1.3.3 Antisymmetric Network

In addition to the core modules of IGMI, we apply the attention mechanism to the node and edge features using the weight matrix computed from Equation 15, which updates the residue node and edge features, as illustrated in Fig. 2b. The node, edge, and atomic features (Equation 19) are then concatenated to obtain the complex features. These features are further encoded using a fully connected feedforward neural network and a residue network to generate the final complex encoding, which is used to update the node features. This process is iterated four times, with no parameter sharing between iterations. Finally, the wild-type and mutant complexes are encoded using the IGMI architecture, yielding two encoding matrices, $u_{wt}, u_{mut} \in \mathbb{R}^{N_{res} \times 128}$. These features were used as input to the following antisymmetric network so as to predict the difference in binding affinity between the two complexes. For the affinity change of residue i, we have:

$$\Delta\Delta G_i = \left(FFN\left(u_{wti} \oplus u_{muti}\right) - FFN\left(u_{muti} \oplus u_{wti}\right)\right) W_{\Delta\Delta G} \quad (8)$$

$$FFN(p_i) = \delta\left(\delta\left(\delta\left(p_i W_1 + b_1\right) W_2 + b_2\right) W_3 + b_3\right) \quad (9)$$

Where, $\oplus$ refers to the concatenation operation, and $W_1, W_2, W_3, W_{\Delta\Delta G}, b_1, b_2, b_3$ represents the trainable parameters, $W_1 \in \mathbb{R}^{256\times128}, W_2, W_3 \in \mathbb{R}^{128\times128}, W_{\Delta\Delta G}, b_1, b_2, b_3 \in R^{128\times1}$. The ReLU function is denoted by $\delta(\cdot)$. Our prediction network consists of a fully connected feedforward network, which is applied identically to each position in both the wild-type and mutant complexes. This network comprises four linear transformations, with three ReLU activations in between. Finally, a linear transformation is applied to the encoded affinity change for residue i before and after mutation, yielding the output. The overall complex binding affinity change is then computed as:

$$\Delta\Delta G = \sum_{i=1}^{N_{res}} \Delta\Delta G_i \quad (10)$$

1.4. Evaluation Metrics and Statistical Analysis

To evaluate the performance of IGMI, we employed several widely used metrics in protein-protein interaction (PPI) and ΔΔG (ΔΔG) prediction tasks. These metrics include Mean Absolute Error (MAE), Root Mean Squared Error (RMSE), Coefficient of Determination (R^2), and Pearson correlation coefficient (Rp), each chosen to capture different aspects of model accuracy and robustness.

MAE (Mean Absolute Error) quantifies the average absolute difference between predicted and experimental ΔΔG values, providing a direct measure of prediction accuracy. The formula for MAE is given by:

$$\mathrm{MAE}=\frac{1}{n}\sum_{i=1}^{n}\left|y_i-\hat{y}_i\right| \tag{11}$$

where $\hat{y}_i$ is the predicted value, y_i is the experimental value, and n is the number of data points.

RMSE (Root Mean Squared Error) penalizes larger prediction errors more heavily, providing insights into the model’s precision. The RMSE formula is:

$$\mathrm{RMSE}=\sqrt{\frac{1}{n}\sum_{i=1}^{n}\left(y_i-\hat{y}_i\right)^2} \tag{12}$$

This metric is particularly sensitive to outliers and reflects the magnitude of the model's prediction errors.

$\boldsymbol{R^2}$ **(Coefficient of Determination)** assesses how well the predicted ΔΔG values align with the experimental data. It quantifies the proportion of variance in the experimental ΔΔG values that can be explained by the model's predictions. The R^2 formula is:

$$R^2=1-\frac{\sum_{i=1}^{n}(y_i-\hat{y}_i)^2}{\sum_{i=1}^{n}(y_i-\overline{y})^2} \tag{13}$$

where $\hat{y}_i$ are the predicted values, y_i are the experimental values, and $\overline{y}$ is the mean of the experimental values. A higher R^2 value indicates better model fit.

Rp (Pearson correlation coefficient) measures the linear correlation between the predicted and experimental ΔΔG values. The closer the value of Rp is to 1, the stronger the linear relationship. The formula for Rp is:

$$R_p=\frac{\sum_{i=1}^{n}(y_i-\overline{y})(\hat{y}_i-\overline{\hat{y}})}{\sqrt{\sum_{i=1}^{n}(y_i-\overline{y})^2\sum_{i=1}^{n}(\hat{y}_i-\overline{\hat{y}})^2}} \tag{14}$$

where $\overline{\hat{y}}$ and y_i are the predicted and experimental ΔΔG values, $\overline{\hat{y}}$ and $\overline{y}$ are their respective means. A higher Rp indicates a stronger linear relationship between predicted and experimental values. To assess the reliability of the Pearson correlation coefficient, we report the associated P-value that indicates the statistical significance of the correlation. A P-value less than 0.05 indicates a statistically significant linear relationship between the predicted and experimental ΔΔG values.

To analyze the effect of mutations on residues in different regions of the protein complex, we divided the regions to which the complex residues belonged in our analysis of the model results. According to the classification method proposed by Levy(Levy, 2010), protein complexes can be classified into five regions: Core, Rim, Support, Interior, and Surface, as shown in Fig. S2. The classification of residues primarily relies on the alteration in the relative residue accessible surface area ($rASA$) between the protein-protein complex ($rASA_c$) and the individual protein components of the complex ($rASA_m$), as depicted in Table S3. The accessible surface area of each residue was determined using the suite SASA from the Biopython library, and the relative solvent accessibility was obtained by normalizing the absolute value with that of the corresponding amino acid in a G-X-G peptide(Miller, et al., 1987).

In addition to these standard metrics, we utilized the split-by-structure cross-validation (SSCV) with 10 folds to assess the model's generalizability. The dataset was divided into ten subsets with no structural domain overlap. Each subset was used as the validation set while the remaining subsets served as the training set. The performance metrics were averaged across the ten folds to provide a robust evaluation of IGMI.

We also conducted a comprehensive interpretability analysis at both the macro and micro levels. At the macro level, we evaluated the differences in regions that the model attended to, performing t-tests on the residues the model focused on in different regions. Differences were considered significant when $p<0.05$. At the microscopic level, for each individual complex, we analyzed the top 5 residues with the highest attention weights assigned by the model. For mutation pairs, we focused on the changes induced by the mutations and examined regions within the complex where attention weights were significantly altered before and after the mutation.

We compared the performance of IGMI against several state-of-the-art methods, including GeoPPI, and TopNetTree, which represent competitive techniques in the field. Performance results were visualized using scatter plots, bar charts, and box plots to illustrate predicted versus experimental ΔΔG values. In addition, 95% confidence intervals were calculated for Rp to assess the reliability of the results.

2 Pseudocode

Algorithm 1 Dynamic Residues Selection

Algorithm 1 Dynamic Residues Selection

Input:

data: Shape as $(L,\ )$. Each element of L contains all the information characterizing each residue in that protein complex, e.g., atomic coordinates, residue name, corresponding chain, index on the residue sequence.

pos: Shape as $(L, 14, 3)$. Each element of L records information on the 3D coordinates of all heavy atoms in the residue.

core_index: Shape as $(N_{core},)$. An index of all the residues located in the core region of this protein complex was recorded.

mut_index: Shape as $(N_{mut},)$. An index of all mutated residues in this protein complex was recorded.

G: Expected CORE regional share.

lr_d: Optimising the move step of the virtual mutation point at each iteration.

$N_{Maxstep}$: Maximum number of iterations.

N_{res}: Number of residues selected.

Output:

select_data: Shape as $(N_{res},)$. Each element of N_{res} records all the characteristic information of the corresponding residue.

FUNCTION DynamicResiduesSelection (data, pos, core_index, mut_index,G , $\mathrm{lr_d}$, $\mathrm{N_{Maxstep}}$, $\mathrm{N_{res}}$):

  # Extract CA coordinates from pos

  SET pos_CA TO pos[:, 1, :]

  # Extract core and mutated CA coordinates

  SET pos_core_CA TO pos_CA[core_index]

  SET pos_mut_CA TO pos_CA[mut_index]

  # Initialize selection

  # Find the indices of the N_{res} residues closest to pos_mut_CA

  SET select_index TO indices of the $\mathrm{N_{res}}$ closest residues to pos_mut_CA based on pos_CA

```
# Calculate the geometric center of all points in pos_core_CA
SET pos_core_CA_center TO the geometric center of all points in pos_core_CA
SET rnum TO 0
# Iteratively refine selection
WHILE (LENGTH(SET(core_index) - SET(select_index)) / LENGTH(core_index)) > (1 - G) DO
  # Move the virtual mutation point towards the core center
  SET pos_mut_CA TO pos_mut_CA moved towards pos_core_CA_center by lr_d
  # Update select_index with the N_res closest residues to pos_mut_CA
  SET select_index TO indices of the N_res closest residues to pos_mut_CA based on pos_CA
  INCREMENT rnum BY 1
  IF rnum >= N_Maxstep THEN
    BREAK
  END IF
END WHILE
SET select_data TO data[select_index]
RETURN select_data
END FUNCTION
```

Algorithm 2 2D Contact Maps Coding

Algorithm 2 2D Contact Maps Coding

Input:

$\{f_i^{pos\_CB}\}$: Shape as $(N_{res}, 3)$. The coordinates of the CB atom corresponding to the residue are recorded for each element of N_{res}.

Output:

$\{d_{ij}^h\}$: Shape as (N_{res}, N_{res}, H).

FUNCTION 2DContactMapsCoding ($\{f_i^{pos\_CB}\}$):

$gamma = softplus(W)$ $gamma, W \in \mathbb{R}^H$, W is a learnable parameter with an initial value of $log(e-1)$ for each element

$d_{ij} = norm(f_i^{pos_{CB}} - f_j^{pos\_CB})$ $d_{ij} \in \mathbb{R}$, units: Å, $i, j \in \{1, \dots, N_{res}\}$

$g_{ij} = sigmoid(LinearNoBias(d_{ij}))$ $h \in \{1, \dots, H\}$, The initial value of the learnable parameter in LinearNoBias is 10

$d_{ij} = \frac{\sqrt{2}}{6} \cdot gamma \cdot d_{ij} \cdot g_{ij}$ $d_{ij} \in \mathbb{R}^H$

RETURN $\{d_{ij}^h\}$

END FUNCTION

Algorithm 3 1D Sequences Coding

Algorithm 3 1D Sequences Coding

Input:

$\{f_i^{chain}\}$: Shape as $(N_{res},)$. Each element of N_{res} corresponds to the chain to which the residue belongs.

$\{f_i^{seq}\}$: Shape as $(N_{res},)$. Each element of N_{res} corresponds to the ordinal number of the residue on the primary sequence.

$\{f_i^{res\_type}\}$: Shape as $(N_{res},)$. Each element of N_{res} corresponds to the name of the residue.

$\{f_i^{pos\_CB}\}$: Shape as $(N_{res}, 3)$. The coordinates of the CB atom corresponding to the residue are recorded for each element of N_{res}.

Output:

$\{p_{ij}^h\}$: Shape as (N_{res}, N_{res}, H).

FUNCTION 1DSequencesCoding ($\{f_i^{chain}\}, \{f_i^{seq}\}, \{f_i^{res\_type}\}$):
  # Encoding sequence, structure and type information
  $d_{ij}^{seq} = f_i^{seq} - f_j^{seq}$ $i, j \in \{1, \dots, N_{res}\}$
  IF $f_i^{chain} \neq f_j^{chain}$ **THEN**
    $d_{ij}^{seq} = 33$
  ELIF $d_{ij}^{seq} < -32$ **THEN**
    $d_{ij}^{seq} = -32$
  ELIF $d_{ij}^{seq} > 32$ **THEN**
    $d_{ij}^{seq} = 32$
  END IF
  $d_{ij}^{seq} = Embedding(\, d_{ij}^{seq} + 32)$
  $f_i^{res\_type\_OneHot} = one\_hot(f_i^{res\_type})$ $f_i^{res\_type\_OneHot} \in \mathbb{R}^{N_{res} \times 21}$
  $d_{ij}^{res\_type} = Linear\left(f_i^{res\_type\_OneHot}\right) + Linear\left(f_j^{res\_type\_OneHot}\right)$
  $d_{ij}^{seq}, d_{ij}^{res\_type} \in \mathbb{R}^{64}$
  # Output projection
  $d_{ij}^{seq} \leftarrow LayerNorm(d_{ij}^{seq})$
  $d_{ij}^{res\_type} \leftarrow LayerNorm(d_{ij}^{res\_type})$
  $p_{ij} = Linear(d_{ij}^{seq} + d_{ij}^{res\_type})$ $p_{ij} \in \mathbb{R}^H$
  RETURN $\{p_{ij}^h\}$
END FUNCTION

Algorithm 4 ProteoMAE

Algorithm 4 ProteoMAE

Input:

$\{v_{ij}^h\}$: Shape as (N_{res}, N_{res}, H). Correlation matrix between residue pairs obtained using node feature encoding.

$\{d_{ij}^h\}$: Shape as (N_{res}, N_{res}, H). Gating strongly affects coding matrix.

$\{p_{ij}^h\}$: Shape as (N_{res}, N_{res}, H). Structural fusion coding matrix.

Output:

$\{A_{ij}^h\}$: Shape as (N_{res}, N_{res}, H). Correlation matrix after modelling dependencies between multi-scale features.

FUNCTION ProteoMAE ($\{v_{ij}^h\}, \{d_{ij}^h\}, \{p_{ij}^h\}$):
  # Global node aggregation
  $v_j = \sum_i \sum_h v_{ij}^h$
  $d_j = \sum_i \sum_h d_{ij}^h$
  $p_j = \sum_i \sum_h p_{ij}^h$ $j \in \{1, \dots, N_{res}\}, h \in \{1, \dots, H\}$
  # Adaptive recalibration
  $x_m \leftarrow \{v_j\}, \{d_j\}, \{p_j\}$ $x_m \in \mathbb{R}^{N_{res}}, m \in \{1, 2, 3\}$
  $Q_m^h, K_m^h, V_m^h = LinearNoBias(x_m)$ $Q_m^h, K_m^h, V_m^h \in \mathbb{R}^c$
  $a_{mn}^h = softmax_n\left(\frac{1}{\sqrt{c}} {Q_m^h}^T K_n^h\right)$
  $O_m^h = \sum_n a_{mn}^h V_n^h$
  $O_m = Linear\left(concat_h(O_m^h)\right)$
  $a = softplus(O_0)$
  $b = softplus(O_1)$
  $c = sigmoid(O_2)$
  # Output projection
  $A_{ij}^h = softmax_j\left(a v_{ij}^h - c d_{ij}^h + b p_{ij}^h\right)$
  RETURN $\{A_{ij}^h\}$
END FUNCTION

Algorithm 5 BackSideAttention

Algorithm 5 BackSideAttention

Input:

$\{R_i\}$: Shape as $(N_{res}, 3, 3)$. Each element in N_{res} records the Euclidean transformation matrix of the corresponding residue.

$\{A_{ij}^h\}$: Shape as (N_{res}, N_{res}, H). Correlation matrix after modelling the dependencies between multi-scale features at the residue level.

$\{f_i^{pos\_CB}\}$: Shape as $(N_{res}, 3)$. The coordinates of the CB atom corresponding to the residue are recorded for each element of N_{res}.

$\{f_i^{pos\_CA}\}$: Shape as $(N_{res}, 3)$. The coordinates of the CA atom corresponding to the residue are recorded for each element of N_{res}.

Output:

$\{S_i\}$: Shape as $(N_{res}, 7H)$. Encoding of side-chain geometry features at the atomic level after establishing informative interactions with the residue level.

FUNCTION BackSideAttention ($\{R_i\}, \{A_{ij}^h\}, \{f_i^{pos\_CB}\}, \{f_i^{pos\_CA}\}$):
  # Mapping to multidimensional space
  $f_i^{h^{pos\_CB}} = \sum_j A_{ij}^h f_j^{pos\_CB}$
  # Euclidean transformation
  $f_i^{h^{\text{pos\_CB}}} = R_i^T \left(f_i^{h^{\text{pos\_CB}}} - f_i^{\text{pos\_CA}} \right)$
  # Direction, distance and position
  $\xi_i^h = f_i^{h^{pos\_CB}}$ $\xi_i^h \in \mathbb{R}^3$
  $\zeta_i^h = norm\left(f_i^{h^{pos\_CB}} \right)$ $\zeta_i^h \in \mathbb{R}$
  $\psi_i^h = \frac{f_i^{h^{pos\_CB}}}{norm\left(f_i^{h^{pos\_CB}} \right)}$ $\psi_i^h \in \mathbb{R}^3$
  $S_i = concat_{\chi \in \{\xi, \zeta, \psi\}} \left(concat_h \left(\chi_i^h \right) \right)$ $S_i \in \mathbb{R}^{7H}$
  RETURN $\{S_i\}$
END FUNCTION

Algorithm 6 Greedy Balanced Fold Partitioning

Algorithm 6 Greedy Balanced Fold Partitioning

Input:

$\{L_c\}$: Shape as $(M,)$. Each element stores the index list of data points belonging to cluster c.

$\{N\}$: Integer. Number of folds.

$\{\varepsilon\}$: Integer. Capacity slack, $\varepsilon \in \mathbb{N}(0,10)$.

Output:

$\{F_k\}$: Shape as $(N,)$. Each element is the index list assigned to fold k.

FUNCTION GreedyBalancedFoldPartitioning ($\{L_c\}, N, \varepsilon$):
  # Cluster sizes and ordering
  $s_c = len(L\_c)$ # Size of cluster c
  C' =Argsort(c) by s_c in descending **order**

  # Initialization
  $D_{size} = \sum_c s_c$ # Total number of data points
  $AvgN = \frac{D_{size}}{N}$ # Average fold size
  $folds \ = \ dict()$ # stores $\{F_k\}$
  $used\_cluster = []$ # Track assigned clusters
  $fold\_id = 0$

  # Greedy partitioning
  FOR $i \in C'$ **DO**
    IF len(folds) == N **THEN**
      BREAK

```
        ELSE
            SET F_fold_id ← L_i
        END IF
        threshold = AvgN + ε

        # Fill current fold with smaller clusters
        FOR j ∈ C′ DO
            IF j ∈ used_cluster THEN CONTINUE
            IF len(L_j) ≥ len(L_i) THEN CONTINUE
            IF len(F_fold_id) + len(L_j) < threshold THEN
                SET F_fold_id ← F_fold_id ∪ L_j
                APPEND j TO used_cluster
            ELSE
                INCREMENT fold_id BY 1
            END IF
    END FOR

    # Assign remaining clusters to the last fold
    FOR j in C′ DO
        IF j not in used_cluster THEN
            SET F_{N−1} ← F_{N−1}L_j
        END IF
    END FOR
    RETURN {F_k}
END FUNCTION
```

3 Baseline Reproduction Details

3.1 Hardware and Software Environment

All baseline models were reproduced under the same computational environment used for IGMI to ensure fully comparable results. The hardware and software specifications are listed in Table S7. This unified setup eliminates variability caused by hardware differences or framework discrepancies.

3.2 Reproduction Strategy for Baseline Methods

3.2.1 Data-driven Baselines

We reproduced GeoPPI, TopGBT, TopNetTree, MpbPPI, DGCddg and MutaBind2 using their official public implementations. For each method:

1. The same dataset partitions as IGMI were used.
2. Features were processed according to each model's original requirements.
3. Training/evaluation was conducted under identical hardware versions.
4. All methods were evaluated using the same performance metrics (Rp, RMSE).

Where hyperparameter defaults were not fully documented, we used the default settings provided in the official codebases to ensure faithful reproduction.

3.2.2 Energy-based Baselines (FoldX, BeAtMuSic)

FoldX and BeAtMuSic are not learning-based models and do not support retraining. They generate ΔΔG predictions via deterministic energetic scoring functions. Therefore:

1. We executed the official FoldX 5.0 and BeAtMuSic Web/Local package on the same preprocessed mutant structures used by IGMI.
2. No parameter tuning or stochastic operations were applied.

3. Their predictions depend solely on their internal scoring potentials, making forward execution on standardized inputs the correct and only reproducible approach.

This strategy follows common practice in ΔΔG prediction studies.

3.4 Baseline Reproduction Code and Resources

All reproduced baseline model repositories are available in our Google Drive collection (https://drive.google.com/drive/folders/1CYxd-utnrIKLUtyZ-EfTCUX6WbGRRn8E?usp=sharing). FoldX can be obtained from its official website (https://foldxsuite.crg.eu/), and BeAtMuSic is accessible through its official web server (http://babylone.ulb.ac.be/beatmusic/).

4 Supplementary Tables

Table S1. Comparison of Pearson correlation coefficient of various methods for the single-point mutations in ten-fold random-split cross-validation (RSCV) on the S1131, S4169, S4191 and S8338 datasets. †: Results were obtained based on the GeoPPI study by Liu et al(Liu, et al., 2021). ‡ : Results were obtained based on the released data.

Method	S1131	S4169	S8338
IGMI	**0.87**	**0.80**	**0.85**
GeoPPI†	0.85	0.78	0.85
TopGBT†	0.78	0.76	0.82
TopNetTree†	0.76	0.77	0.81
mCSM-PPI2†	-	0.76	0.82
MutaBind2†	-	0.74	0.81
BindProfX†	0.74	-	-
Profile-score + FoldX†	0.74	-	-
Profile-score†	0.68	-	-
SAAMBE†	0.62	-	-
FoldX†	0.46	0.27	0.44
BeAtMuSic†	0.27	-	-
Dcomplex†	0.06	-	-
MpbPPI‡(Yang et al.(Yue, et al., 2023))	0.87	0.80	-
DGCddg‡(Jiang et al.(Jiang, et al., 2023))	0.85	-	-

Table S2. Comparison of Pearson correlation coefficients for multiple methods on multi-point mutations under 10-fold RSCV on the M1707 dataset. †: Results were obtained based on the GeoPPI study by Liu et al.

Method	M1707
**IGMI**	**0.94**
GeoPPI†	0.89

MutaBind2†	0.87
FoldX†	0.49

Table S3. Guidelines for Residue Region Classification. $\Delta rASA = \Delta rASA_m - \Delta rASA_c$

Region	$\Delta rASA$	$\Delta rASA_c$	$\Delta rASA_m$
Interior	0	<25%	-
Surface	0	>25%	-
Rim	>0	>25%	-
Support	>0	-	<25%
Core	>0	<25%	>25%

Table S4. The S1131 dataset is partitioned according to the SSCV protocol.

fold	train_complexes	test_complexes
0	1A22_AB,1A4Y_AB,1ACB_EI,1AK4_AD,1B2S_AD,1B2U_AD,1B3S_AD,1BRS_AD,1CSE_EI,1CSO_EI,1CT0_EI,1CT2_EI,1CT4_EI,1E96_AB,1EAW_AB,1EFN_AB,1EMV_AB,1F47_AB,1FC2_CD,1FCC_AC,1FFW_AB,1GC1_GC,1GL0_EI,1GL1_AI,1H9D_AB,1HE8_AB,1IAR_AB,1JCK_AB,1JTG_AB,1KAC_AB,1KTZ_AB,1LFD_AB,1M9E_AD,1MAH_AF,1P69_AB,1P6A_AB,1PPF_EI,1R0R_EI,1S0W_AC,1S1Q_AB,1SBB_AB,1SBN_EI,1SGD_EI,1SGE_EI,1SGN_EI,1SGP_EI,1SGQ_EI,1SGY_EI,1SMF_EI,1TM1_EI,1TM3_EI,1TM4_EI,1TM5_EI,1TM7_EI,1TMG_EI,1TO1_EI,1UUZ_AD,1X1X_AD,1XD3_AB,1Y1K_EI,1Y33_EI,1Y34_EI,1Y3B_EI,1Z7X_WX,2A9K_AB,2AJF_AE,2B0Z_AB,2B10_AB,2B11_AB,2B12_AB,2B42_AB,2BTF_AP,2FTL_EI,2G2U_AB,2G2W_AB,2GOX_AB,2HLE_AB,2HRK_AB,2I26_NL,2J0T_AD,2J1K_CT,2NU0_EI,2NU1_EI,2NU2_EI,2NU4_EI,2O3B_AB,2OOB_AB,2PCB_AB,2PCC_AB,2SGP_EI,2SGQ_EI,2SIC_EI,2VLN_AB,2VLO_AB,2VLQ_AB,2WPT_AB,3BK3_AC,3BP8_AC,3BTD_EI,3BTE_EI,3BTF_EI,3BTG_EI,3BTH_EI,3BTM_EI,3BTQ_EI,3BTT_EI,3BTW_EI,3D5R_AC,3D5S_AC,4CPA_AI	3SGB_EI

1	1A22_AB,1A4Y_AB,1ACB_EI,1AK4_AD,1B2S_AD,1B2U_AD,1B3S_AD,1BRS_AD,1CSE_EI,1CSO_EI,1CT0_EI,1CT2_EI,1CT4_EI,1E96_AB,1EAW_AB,1EFN_AB,1EMV_AB,1F47_AB,1FC2_CD,1FCC_AC,1FFW_AB,1GC1_GC,1GL0_EI,1GL1_AI,1H9D_AB,1HE8_AB,1IAR_AB,1JCK_AB,1JTG_AB,1KAC_AB,1KTZ_AB,1LFD_AB,1M9E_AD,1MAH_AF,1P69_AB,1P6A_AB,1R0R_EI,1S0W_AC,1S1Q_AB,1SBB_AB,1SBN_EI,1SGD_EI,1SGE_EI,1SGN_EI,1SGP_EI,1SGQ_EI,1SGY_EI,1SMF_EI,1TM1_EI,1TM3_EI,1TM4_EI,1TM5_EI,1TM7_EI,1TMG_EI,1TO1_EI,1UUZ_AD,1X1X_AD,1XD3_AB,1Y1K_EI,1Y33_EI,1Y34_EI,1Y3B_EI,1Z7X_WX,2A9K_AB,2AJF_AE,2B0Z_AB,2B10_AB,2B11_AB,2B12_AB,2B42_AB,2BTF_AP,2FTL_EI,2G2U_AB,2G2W_AB,2GOX_AB,2HLE_AB,2HRK_AB,2I26_NL,2J0T_AD,2J1K_CT,2NU0_EI,2NU1_EI,2NU2_EI,2NU4_EI,2O3B_AB,2OOB_AB,2PCB_AB,2PCC_AB,2SGP_EI,2SGQ_EI,2SIC_EI,2VLN_AB,2VLO_AB,2VLQ_AB,2WPT_AB,3BK3_AC,3BP8_AC,3BTD_EI,3BTE_EI,3BTF_EI,3BTG_EI,3BTH_EI,3BTM_EI,3BTQ_EI,3BTT_EI,3BTW_EI,3D5R_AC,3D5S_AC,3SGB_EI,4CPA_AI	1PPF_EI
2	1A22_AB,1A4Y_AB,1ACB_EI,1AK4_AD,1B2S_AD,1B2U_AD,1B3S_AD,1BRS_AD,1CSE_EI,1CSO_EI,1CT0_EI,1CT2_EI,1CT4_EI,1E96_AB,1EAW_AB,1EFN_AB,1EMV_AB,1F47_AB,1FC2_CD,1FCC_AC,1FFW_AB,1GC1_GC,1GL0_EI,1GL1_AI,1H9D_AB,1HE8_AB,1IAR_AB,1JCK_AB,1JTG_AB,1KAC_AB,1KTZ_AB,1LFD_AB,1M9E_AD,1MAH_AF,1P69_AB,1P6A_AB,1PPF_EI,1S0W_AC,1S1Q_AB,1SBB_AB,1SBN_EI,1SGD_EI,1SGE_EI,1SGN_EI,1SGP_EI,1SGQ_EI,1SGY_EI,1SMF_EI,1TM1_EI,1TM3_EI,1TM4_EI,1TM5_EI,1TM7_EI,1TMG_EI,1TO1_EI,1UUZ_AD,1X1X_AD,1XD3_AB,1Y1K_EI,1Y33_EI,1Y	1R0R_EI

	34_EI,1Y3B_EI,1Z7X_WX,2A9K_AB,2AJF_AE,2B0Z_AB,2B10_AB,2B11_AB,2B12_AB,2B42_AB,2BTF_AP,2FTL_EI,2G2U_AB,2G2W_AB,2GOX_AB,2HLE_AB,2HRK_AB,2I26_NL,2J0T_AD,2J1K_CT,2NU0_EI,2NU1_EI,2NU2_EI,2NU4_EI,2O3B_AB,2OOB_AB,2PCB_AB,2PCC_AB,2SGP_EI,2SGQ_EI,2SIC_EI,2VLN_AB,2VLO_AB,2VLQ_AB,2WPT_AB,3BK3_AC,3BP8_AC,3BTD_EI,3BTE_EI,3BTF_EI,3BTG_EI,3BTH_EI,3BTM_EI,3BTQ_EI,3BTT_EI,3BTW_EI,3D5R_AC,3D5S_AC,3SGB_EI,4CPA_AI	
3	1A4Y_AB,1ACB_EI,1B2S_AD,1B2U_AD,1B3S_AD,1BRS_AD,1CSE_EI,1CSO_EI,1CT0_EI,1CT2_EI,1CT4_EI,1E96_AB,1EAW_AB,1EFN_AB,1EMV_AB,1F47_AB,1FC2_CD,1FCC_AC,1FFW_AB,1GL0_EI,1GL1_AI,1H9D_AB,1HE8_AB,1IAR_AB,1JCK_AB,1JTG_AB,1KAC_AB,1KTZ_AB,1LFD_AB,1M9E_AD,1MAH_AF,1P69_AB,1P6A_AB,1PPF_EI,1R0R_EI,1S0W_AC,1S1Q_AB,1SBB_AB,1SBN_EI,1SGD_EI,1SGE_EI,1SGN_EI,1SGP_EI,1SGQ_EI,1SGY_EI,1SMF_EI,1TM1_EI,1TM3_EI,1TM4_EI,1TM5_EI,1TM7_EI,1TMG_EI,1TO1_EI,1UUZ_AD,1X1X_AD,1XD3_AB,1Y1K_EI,1Y33_EI,1Y34_EI,1Y3B_EI,2A9K_AB,2AJF_AE,2B0Z_AB,2B10_AB,2B11_AB,2B12_AB,2B42_AB,2BTF_AP,2FTL_EI,2G2U_AB,2G2W_AB,2GOX_AB,2HLE_AB,2HRK_AB,2I26_NL,2J1K_CT,2NU0_EI,2NU1_EI,2NU2_EI,2NU4_EI,2O3B_AB,2OOB_AB,2PCB_AB,2PCC_AB,2SGP_EI,2SGQ_EI,2SIC_EI,2VLN_AB,2VLO_AB,2VLQ_AB,2WPT_AB,3BK3_AC,3BP8_AC,3BTD_EI,3BTE_EI,3BTF_EI,3BTG_EI,3BTH_EI,3BTM_EI,3BTQ_EI,3BTT_EI,3BTW_EI,3D5R_AC,3D5S_AC,3SGB_EI,4CPA_AI	1A22_AB,1AK4_AD,1GC1_GC,1Z7X_WX,2J0T_AD
4	1A22_AB,1A4Y_AB,1ACB_EI,1AK4_AD,1B2S_AD,1B2U_AD,1B3S_AD,1BRS_AD,1CSE_EI,1CSO_EI,1CT0_EI,1CT2_EI,1	1EAW_AB,1F47_AB,1FFW_AB,1JTG_AB,1LFD_AB,1XD3_AB,2O3B_AB,2SIC_EI,3BK3_AC,4CPA_AI

	CT4_EI,1E96_AB,1EFN_AB,1EMV_AB,1FC2_CD,1FCC_AC,1GC1_GC,1GL0_EI,1GL1_AI,1H9D_AB,1HE8_AB,1IAR_AB,1JCK_AB,1KAC_AB,1KTZ_AB,1M9E_AD,1MAH_AF,1P69_AB,1P6A_AB,1PPF_EI,1R0R_EI,1S0W_AC,1S1Q_AB,1SBB_AB,1SBN_EI,1SGD_EI,1SGE_EI,1SGN_EI,1SGP_EI,1SGQ_EI,1SGY_EI,1SMF_EI,1TM1_EI,1TM3_EI,1TM4_EI,1TM5_EI,1TM7_EI,1TMG_EI,1TO1_EI,1UUZ_AD,1X1X_AD,1Y1K_EI,1Y33_EI,1Y34_EI,1Y3B_EI,1Z7X_WX,2A9K_AB,2AJF_AE,2B0Z_AB,2B10_AB,2B11_AB,2B12_AB,2B42_AB,2BTF_AP,2FTL_EI,2G2U_AB,2G2W_AB,2GOX_AB,2HLE_AB,2HRK_AB,2I26_NL,2J0T_AD,2J1K_CT,2NU0_EI,2NU1_EI,2NU2_EI,2NU4_EI,2OOB_AB,2PCB_AB,2PCC_AB,2SGP_EI,2SGQ_EI,2VLN_AB,2VLO_AB,2VLQ_AB,2WPT_AB,3BP8_AC,3BTD_EI,3BTE_EI,3BTF_EI,3BTG_EI,3BTH_EI,3BTM_EI,3BTQ_EI,3BTT_EI,3BTW_EI,3D5R_AC,3D5S_AC,3SGB_EI	
5	1A22_AB,1A4Y_AB,1AK4_AD,1BRS_AD,1CSO_EI,1CT0_EI,1CT2_EI,1CT4_EI,1EAW_AB,1F47_AB,1FCC_AC,1FFW_AB,1GC1_GC,1GL0_EI,1GL1_AI,1HE8_AB,1IAR_AB,1JTG_AB,1KTZ_AB,1LFD_AB,1M9E_AD,1P69_AB,1P6A_AB,1PPF_EI,1R0R_EI,1S0W_AC,1SBN_EI,1SGD_EI,1SGE_EI,1SGN_EI,1SGP_EI,1SGQ_EI,1SGY_EI,1TM1_EI,1TM3_EI,1TM4_EI,1TM5_EI,1TM7_EI,1TMG_EI,1TO1_EI,1X1X_AD,1XD3_AB,1Y1K_EI,1Y33_EI,1Y34_EI,1Y3B_EI,1Z7X_WX,2A9K_AB,2B0Z_AB,2B10_AB,2B11_AB,2B12_AB,2FTL_EI,2G2U_AB,2G2W_AB,2I26_NL,2J0T_AD,2NU0_EI,2NU1_EI,2NU2_EI,2NU4_EI,2O3B_AB,2OOB_AB,2SGP_EI,2SGQ_EI,2SIC_EI,2VLN_AB,2VLO_AB,2VLQ_AB,2WPT_AB,3BK3_AC,3BTD_EI,3BTE_EI,3BTF_EI,3BTG_EI,3BTH_EI,3BTM_EI,	1ACB_EI,1B2S_AD,1B2U_AD,1B3S_AD,1CSE_EI,1E96_AB,1EFN_AB,1EMV_AB,1FC2_CD,1H9D_AB,1JCK_AB,1KAC_AB,1MAH_AF,1S1Q_AB,1SBB_AB,1SMF_EI,1UUZ_AD,2AJF_AE,2B42_AB,2BTF_AP,2GOX_AB,2HLE_AB,2HRK_AB,2J1K_CT,2PCB_AB,2PCC_AB,3BP8_AC,3D5R_AC,3D5S_AC

	3BTQ_EI,3BTT_EI,3BTW_EI,3SGB_EI,4CPA_AI	
6	1A22_AB,1A4Y_AB,1ACB_EI,1AK4_AD,1B2S_AD,1B2U_AD,1B3S_AD,1CSE_EI,1E96_AB,1EAW_AB,1EFN_AB,1EMV_AB,1F47_AB,1FC2_CD,1FCC_AC,1FFW_AB,1GC1_GC,1H9D_AB,1IAR_AB,1JCK_AB,1JTG_AB,1KAC_AB,1KTZ_AB,1LFD_AB,1MAH_AF,1PPF_EI,1R0R_EI,1S1Q_AB,1SBB_AB,1SMF_EI,1TM1_EI,1UUZ_AD,1XD3_AB,1Z7X_WX,2AJF_AE,2B10_AB,2B42_AB,2BTF_AP,2FTL_EI,2G2U_AB,2GOX_AB,2HLE_AB,2HRK_AB,2J0T_AD,2J1K_CT,2O3B_AB,2PCB_AB,2PCC_AB,2SIC_EI,2WPT_AB,3BK3_AC,3BP8_AC,3D5R_AC,3D5S_AC,3SGB_EI,4CPA_AI	1BRS_AD,1CSO_EI,1CT0_EI,1CT2_EI,1CT4_EI,1GL0_EI,1GL1_AI,1HE8_AB,1M9E_AD,1P69_AB,1P6A_AB,1S0W_AC,1SBN_EI,1SGD_EI,1SGE_EI,1SGN_EI,1SGP_EI,1SGQ_EI,1SGY_EI,1TM3_EI,1TM4_EI,1TM5_EI,1TM7_EI,1TMG_EI,1TO1_EI,1X1X_AD,1Y1K_EI,1Y33_EI,1Y34_EI,1Y3B_EI,2A9K_AB,2B0Z_AB,2B11_AB,2B12_AB,2G2W_AB,2I26_NL,2NU0_EI,2NU1_EI,2NU2_EI,2NU4_EI,2OOB_AB,2SGP_EI,2SGQ_EI,2VLN_AB,2VLO_AB,2VLQ_AB,3BTD_EI,3BTE_EI,3BTF_EI,3BTG_EI,3BTH_EI,3BTM_EI,3BTQ_EI,3BTT_EI,3BTW_EI
7	1A22_AB,1ACB_EI,1AK4_AD,1B2S_AD,1B2U_AD,1B3S_AD,1BRS_AD,1CSE_EI,1CSO_EI,1CT0_EI,1CT2_EI,1CT4_EI,1E96_AB,1EAW_AB,1EFN_AB,1EMV_AB,1F47_AB,1FC2_CD,1FCC_AC,1FFW_AB,1GC1_GC,1GL0_EI,1GL1_AI,1H9D_AB,1HE8_AB,1IAR_AB,1JCK_AB,1JTG_AB,1KAC_AB,1KTZ_AB,1LFD_AB,1M9E_AD,1MAH_AF,1P69_AB,1P6A_AB,1PPF_EI,1R0R_EI,1S0W_AC,1S1Q_AB,1SBB_AB,1SBN_EI,1SGD_EI,1SGE_EI,1SGN_EI,1SGP_EI,1SGQ_EI,1SGY_EI,1SMF_EI,1TM1_EI,1TM3_EI,1TM4_EI,1TM5_EI,1TM7_EI,1TMG_EI,1TO1_EI,1UUZ_AD,1X1X_AD,1XD3_AB,1Y1K_EI,1Y33_EI,1Y34_EI,1Y3B_EI,1Z7X_WX,2A9K_AB,2AJF_AE,2B0Z_AB,2B10_AB,2B11_AB,2B12_AB,2B42_AB,2BTF_AP,2FTL_EI,2G2U_AB,2G2W_AB,2GOX_AB,2HLE_AB,2HRK_AB,2I26_NL,2J0T_AD,2J1K_CT,2NU0_EI,2NU1_EI,2NU2_EI,2NU4_EI,2O3B_AB,2OOB_AB,2PCB_AB,2PCC_AB,2SGP_EI,2SGQ_EI,2SIC_EI,2VLN_AB,2VLO_AB,2VLQ_AB,2WPT_AB,3BK3_AC,3BP8_AC,3BTD_EI,3BTE_EI,3BTF_EI,3BTG_EI,3BTH_EI,3BTM_EI,3BTQ_EI,3	1A4Y_AB

	BTT_EI,3BTW_EI,3D5R_AC,3D5S_AC,3SGB_EI,4CPA_AI	
8	1A22_AB,1A4Y_AB,1ACB_EI,1AK4_AD,1B2S_AD,1B2U_AD,1B3S_AD,1BRS_AD,1CSE_EI,1CSO_EI,1CT0_EI,1CT2_EI,1CT4_EI,1E96_AB,1EAW_AB,1EFN_AB,1EMV_AB,1F47_AB,1FC2_CD,1FCC_AC,1FFW_AB,1GC1_GC,1GL0_EI,1GL1_AI,1H9D_AB,1HE8_AB,1JCK_AB,1JTG_AB,1KAC_AB,1KTZ_AB,1LFD_AB,1M9E_AD,1MAH_AF,1P69_AB,1P6A_AB,1PPF_EI,1R0R_EI,1S0W_AC,1S1Q_AB,1SBB_AB,1SBN_EI,1SGD_EI,1SGE_EI,1SGN_EI,1SGP_EI,1SGQ_EI,1SGY_EI,1SMF_EI,1TM1_EI,1TM3_EI,1TM4_EI,1TM5_EI,1TM7_EI,1TMG_EI,1TO1_EI,1UUZ_AD,1X1X_AD,1XD3_AB,1Y1K_EI,1Y33_EI,1Y34_EI,1Y3B_EI,1Z7X_WX,2A9K_AB,2AJF_AE,2B0Z_AB,2B10_AB,2B11_AB,2B12_AB,2B42_AB,2BTF_AP,2FTL_EI,2G2U_AB,2G2W_AB,2GOX_AB,2HLE_AB,2HRK_AB,2I26_NL,2J0T_AD,2J1K_CT,2NU0_EI,2NU1_EI,2NU2_EI,2NU4_EI,2O3B_AB,2OOB_AB,2PCB_AB,2PCC_AB,2SGP_EI,2SGQ_EI,2SIC_EI,2VLN_AB,2VLO_AB,2VLQ_AB,2WPT_AB,3BK3_AC,3BP8_AC,3BTD_EI,3BTE_EI,3BTF_EI,3BTG_EI,3BTH_EI,3BTM_EI,3BTQ_EI,3BTT_EI,3BTW_EI,3D5R_AC,3D5S_AC,3SGB_EI,4CPA_AI	1IAR_AB
9	1A22_AB,1A4Y_AB,1ACB_EI,1AK4_AD,1B2S_AD,1B2U_AD,1B3S_AD,1BRS_AD,1CSE_EI,1CSO_EI,1CT0_EI,1CT2_EI,1CT4_EI,1E96_AB,1EAW_AB,1EFN_AB,1EMV_AB,1F47_AB,1FC2_CD,1FFW_AB,1GC1_GC,1GL0_EI,1GL1_AI,1H9D_AB,1HE8_AB,1IAR_AB,1JCK_AB,1JTG_AB,1KAC_AB,1LFD_AB,1M9E_AD,1MAH_AF,1P69_AB,1P6A_AB,1PPF_EI,1R0R_EI,1S0W_AC,1S1Q_AB,1SBB_AB,1SBN_EI,1SGD_EI,1SGE_EI,1SGN_EI,1SGP_EI,1SGQ_EI,1SGY_EI,1SMF_EI,1TM3_EI,1TM4_EI,1TM5_EI,1TM7_EI,1TMG_EI,1T	1FCC_AC,1KTZ_AB,1TM1_EI,2B10_AB,2FTL_EI,2G2U_AB,2WPT_AB

	O1_EI,1UUZ_AD,1X1X_AD,1XD3_AB,1Y1K_EI,1Y33_EI,1Y34_EI,1Y3B_EI,1Z7X_WX,2A9K_AB,2AJF_AE,2B0Z_AB,2B11_AB,2B12_AB,2B42_AB,2BTF_AP,2G2W_AB,2GOX_AB,2HLE_AB,2HRK_AB,2I26_NL,2J0T_AD,2J1K_CT,2NU0_EI,2NU1_EI,2NU2_EI,2NU4_EI,2O3B_AB,2OOB_AB,2PCB_AB,2PCC_AB,2SGP_EI,2SGQ_EI,2SIC_EI,2VLN_AB,2VLO_AB,2VLQ_AB,3BK3_AC,3BP8_AC,3BTD_EI,3BTE_EI,3BTF_EI,3BTG_EI,3BTH_EI,3BTM_EI,3BTQ_EI,3BTT_EI,3BTW_EI,3D5R_AC,3D5S_AC,3SGB_EI,4CPA_AI	

Table S5. The S4169 dataset is partitioned according to the SSCV protocol.

fold	train_complexes	test_complexes
0	1A22_AB,1A4Y_AB,1ACB_EI,1AHW_ABC,1AK4_AD,1AO7_ABCDE,1B2S_AD,1B2U_AD,1B3S_AD,1B41_AB,1BD2_ABCDE,1BJ1_HLVW,1BP3_AB,1BRS_AD,1C1Y_AB,1C4Z_ABCD,1CBW_FGHI,1CHO_EFGI,1CSE_EI,1CSO_EI,1CT0_EI,1CT2_EI,1CT4_EI,1CZ8_HLVW,1DAN_HLUT,1DQJ_ABC,1DVF_ABCD,1E50_AB,1E96_AB,1EAW_AB,1EFN_AB,1EMV_AB,1F47_AB,1F5R_AI,1FC2_CD,1FCC_AC,1FFW_AB,1FR2_AB,1FSS_AB,1FY8_EI,1GC1_GC,1GCQ_ABC,1GL0_EI,1GL1_AI,1GRN_AB,1GUA_AB,1H9D_AB,1HE8_AB,1IAR_AB,1JCK_AB,1JRH_LHI,1JTD_AB,1K8R_AB,1KAC_AB,1KBH_AB,1KIP_ABC,1KIQ_ABC,1KIR_ABC,1KNE_AP,1KTZ_AB,1LFD_AB,1LP9_ABCEF,1M9E_AD,1MAH_AF,1MHP_HLA,1MI5_ABCDE,1MLC_ABE,1N8O_ABCE,1N8Z_ABC,1NCA_NLH,1NMB_NLH,1OGA_ABCDE,1P69_AB,1P6A_AB,1PPF_EI,1QSE_ABCDE,1R0R_EI,1REW_ABC,1S0W_AC,1S1Q_AB,1SBB_AB,1SBN_EI,1SGD_EI,1SGE_EI,1SGN_EI,1SGP_EI,1SGQ_EI,1SGY_EI,1SIB_EI,1SMF_EI,1TM1_EI,1TM3_EI,1TM4_EI,1TM5_EI,1TM7_EI,1T	1JTG_AB,3BT1_AU,3S9D_AB

	MG_EI,1TO1_EI,1U7F_BAC,1UUZ_AD,1VFB_ABC,1WQJ_IB,1X1W_AD,1X1X_AD,1XD3_AB,1XGP_ABC,1XGQ_ABC,1XGR_ABC,1XGT_ABC,1XGU_ABC,1Y1K_EI,1Y33_EI,1Y34_EI,1Y3B_EI,1Y3C_EI,1Y3D_EI,1Y48_EI,1YCS_AB,1YQV_HLY,1YY9_CDA,1Z7X_WX,2A9K_AB,2AJF_AE,2AK4_ABCDE,2AW2_AB,2B0U_ABC,2B0Z_AB,2B10_AB,2B11_AB,2B12_AB,2B2X_HLA,2B42_AB,2BDN_HLA,2BNQ_ABCDE,2BNR_ABCDE,2BTF_AP,2C0L_AB,2C5D_AC,2DSQ_IG,2DVW_AB,2E7L_EQAD,2FTL_EI,2G2U_AB,2G2W_AB,2GOX_AB,2GYK_AB,2HLE_AB,2HRK_AB,2I26_NL,2J0T_AD,2J12_AB,2J1K_CT,2J8U_ABCEF,2JCC_ABCEF,2JEL_LHP,2KSO_AB,2NU0_EI,2NU1_EI,2NU2_EI,2NU4_EI,2NYY_DCA,2NZ9_DCA,2O3B_AB,2OI9_AQBC,2OOB_AB,2P5E_ABCDE,2PCB_AB,2PCC_AB,2REX_AB,2SGP_EI,2SGQ_EI,2SIC_EI,2UWE_ABCEF,2VIR_ABC,2VIS_ABC,2VLN_AB,2VLO_AB,2VLP_AB,2VLQ_AB,2VLR_ABCDE,2WPT_AB,3AAA_ABC,3B4V_ABC,3BK3_AC,3BN9_BCD,3BP8_AC,3BTD_EI,3BTE_EI,3BTF_EI,3BTG_EI,3BTH_EI,3BTM_EI,3BTQ_EI,3BTT_EI,3BTW_EI,3BX1_AC,3C60_CDAB,3D3V_ABCDE,3D5R_AC,3D5S_AC,3EG5_AB,3EQS_AB,3EQY_AC,3F1S_AB,3G6D_LHA,3H9S_ABCDE,3HFM_HLY,3HH2_ABC,3KBH_AE,3KUD_AB,3L5X_AHL,3LB6_AC,3LNZ_AB,3LZF_ABHL,3M62_AB,3M63_AB,3MZG_AB,3MZW_AB,3N06_AB,3N0P_AB,3N4I_AB,3N85_ALH,3NCB_AB,3NCC_AB,3NGB_HLG,3NPS_ABC,3NVN_BA,3NVQ_BA,3PWP_ABCDE,3Q3J_AB,3Q8D_AE,3QDG_ABCDE,3QDJ_ABCDE,3QFJ_ABCDE,3QHY_AB,3QIB_ABPCD,3R9A_ACB,3RF3_AC,3SE3_BA,3SE4_BA,3SE4_BC,3SE8_HLG,3SE9_HLG,3SEK_BC,3SF4_AD,3SGB_EI,3SZK_ABC,3TGK_EI,3U82_AB,3UIG_AP,3UII_AP,3VR	

	6_ABCDEFGH,3W2D_AHL,3WWN_AB,4B0M_ABM,4BFI_AB,4CPA_AI,4CVW_AC,4E6K_ABG,4EKD_AB,4FTV_ABCDE,4FZA_AB,4G0N_AB,4G2V_AB,4GNK_AB,4GXU_ABCDEFMN,4HFK_ABD,4HRN_AD,4HSA_ABC,4I77_HLZ,4J2L_ACD,4JEU_AB,4JFD_ABCDE,4JFE_ABCDE,4JFF_ABCDE,4JGH_ABCD,4JPK_HLA,4K71_ABC,4KRL_AB,4KRO_AB,4KRP_AB,4L0P_AB,4L3E_ABCDE,4LRX_ABCD,4MNQ_ABCDE,4MYW_AB,4N8V_GABC,4NKQ_CAB,4NM8_ABCDEFHL,4NZW_AB,4O27_AB,4OFY_AD,4OZG_ABJGH,4P23_CDAB,4P5T_CDAB,4PWX_ABCD,4RA0_AC,4RS1_AB,4U6H_ABE,4WND_AB,4X4M_ABE,4Y61_AB,4YEB_AB,4YFD_AB,4YH7_AB,4ZS6_HLA,5C6T_HLA,5CXB_AB,5CYK_AB,5E6P_AB,5E9D_ABCDE,5F4E_AB,5K39_AB,5M2O_AB,5TAR_AB,5UFE_AB,5UFQ_AC,5XCO_AB	
1	1A22_AB,1A4Y_AB,1ACB_EI,1AHW_ABC,1AK4_AD,1AO7_ABCDE,1B2S_AD,1B2U_AD,1B3S_AD,1B41_AB,1BD2_ABCDE,1BJ1_HLVW,1BP3_AB,1BRS_AD,1C1Y_AB,1CBW_FGHI,1CHO_EFGI,1CSE_EI,1CSO_EI,1CT0_EI,1CT2_EI,1CT4_EI,1CZ8_HLVW,1DAN_HLUT,1DQJ_ABC,1DVF_ABCD,1E50_AB,1E96_AB,1EAW_AB,1EFN_AB,1EMV_AB,1F47_AB,1F5R_AI,1FC2_CD,1FCC_AC,1FFW_AB,1FR2_AB,1FSS_AB,1FY8_EI,1GC1_GC,1GCQ_ABC,1GL0_EI,1GL1_AI,1GRN_AB,1GUA_AB,1H9D_AB,1HE8_AB,1IAR_AB,1JCK_AB,1JRH_LHI,1JTD_AB,1JTG_AB,1K8R_AB,1KAC_AB,1KBH_AB,1KNE_AP,1KTZ_AB,1LFD_AB,1LP9_ABCEF,1M9E_AD,1MAH_AF,1MI5_ABCDE,1MLC_ABE,1N8O_ABCE,1N8Z_ABC,1NCA_NLH,1NMB_NLH,1P69_AB,1P6A_AB,1PPF_EI,1QSE_ABCDE,1R0R_EI,1REW_ABC,1S0W_AC,1S1Q_AB,1SBB_AB,1SBN_EI,1SGD_EI,1SGE_EI,1SGN_EI,	1C4Z_ABCD,1KIP_ABC,1KIQ_ABC,1KIR_ABC,1MHP_HLA,1OGA_ABCDE,1VFB_ABC,3SGB_EI

	1SGP_EI,1SGQ_EI,1SGY_EI,1SIB_EI,1SMF_EI,1TM1_EI,1TM3_EI,1TM4_EI,1TM5_EI,1TM7_EI,1TMG_EI,1TO1_EI,1U7F_BAC,1UUZ_AD,1WQJ_IB,1X1W_AD,1X1X_AD,1XD3_AB,1XGP_ABC,1XGQ_ABC,1XGR_ABC,1XGT_ABC,1XGU_ABC,1Y1K_EI,1Y33_EI,1Y34_EI,1Y3B_EI,1Y3C_EI,1Y3D_EI,1Y48_EI,1YCS_AB,1YQV_HLY,1YY9_CDA,1Z7X_WX,2A9K_AB,2AJF_AE,2AK4_ABCDE,2AW2_AB,2B0U_ABC,2B0Z_AB,2B10_AB,2B11_AB,2B12_AB,2B2X_HLA,2B42_AB,2BDN_HLA,2BNQ_ABCDE,2BNR_ABCDE,2BTF_AP,2C0L_AB,2C5D_AC,2DSQ_IG,2DVW_AB,2E7L_EQAD,2FTL_EI,2G2U_AB,2G2W_AB,2GOX_AB,2GYK_AB,2HLE_AB,2HRK_AB,2I26_NL,2J0T_AD,2J12_AB,2J1K_CT,2J8U_ABCEF,2JCC_ABCEF,2JEL_LHP,2KSO_AB,2NU0_EI,2NU1_EI,2NU2_EI,2NU4_EI,2NYY_DCA,2NZ9_DCA,2O3B_AB,2OI9_AQBC,2OOB_AB,2P5E_ABCDE,2PCB_AB,2PCC_AB,2REX_AB,2SGP_EI,2SGQ_EI,2SIC_EI,2UWE_ABCEF,2VIR_ABC,2VIS_ABC,2VLN_AB,2VLO_AB,2VLP_AB,2VLQ_AB,2VLR_ABCDE,2WPT_AB,3AAA_ABC,3B4V_ABC,3BK3_AC,3BN9_BCD,3BP8_AC,3BT1_AU,3BTD_EI,3BTE_EI,3BTF_EI,3BTG_EI,3BTH_EI,3BTM_EI,3BTQ_EI,3BTT_EI,3BTW_EI,3BX1_AC,3C60_CDAB,3D3V_ABCDE,3D5R_AC,3D5S_AC,3EG5_AB,3EQS_AB,3EQY_AC,3F1S_AB,3G6D_LHA,3H9S_ABCDE,3HFM_HLY,3HH2_ABC,3KBH_AE,3KUD_AB,3L5X_AHL,3LB6_AC,3LNZ_AB,3LZF_ABHL,3M62_AB,3M63_AB,3MZG_AB,3MZW_AB,3N06_AB,3N0P_AB,3N4I_AB,3N85_ALH,3NCB_AB,3NCC_AB,3NGB_HLG,3NPS_ABC,3NVN_BA,3NVQ_BA,3PWP_ABCDE,3Q3J_AB,3Q8D_AE,3QDG_ABCDE,3QDJ_ABCDE,3QFJ_ABCDE,3QHY_AB,3QIB_ABPCD,3R9A_ACB,3RF3_AC,3S9D_AB,3SE3_BA,3SE4_BA,3SE4_BC,3S	

	E8_HLG,3SE9_HLG,3SEK_BC,3SF4_AD,3SZK_ABC,3TGK_EI,3U82_AB,3UIG_AP,3UII_AP,3VR6_ABCDEFGH,3W2D_AHL,3WWN_AB,4B0M_ABM,4BFI_AB,4CPA_AI,4CVW_AC,4E6K_ABG,4EKD_AB,4FTV_ABCDE,4FZA_AB,4G0N_AB,4G2V_AB,4GNK_AB,4GXU_ABCDEFMN,4HFK_ABD,4HRN_AD,4HSA_ABC,4I77_HLZ,4J2L_ACD,4JEU_AB,4JFD_ABCDE,4JFE_ABCDE,4JFF_ABCDE,4JGH_ABCD,4JPK_HLA,4K71_ABC,4KRL_AB,4KRO_AB,4KRP_AB,4L0P_AB,4L3E_ABCDE,4LRX_ABCD,4MNQ_ABCDE,4MYW_AB,4N8V_GABC,4NKQ_CAB,4NM8_ABCDEFHL,4NZW_AB,4O27_AB,4OFY_AD,4OZG_ABJGH,4P23_CDAB,4P5T_CDAB,4PWX_ABCD,4RA0_AC,4RS1_AB,4U6H_ABE,4WND_AB,4X4M_ABE,4Y61_AB,4YEB_AB,4YFD_AB,4YH7_AB,4ZS6_HLA,5C6T_HLA,5CXB_AB,5CYK_AB,5E6P_AB,5E9D_ABCDE,5F4E_AB,5K39_AB,5M2O_AB,5TAR_AB,5UFE_AB,5UFQ_AC,5XCO_AB	
2	1A22_AB,1A4Y_AB,1ACB_EI,1AHW_ABC,1AK4_AD,1AO7_ABCDE,1B2S_AD,1B2U_AD,1B3S_AD,1B41_AB,1BD2_ABCDE,1BJ1_HLVW,1BRS_AD,1C1Y_AB,1C4Z_ABCD,1CBW_FGHI,1CSE_EI,1CSO_EI,1CT0_EI,1CT2_EI,1CT4_EI,1CZ8_HLVW,1DAN_HLUT,1DQJ_ABC,1DVF_ABCD,1E50_AB,1E96_AB,1EAW_AB,1EFN_AB,1EMV_AB,1F47_AB,1F5R_AI,1FC2_CD,1FCC_AC,1FFW_AB,1FR2_AB,1FSS_AB,1FY8_EI,1GC1_GC,1GCQ_ABC,1GL0_EI,1GL1_AI,1GRN_AB,1GUA_AB,1H9D_AB,1HE8_AB,1IAR_AB,1JCK_AB,1JTD_AB,1JTG_AB,1K8R_AB,1KAC_AB,1KBH_AB,1KIP_ABC,1KIQ_ABC,1KIR_ABC,1KNE_AP,1KTZ_AB,1LP9_ABCEF,1M9E_AD,1MAH_AF,1MHP_HLA,1MI5_ABCDE,1MLC_ABE,1N8O_ABCE,1N8Z_ABC,1NCA_NLH,1NMB_NLH,1OGA_ABCDE,1P69_AB,1P6A_AB,1PPF	1BP3_AB,1CHO_EFGI,1JRH_LHI,1LFD_AB,2JEL_LHP,2REX_AB,3NGB_HLG

	_EI,1QSE_ABCDE,1R0R_EI,1REW_ABC,1S0W_AC,1S1Q_AB,1SBB_AB,1SBN_EI,1SGD_EI,1SGE_EI,1SGN_EI,1SGP_EI,1SGQ_EI,1SGY_EI,1SIB_EI,1SMF_EI,1TM1_EI,1TM3_EI,1TM4_EI,1TM5_EI,1TM7_EI,1TMG_EI,1TO1_EI,1U7F_BAC,1UUZ_AD,1VFB_ABC,1WQJ_IB,1X1W_AD,1X1X_AD,1XD3_AB,1XGP_ABC,1XGQ_ABC,1XGR_ABC,1XGT_ABC,1XGU_ABC,1Y1K_EI,1Y33_EI,1Y34_EI,1Y3B_EI,1Y3C_EI,1Y3D_EI,1Y48_EI,1YCS_AB,1YQV_HLY,1YY9_CDA,1Z7X_WX,2A9K_AB,2AJF_AE,2AK4_ABCDE,2AW2_AB,2B0U_ABC,2B0Z_AB,2B10_AB,2B11_AB,2B12_AB,2B2X_HLA,2B42_AB,2BDN_HLA,2BNQ_ABCDE,2BNR_ABCDE,2BTF_AP,2C0L_AB,2C5D_AC,2DSQ_IG,2DVW_AB,2E7L_EQAD,2FTL_EI,2G2U_AB,2G2W_AB,2GOX_AB,2GYK_AB,2HLE_AB,2HRK_AB,2I26_NL,2J0T_AD,2J12_AB,2J1K_CT,2J8U_ABCEF,2JCC_ABCEF,2KSO_AB,2NU0_EI,2NU1_EI,2NU2_EI,2NU4_EI,2NYY_DCA,2NZ9_DCA,2O3B_AB,2OI9_AQBC,2OOB_AB,2P5E_ABCDE,2PCB_AB,2PCC_AB,2SGP_EI,2SGQ_EI,2SIC_EI,2UWE_ABCEF,2VIR_ABC,2VIS_ABC,2VLN_AB,2VLO_AB,2VLP_AB,2VLQ_AB,2VLR_ABCDE,2WPT_AB,3AAA_ABC,3B4V_ABC,3BK3_AC,3BN9_BCD,3BP8_AC,3BT1_AU,3BTD_EI,3BTE_EI,3BTF_EI,3BTG_EI,3BTH_EI,3BTM_EI,3BTQ_EI,3BTT_EI,3BTW_EI,3BX1_AC,3C60_CDAB,3D3V_ABCDE,3D5R_AC,3D5S_AC,3EG5_AB,3EQS_AB,3EQY_AC,3F1S_AB,3G6D_LHA,3H9S_ABCDE,3HFM_HLY,3HH2_ABC,3KBH_AE,3KUD_AB,3L5X_AHL,3LB6_AC,3LNZ_AB,3LZF_ABHL,3M62_AB,3M63_AB,3MZG_AB,3MZW_AB,3N06_AB,3N0P_AB,3N4I_AB,3N85_ALH,3NCB_AB,3NCC_AB,3NPS_ABC,3NVN_BA,3NVQ_BA,3PWP_ABCDE,3Q3J_AB,3Q8D_AE,3QDG_ABCDE,3QDJ_ABCDE,3QFJ_ABCDE,3Q	

	HY_AB,3QIB_ABPCD,3R9A_ACB,3RF3_AC,3S9D_AB,3SE3_BA,3SE4_BA,3SE4_BC,3SE8_HLG,3SE9_HLG,3SEK_BC,3SF4_AD,3SGB_EI,3SZK_ABC,3TGK_EI,3U82_AB,3UIG_AP,3UII_AP,3VR6_ABCDEFGH,3W2D_AHL,3WWN_AB,4B0M_ABM,4BFI_AB,4CPA_AI,4CVW_AC,4E6K_ABG,4EKD_AB,4FTV_ABCDE,4FZA_AB,4G0N_AB,4G2V_AB,4GNK_AB,4GXU_ABCDEFMN,4HFK_ABD,4HRN_AD,4HSA_ABC,4I77_HLZ,4J2L_ACD,4JEU_AB,4JFD_ABCDE,4JFE_ABCDE,4JFF_ABCDE,4JGH_ABCD,4JPK_HLA,4K71_ABC,4KRL_AB,4KRO_AB,4KRP_AB,4L0P_AB,4L3E_ABCDE,4LRX_ABCD,4MNQ_ABCDE,4MYW_AB,4N8V_GABC,4NKQ_CAB,4NM8_ABCDEFHL,4NZW_AB,4O27_AB,4OFY_AD,4OZG_ABJGH,4P23_CDAB,4P5T_CDAB,4PWX_ABCD,4RA0_AC,4RS1_AB,4U6H_ABE,4WND_AB,4X4M_ABE,4Y61_AB,4YEB_AB,4YFD_AB,4YH7_AB,4ZS6_HLA,5C6T_HLA,5CXB_AB,5CYK_AB,5E6P_AB,5E9D_ABCDE,5F4E_AB,5K39_AB,5M2O_AB,5TAR_AB,5UFE_AB,5UFQ_AC,5XCO_AB	
3	1A22_AB,1A4Y_AB,1ACB_EI,1AHW_ABC,1AK4_AD,1AO7_ABCDE,1B2S_AD,1B2U_AD,1B3S_AD,1B41_AB,1BD2_ABCDE,1BJ1_HLVW,1BP3_AB,1BRS_AD,1C1Y_AB,1C4Z_ABCD,1CBW_FGHI,1CHO_EFGI,1CSE_EI,1CSO_EI,1CT0_EI,1CT2_EI,1CT4_EI,1CZ8_HLVW,1DAN_HLUT,1DQJ_ABC,1DVF_ABCD,1E50_AB,1E96_AB,1EAW_AB,1EFN_AB,1EMV_AB,1F47_AB,1F5R_AI,1FC2_CD,1FCC_AC,1FFW_AB,1FR2_AB,1FSS_AB,1FY8_EI,1GC1_GC,1GCQ_ABC,1GL0_EI,1GL1_AI,1GRN_AB,1GUA_AB,1H9D_AB,1HE8_AB,1JCK_AB,1JRH_LHI,1JTG_AB,1K8R_AB,1KAC_AB,1KBH_AB,1KIP_ABC,1KIQ_ABC,1KIR_ABC,1KNE_AP,1KTZ_AB,1LFD_AB,1LP9_ABCEF,1M9E_AD,1MAH_AF,1MHP_HLA,1MI5_ABCDE,1	1IAR_AB,1JTD_AB,1R0R_EI,3BN9_BCD,3C60_CDAB,4NKQ_CAB,4RS1_AB

	MLC_ABE,1N8O_ABCE,1N8Z_ABC,1NCA_NLH,1NMB_NLH,1OGA_ABCDE,1P69_AB,1P6A_AB,1PPF_EI,1QSE_ABCDE,1REW_ABC,1S0W_AC,1S1Q_AB,1SBB_AB,1SBN_EI,1SGD_EI,1SGE_EI,1SGN_EI,1SGP_EI,1SGQ_EI,1SGY_EI,1SIB_EI,1SMF_EI,1TM1_EI,1TM3_EI,1TM4_EI,1TM5_EI,1TM7_EI,1TMG_EI,1TO1_EI,1U7F_BAC,1UUZ_AD,1VFB_ABC,1WQJ_IB,1X1W_AD,1X1X_AD,1XD3_AB,1XGP_ABC,1XGQ_ABC,1XGR_ABC,1XGT_ABC,1XGU_ABC,1Y1K_EI,1Y33_EI,1Y34_EI,1Y3B_EI,1Y3C_EI,1Y3D_EI,1Y48_EI,1YCS_AB,1YQV_HLY,1YY9_CDA,1Z7X_WX,2A9K_AB,2AJF_AE,2AK4_ABCDE,2AW2_AB,2B0U_ABC,2B0Z_AB,2B10_AB,2B11_AB,2B12_AB,2B2X_HLA,2B42_AB,2BDN_HLA,2BNQ_ABCDE,2BNR_ABCDE,2BTF_AP,2C0L_AB,2C5D_AC,2DSQ_IG,2DVW_AB,2E7L_EQAD,2FTL_EI,2G2U_AB,2G2W_AB,2GOX_AB,2GYK_AB,2HLE_AB,2HRK_AB,2I26_NL,2J0T_AD,2J12_AB,2J1K_CT,2J8U_ABCEF,2JCC_ABCEF,2JEL_LHP,2KSO_AB,2NU0_EI,2NU1_EI,2NU2_EI,2NU4_EI,2NYY_DCA,2NZ9_DCA,2O3B_AB,2OI9_AQBC,2OOB_AB,2P5E_ABCDE,2PCB_AB,2PCC_AB,2REX_AB,2SGP_EI,2SGQ_EI,2SIC_EI,2UWE_ABCEF,2VIR_ABC,2VIS_ABC,2VLN_AB,2VLO_AB,2VLP_AB,2VLQ_AB,2VLR_ABCDE,2WPT_AB,3AAA_ABC,3B4V_ABC,3BK3_AC,3BP8_AC,3BT1_AU,3BTD_EI,3BTE_EI,3BTF_EI,3BTG_EI,3BTH_EI,3BTM_EI,3BTQ_EI,3BTT_EI,3BTW_EI,3BX1_AC,3D3V_ABCDE,3D5R_AC,3D5S_AC,3EG5_AB,3EQS_AB,3EQY_AC,3F1S_AB,3G6D_LHA,3H9S_ABCDE,3HFM_HLY,3HH2_ABC,3KBH_AE,3KUD_AB,3L5X_AHL,3LB6_AC,3LNZ_AB,3LZF_ABHL,3M62_AB,3M63_AB,3MZG_AB,3MZW_AB,3N06_AB,3N0P_AB,3N4I_AB,3N85_ALH,3NCB_AB,3NCC_AB,3NGB_HLG,3NPS_ABC,3NVN_	

	BA,3NVQ_BA,3PWP_ABCDE,3Q3J_AB,3Q8D_AE,3QDG_ABCDE,3QDJ_ABCDE,3QFJ_ABCDE,3QHY_AB,3QIB_ABPCD,3R9A_ACB,3RF3_AC,3S9D_AB,3SE3_BA,3SE4_BA,3SE4_BC,3SE8_HLG,3SE9_HLG,3SEK_BC,3SF4_AD,3SGB_EI,3SZK_ABC,3TGK_EI,3U82_AB,3UIG_AP,3UII_AP,3VR6_ABCDEFGH,3W2D_AHL,3WWN_AB,4B0M_ABM,4BFI_AB,4CPA_AI,4CVW_AC,4E6K_ABG,4EKD_AB,4FTV_ABCDE,4FZA_AB,4G0N_AB,4G2V_AB,4GNK_AB,4GXU_ABCDEFMN,4HFK_ABD,4HRN_AD,4HSA_ABC,4I77_HLZ,4J2L_ACD,4JEU_AB,4JFD_ABCDE,4JFE_ABCDE,4JFF_ABCDE,4JGH_ABCD,4JPK_HLA,4K71_ABC,4KRL_AB,4KRO_AB,4KRP_AB,4L0P_AB,4L3E_ABCDE,4LRX_ABCD,4MNQ_ABCDE,4MYW_AB,4N8V_GABC,4NM8_ABCDEFHL,4NZW_AB,4O27_AB,4OFY_AD,4OZG_ABJGH,4P23_CDAB,4P5T_CDAB,4PWX_ABCD,4RA0_AC,4U6H_ABE,4WND_AB,4X4M_ABE,4Y61_AB,4YEB_AB,4YFD_AB,4YH7_AB,4ZS6_HLA,5C6T_HLA,5CXB_AB,5CYK_AB,5E6P_AB,5E9D_ABCDE,5F4E_AB,5K39_AB,5M2O_AB,5TAR_AB,5UFE_AB,5UFQ_AC,5XCO_AB	
4	1A22_AB,1A4Y_AB,1ACB_EI,1AHW_ABC,1AK4_AD,1AO7_ABCDE,1B2S_AD,1B2U_AD,1B3S_AD,1B41_AB,1BD2_ABCDE,1BJ1_HLVW,1BP3_AB,1C1Y_AB,1C4Z_ABCD,1CHO_EFGI,1CSE_EI,1CSO_EI,1CT0_EI,1CT2_EI,1CT4_EI,1CZ8_HLVW,1DAN_HLUT,1DQJ_ABC,1DVF_ABCD,1E50_AB,1E96_AB,1EAW_AB,1EFN_AB,1EMV_AB,1F47_AB,1F5R_AI,1FC2_CD,1FCC_AC,1FFW_AB,1FR2_AB,1FSS_AB,1FY8_EI,1GC1_GC,1GCQ_ABC,1GL0_EI,1GL1_AI,1GRN_AB,1GUA_AB,1H9D_AB,1HE8_AB,1IAR_AB,1JCK_AB,1JRH_LHI,1JTD_AB,1JTG_AB,1K8R_AB,1KAC_AB,1KBH_AB,1KIP_ABC,1KIQ_ABC,1KIR_ABC,1KNE_AP,1KTZ_AB,1LF	1BRS_AD,1CBW_FGHI,1PPF_EI,2AK4_ABCDE,2FTL_EI,2G2U_AB,2NYY_DCA,2WPT_AB,3SE8_HLG

	D_AB,1LP9_ABCEF,1M9E_AD,1MAH_AF,1MHP_HLA,1MI5_ABCDE,1MLC_ABE,1N8O_ABCE,1N8Z_ABC,1NCA_NLH,1NMB_NLH,1OGA_ABCDE,1P69_AB,1P6A_AB,1QSE_ABCDE,1R0R_EI,1REW_ABC,1S0W_AC,1S1Q_AB,1SBB_AB,1SBN_EI,1SGD_EI,1SGE_EI,1SGN_EI,1SGP_EI,1SGQ_EI,1SGY_EI,1SIB_EI,1SMF_EI,1TM1_EI,1TM3_EI,1TM4_EI,1TM5_EI,1TM7_EI,1TMG_EI,1TO1_EI,1U7F_BAC,1UUZ_AD,1VFB_ABC,1WQJ_IB,1X1W_AD,1X1X_AD,1XD3_AB,1XGP_ABC,1XGQ_ABC,1XGR_ABC,1XGT_ABC,1XGU_ABC,1Y1K_EI,1Y33_EI,1Y34_EI,1Y3B_EI,1Y3C_EI,1Y3D_EI,1Y48_EI,1YCS_AB,1YQV_HLY,1YY9_CDA,1Z7X_WX,2A9K_AB,2AJF_AE,2AW2_AB,2B0U_ABC,2B0Z_AB,2B10_AB,2B11_AB,2B12_AB,2B2X_HLA,2B42_AB,2BDN_HLA,2BNQ_ABCDE,2BNR_ABCDE,2BTF_AP,2C0L_AB,2C5D_AC,2DSQ_IG,2DVW_AB,2E7L_EQAD,2G2W_AB,2GOX_AB,2GYK_AB,2HLE_AB,2HRK_AB,2I26_NL,2J0T_AD,2J12_AB,2J1K_CT,2J8U_ABCEF,2JCC_ABCEF,2JEL_LHP,2KSO_AB,2NU0_EI,2NU1_EI,2NU2_EI,2NU4_EI,2NZ9_DCA,2O3B_AB,2OI9_AQBC,2OOB_AB,2P5E_ABCDE,2PCB_AB,2PCC_AB,2REX_AB,2SGP_EI,2SGQ_EI,2SIC_EI,2UWE_ABCEF,2VIR_ABC,2VIS_ABC,2VLN_AB,2VLO_AB,2VLP_AB,2VLQ_AB,2VLR_ABCDE,3AAA_ABC,3B4V_ABC,3BK3_AC,3BN9_BCD,3BP8_AC,3BT1_AU,3BTD_EI,3BTE_EI,3BTF_EI,3BTG_EI,3BTH_EI,3BTM_EI,3BTQ_EI,3BTT_EI,3BTW_EI,3BX1_AC,3C60_CDAB,3D3V_ABCDE,3D5R_AC,3D5S_AC,3EG5_AB,3EQS_AB,3EQY_AC,3F1S_AB,3G6D_LHA,3H9S_ABCDE,3HFM_HLY,3HH2_ABC,3KBH_AE,3KUD_AB,3L5X_AHL,3LB6_AC,3LNZ_AB,3LZF_ABHL,3M62_AB,3M63_AB,3MZG_AB,3MZW_AB,3N06_AB,3N0P_AB,3N4I_AB,3N85_ALH,3NCB_AB,3N	

	CC_AB,3NGB_HLG,3NPS_ABC,3NVN_BA,3NVQ_BA,3PWP_ABCDE,3Q3J_AB,3Q8D_AE,3QDG_ABCDE,3QDJ_ABCDE,3QFJ_ABCDE,3QHY_AB,3QIB_ABPCD,3R9A_ACB,3RF3_AC,3S9D_AB,3SE3_BA,3SE4_BA,3SE4_BC,3SE9_HLG,3SEK_BC,3SF4_AD,3SGB_EI,3SZK_ABC,3TGK_EI,3U82_AB,3UIG_AP,3UII_AP,3VR6_ABCDEFGH,3W2D_AHL,3WWN_AB,4B0M_ABM,4BFI_AB,4CPA_AI,4CVW_AC,4E6K_ABG,4EKD_AB,4FTV_ABCDE,4FZA_AB,4G0N_AB,4G2V_AB,4GNK_AB,4GXU_ABCDEFMN,4HFK_ABD,4HRN_AD,4HSA_ABC,4I77_HLZ,4J2L_ACD,4JEU_AB,4JFD_ABCDE,4JFE_ABCDE,4JFF_ABCDE,4JGH_ABCD,4JPK_HLA,4K71_ABC,4KRL_AB,4KRO_AB,4KRP_AB,4L0P_AB,4L3E_ABCDE,4LRX_ABCD,4MNQ_ABCDE,4MYW_AB,4N8V_GABC,4NKQ_CAB,4NM8_ABCDEFHL,4NZW_AB,4O27_AB,4OFY_AD,4OZG_ABJGH,4P23_CDAB,4P5T_CDAB,4PWX_ABCD,4RA0_AC,4RS1_AB,4U6H_ABE,4WND_AB,4X4M_ABE,4Y61_AB,4YEB_AB,4YFD_AB,4YH7_AB,4ZS6_HLA,5C6T_HLA,5CXB_AB,5CYK_AB,5E6P_AB,5E9D_ABCDE,5F4E_AB,5K39_AB,5M2O_AB,5TAR_AB,5UFE_AB,5UFQ_AC,5XCO_AB	
5	1A4Y_AB,1ACB_EI,1AHW_ABC,1AK4_AD,1AO7_ABCDE,1B2S_AD,1B2U_AD,1B3S_AD,1B41_AB,1BD2_ABCDE,1BJ1_HLVW,1BP3_AB,1BRS_AD,1C1Y_AB,1C4Z_ABCD,1CBW_FGHI,1CHO_EFGI,1CSE_EI,1CSO_EI,1CT0_EI,1CT2_EI,1CT4_EI,1CZ8_HLVW,1DAN_HLUT,1DQJ_ABC,1E50_AB,1E96_AB,1EFN_AB,1EMV_AB,1F47_AB,1F5R_AI,1FC2_CD,1FCC_AC,1FFW_AB,1FR2_AB,1FSS_AB,1FY8_EI,1GC1_GC,1GCQ_ABC,1GL0_EI,1GL1_AI,1GRN_AB,1GUA_AB,1H9D_AB,1HE8_AB,1IAR_AB,1JCK_AB,1JRH_LHI,1JTD_AB,1JTG_AB,1K8R_AB,1KAC_AB	1A22_AB,1DVF_ABCD,1EAW_AB,1KTZ_AB,2BNR_ABCDE,3NPS_ABC,3QHY_AB,3QIB_ABPCD,3SE9_HLG,3U82_AB,4P23_CDAB,4P5T_CDAB,4PWX_ABCD

	,1KBH_AB,1KIP_ABC,1KIQ_ABC,1KIR_ABC,1KNE_AP,1LFD_AB,1LP9_ABCEF,1M9E_AD,1MAH_AF,1MHP_HLA,1MI5_ABCDE,1MLC_ABE,1N8O_ABCE,1N8Z_ABC,1NCA_NLH,1NMB_NLH,1OGA_ABCDE,1P69_AB,1P6A_AB,1PPF_EI,1QSE_ABCDE,1R0R_EI,1REW_ABC,1S0W_AC,1S1Q_AB,1SBB_AB,1SBN_EI,1SGD_EI,1SGE_EI,1SGN_EI,1SGP_EI,1SGQ_EI,1SGY_EI,1SIB_EI,1SMF_EI,1TM1_EI,1TM3_EI,1TM4_EI,1TM5_EI,1TM7_EI,1TMG_EI,1TO1_EI,1U7F_BAC,1UUZ_AD,1VFB_ABC,1WQJ_IB,1X1W_AD,1X1X_AD,1XD3_AB,1XGP_ABC,1XGQ_ABC,1XGR_ABC,1XGT_ABC,1XGU_ABC,1Y1K_EI,1Y33_EI,1Y34_EI,1Y3B_EI,1Y3C_EI,1Y3D_EI,1Y48_EI,1YCS_AB,1YQV_HLY,1YY9_CDA,1Z7X_WX,2A9K_AB,2AJF_AE,2AK4_ABCDE,2AW2_AB,2B0U_ABC,2B0Z_AB,2B10_AB,2B11_AB,2B12_AB,2B2X_HLA,2B42_AB,2BDN_HLA,2BNQ_ABCDE,2BTF_AP,2C0L_AB,2C5D_AC,2DSQ_IG,2DVW_AB,2E7L_EQAD,2FTL_EI,2G2U_AB,2G2W_AB,2GOX_AB,2GYK_AB,2HLE_AB,2HRK_AB,2I26_NL,2J0T_AD,2J12_AB,2J1K_CT,2J8U_ABCEF,2JCC_ABCEF,2JEL_LHP,2KSO_AB,2NU0_EI,2NU1_EI,2NU2_EI,2NU4_EI,2NYY_DCA,2NZ9_DCA,2O3B_AB,2OI9_AQBC,2OOB_AB,2P5E_ABCDE,2PCB_AB,2PCC_AB,2REX_AB,2SGP_EI,2SGQ_EI,2SIC_EI,2UWE_ABCEF,2VIR_ABC,2VIS_ABC,2VLN_AB,2VLO_AB,2VLP_AB,2VLQ_AB,2VLR_ABCDE,2WPT_AB,3AAA_ABC,3B4V_ABC,3BK3_AC,3BN9_BCD,3BP8_AC,3BT1_AU,3BTD_EI,3BTE_EI,3BTF_EI,3BTG_EI,3BTH_EI,3BTM_EI,3BTQ_EI,3BTT_EI,3BTW_EI,3BX1_AC,3C60_CDAB,3D3V_ABCDE,3D5R_AC,3D5S_AC,3EG5_AB,3EQS_AB,3EQY_AC,3F1S_AB,3G6D_LHA,3H9S_ABCDE,3HFM_HLY,3HH2_ABC,3KBH_AE,3KUD_AB,3L5X_AHL,3LB6_AC,3LNZ_A	

	B,3LZF_ABHL,3M62_AB,3M63_AB,3MZG_AB,3MZW_AB,3N06_AB,3N0P_AB,3N4I_AB,3N85_ALH,3NCB_AB,3NCC_AB,3NGB_HLG,3NVN_BA,3NVQ_BA,3PWP_ABCDE,3Q3J_AB,3Q8D_AE,3QDG_ABCDE,3QDJ_ABCDE,3QFJ_ABCDE,3R9A_ACB,3RF3_AC,3S9D_AB,3SE3_BA,3SE4_BA,3SE4_BC,3SE8_HLG,3SEK_BC,3SF4_AD,3SGB_EI,3SZK_ABC,3TGK_EI,3UIG_AP,3UII_AP,3VR6_ABCDEFGH,3W2D_AHL,3WWN_AB,4B0M_ABM,4BFI_AB,4CPA_AI,4CVW_AC,4E6K_ABG,4EKD_AB,4FTV_ABCDE,4FZA_AB,4G0N_AB,4G2V_AB,4GNK_AB,4GXU_ABCDEFMN,4HFK_ABD,4HRN_AD,4HSA_ABC,4I77_HLZ,4J2L_ACD,4JEU_AB,4JFD_ABCDE,4JFE_ABCDE,4JFF_ABCDE,4JGH_ABCD,4JPK_HLA,4K71_ABC,4KRL_AB,4KRO_AB,4KRP_AB,4L0P_AB,4L3E_ABCDE,4LRX_ABCD,4MNQ_ABCDE,4MYW_AB,4N8V_GABC,4NKQ_CAB,4NM8_ABCDEFHL,4NZW_AB,4O27_AB,4OFY_AD,4OZG_ABJGH,4RA0_AC,4RS1_AB,4U6H_ABE,4WND_AB,4X4M_ABE,4Y61_AB,4YEB_AB,4YFD_AB,4YH7_AB,4ZS6_HLA,5C6T_HLA,5CXB_AB,5CYK_AB,5E6P_AB,5E9D_ABCDE,5F4E_AB,5K39_AB,5M2O_AB,5TAR_AB,5UFE_AB,5UFQ_AC,5XCO_AB	
6	1A22_AB,1A4Y_AB,1ACB_EI,1AHW_ABC,1AO7_ABCDE,1B2S_AD,1B2U_AD,1B3S_AD,1BD2_ABCDE,1BJ1_HLVW,1BP3_AB,1BRS_AD,1C1Y_AB,1C4Z_ABCD,1CBW_FGHI,1CHO_EFGI,1CSE_EI,1CSO_EI,1CT0_EI,1CT2_EI,1CT4_EI,1CZ8_HLVW,1DAN_HLUT,1DVF_ABCD,1E96_AB,1EAW_AB,1EFN_AB,1EMV_AB,1F47_AB,1F5R_AI,1FCC_AC,1FR2_AB,1FY8_EI,1GC1_GC,1GCQ_ABC,1GL0_EI,1GL1_AI,1GRN_AB,1GUA_AB,1H9D_AB,1HE8_AB,1IAR_AB,1JCK_AB,1JRH_LHI,1JTD_AB,1JTG_AB,1K8R_AB,1KAC_AB,1KBH_AB,1KIP_ABC,1KIQ_ABC,1K	1AK4_AD,1B41_AB,1DQJ_ABC,1E50_AB,1FC2_CD,1FFW_AB,1FSS_AB,1TM1_EI,1XD3_AB,1XGP_ABC,1XGQ_ABC,1XGR_ABC,1XGT_ABC,1XGU_ABC,1YQV_HLY,1YY9_CDA,1Z7X_WX,2J0T_AD,2NZ9_DCA,3HFM_HLY,3M62_AB,3QDG_ABCDE,3QDJ_ABCDE,3SE3_BA,5C6T_HLA,5M2O_AB

	IR_ABC,1KNE_AP,1KTZ_AB,1LFD_AB,1LP9_ABCEF,1M9E_AD,1MAH_AF,1MHP_HLA,1MI5_ABCDE,1MLC_ABE,1N8O_ABCE,1N8Z_ABC,1NCA_NLH,1NMB_NLH,1OGA_ABCDE,1P69_AB,1P6A_AB,1PPF_EI,1QSE_ABCDE,1R0R_EI,1REW_ABC,1S0W_AC,1S1Q_AB,1SBB_AB,1SBN_EI,1SGD_EI,1SGE_EI,1SGN_EI,1SGP_EI,1SGQ_EI,1SGY_EI,1SIB_EI,1SMF_EI,1TM3_EI,1TM4_EI,1TM5_EI,1TM7_EI,1TMG_EI,1TO1_EI,1U7F_BAC,1UUZ_AD,1VFB_ABC,1WQJ_IB,1X1W_AD,1X1X_AD,1Y1K_EI,1Y33_EI,1Y34_EI,1Y3B_EI,1Y3C_EI,1Y3D_EI,1Y48_EI,1YCS_AB,2A9K_AB,2AJF_AE,2AK4_ABCDE,2AW2_AB,2B0U_ABC,2B0Z_AB,2B10_AB,2B11_AB,2B12_AB,2B2X_HLA,2B42_AB,2BDN_HLA,2BNQ_ABCDE,2BNR_ABCDE,2BTF_AP,2C0L_AB,2C5D_AC,2DSQ_IG,2DVW_AB,2E7L_EQAD,2FTL_EI,2G2U_AB,2G2W_AB,2GOX_AB,2GYK_AB,2HLE_AB,2HRK_AB,2I26_NL,2J12_AB,2J1K_CT,2J8U_ABCEF,2JCC_ABCEF,2JEL_LHP,2KSO_AB,2NU0_EI,2NU1_EI,2NU2_EI,2NU4_EI,2NYY_DCA,2O3B_AB,2OI9_AQBC,2OOB_AB,2P5E_ABCDE,2PCB_AB,2PCC_AB,2REX_AB,2SGP_EI,2SGQ_EI,2SIC_EI,2UWE_ABCEF,2VIR_ABC,2VIS_ABC,2VLN_AB,2VLO_AB,2VLP_AB,2VLQ_AB,2VLR_ABCDE,2WPT_AB,3AAA_ABC,3B4V_ABC,3BK3_AC,3BN9_BCD,3BP8_AC,3BT1_AU,3BTD_EI,3BTE_EI,3BTF_EI,3BTG_EI,3BTH_EI,3BTM_EI,3BTQ_EI,3BTT_EI,3BTW_EI,3BX1_AC,3C60_CDAB,3D3V_ABCDE,3D5R_AC,3D5S_AC,3EG5_AB,3EQS_AB,3EQY_AC,3F1S_AB,3G6D_LHA,3H9S_ABCDE,3HH2_ABC,3KBH_AE,3KUD_AB,3L5X_AHL,3LB6_AC,3LNZ_AB,3LZF_ABHL,3M63_AB,3MZG_AB,3MZW_AB,3N06_AB,3N0P_AB,3N4I_AB,3N85_ALH,3NCB_AB,3NCC_AB,3NGB_HLG,3NPS_ABC,3NVN_BA,3NVQ_BA,	

	3PWP_ABCDE,3Q3J_AB,3Q8D_AE,3QFJ_ABCDE,3QHY_AB,3QIB_ABPCD,3R9A_ACB,3RF3_AC,3S9D_AB,3SE4_BA,3SE4_BC,3SE8_HLG,3SE9_HLG,3SEK_BC,3SF4_AD,3SGB_EI,3SZK_ABC,3TGK_EI,3U82_AB,3UIG_AP,3UII_AP,3VR6_ABCDEFGH,3W2D_AHL,3WWN_AB,4B0M_ABM,4BFI_AB,4CPA_AI,4CVW_AC,4E6K_ABG,4EKD_AB,4FTV_ABCDE,4FZA_AB,4G0N_AB,4G2V_AB,4GNK_AB,4GXU_ABCDEFMN,4HFK_ABD,4HRN_AD,4HSA_ABC,4I77_HLZ,4J2L_ACD,4JEU_AB,4JFD_ABCDE,4JFE_ABCDE,4JFF_ABCDE,4JGH_ABCD,4JPK_HLA,4K71_ABC,4KRL_AB,4KRO_AB,4KRP_AB,4L0P_AB,4L3E_ABCDE,4LRX_ABCD,4MNQ_ABCDE,4MYW_AB,4N8V_GABC,4NKQ_CAB,4NM8_ABCDEFHL,4NZW_AB,4O27_AB,4OFY_AD,4OZG_ABJGH,4P23_CDAB,4P5T_CDAB,4PWX_ABCD,4RA0_AC,4RS1_AB,4U6H_ABE,4WND_AB,4X4M_ABE,4Y61_AB,4YEB_AB,4YFD_AB,4YH7_AB,4ZS6_HLA,5CXB_AB,5CYK_AB,5E6P_AB,5E9D_ABCDE,5F4E_AB,5K39_AB,5TAR_AB,5UFE_AB,5UFQ_AC,5XCO_AB	
7	1A22_AB,1A4Y_AB,1ACB_EI,1AK4_AD,1AO7_ABCDE,1B2S_AD,1B2U_AD,1B3S_AD,1B41_AB,1BP3_AB,1BRS_AD,1C1Y_AB,1C4Z_ABCD,1CBW_FGHI,1CHO_EFGI,1CSE_EI,1CSO_EI,1CT0_EI,1CT2_EI,1CT4_EI,1CZ8_HLVW,1DQJ_ABC,1DVF_ABCD,1E50_AB,1E96_AB,1EAW_AB,1EFN_AB,1EMV_AB,1F5R_AI,1FC2_CD,1FFW_AB,1FR2_AB,1FSS_AB,1FY8_EI,1GC1_GC,1GCQ_ABC,1GL0_EI,1GL1_AI,1GRN_AB,1GUA_AB,1H9D_AB,1HE8_AB,1IAR_AB,1JRH_LHI,1JTD_AB,1JTG_AB,1K8R_AB,1KAC_AB,1KBH_AB,1KIP_ABC,1KIQ_ABC,1KIR_ABC,1KNE_AP,1KTZ_AB,1LFD_AB,1LP9_ABCEF,1M9E_AD,1MHP_HLA,1MI5_ABCDE,1N8O_ABCE,1NCA_NLH,1OGA_AB	1AHW_ABC,1BD2_ABCDE,1BJ1_HLVW,1DAN_HLUT,1F47_AB,1FCC_AC,1JCK_AB,1MAH_AF,1MLC_ABE,1N8Z_ABC,1NMB_NLH,2BDN_HLA,2SIC_EI,3AAA_ABC,3BK3_AC,3EQS_AB,3EQY_AC,3M63_AB,3MZG_AB,3N85_ALH,3Q8D_AE,4B0M_ABM,4BFI_AB,4CPA_AI,4CVW_AC,4HFK_ABD,4I77_HLZ,4JPK_HLA,4NM8_ABCDEFHL,4OFY_AD,4OZG_ABJGH,5E9D_ABCDE,5F4E_AB,5TAR_AB,5XCO_AB

	CDE,1P69_AB,1P6A_AB,1PPF_EI,1QSE_ABCDE,1R0R_EI,1REW_ABC,1S0W_AC,1S1Q_AB,1SBB_AB,1SBN_EI,1SGD_EI,1SGE_EI,1SGN_EI,1SGP_EI,1SGQ_EI,1SGY_EI,1SIB_EI,1SMF_EI,1TM1_EI,1TM3_EI,1TM4_EI,1TM5_EI,1TM7_EI,1TMG_EI,1TO1_EI,1U7F_BAC,1UUZ_AD,1VFB_ABC,1WQJ_IB,1X1W_AD,1X1X_AD,1XD3_AB,1XGP_ABC,1XGQ_ABC,1XGR_ABC,1XGT_ABC,1XGU_ABC,1Y1K_EI,1Y33_EI,1Y34_EI,1Y3B_EI,1Y3C_EI,1Y3D_EI,1Y48_EI,1YCS_AB,1YQV_HLY,1YY9_CDA,1Z7X_WX,2A9K_AB,2AJF_AE,2AK4_ABCDE,2AW2_AB,2B0U_ABC,2B0Z_AB,2B10_AB,2B11_AB,2B12_AB,2B2X_HLA,2B42_AB,2BNQ_ABCDE,2BNR_ABCDE,2BTF_AP,2C0L_AB,2C5D_AC,2DSQ_IG,2DVW_AB,2E7L_EQAD,2FTL_EI,2G2U_AB,2G2W_AB,2GOX_AB,2GYK_AB,2HLE_AB,2HRK_AB,2I26_NL,2J0T_AD,2J12_AB,2J1K_CT,2J8U_ABCEF,2JCC_ABCEF,2JEL_LHP,2KSO_AB,2NU0_EI,2NU1_EI,2NU2_EI,2NU4_EI,2NYY_DCA,2NZ9_DCA,2O3B_AB,2OI9_AQBC,2OOB_AB,2P5E_ABCDE,2PCB_AB,2PCC_AB,2REX_AB,2SGP_EI,2SGQ_EI,2UWE_ABCEF,2VIR_ABC,2VIS_ABC,2VLN_AB,2VLO_AB,2VLP_AB,2VLQ_AB,2VLR_ABCDE,2WPT_AB,3B4V_ABC,3BN9_BCD,3BP8_AC,3BT1_AU,3BTD_EI,3BTE_EI,3BTF_EI,3BTG_EI,3BTH_EI,3BTM_EI,3BTQ_EI,3BTT_EI,3BTW_EI,3BX1_AC,3C60_CDAB,3D3V_ABCDE,3D5R_AC,3D5S_AC,3EG5_AB,3F1S_AB,3G6D_LHA,3H9S_ABCDE,3HFM_HLY,3HH2_ABC,3KBH_AE,3KUD_AB,3L5X_AHL,3LB6_AC,3LNZ_AB,3LZF_ABHL,3M62_AB,3MZW_AB,3N06_AB,3N0P_AB,3N4I_AB,3NCB_AB,3NCC_AB,3NGB_HLG,3NPS_ABC,3NVN_BA,3NVQ_BA,3PWP_ABCDE,3Q3J_AB,3QDG_ABCDE,3QDJ_ABCDE,3QFJ_ABCDE,3QHY_AB,3QIB_ABPCD,3R9A_ACB,3RF3_AC,3S9	

8	D_AB,3SE3_BA,3SE4_BA,3SE4_BC,3SE8_HLG,3SE9_HLG,3SEK_BC,3SF4_AD,3SGB_EI,3SZK_ABC,3TGK_EI,3U82_AB,3UIG_AP,3UII_AP,3VR6_ABCDEFGH,3W2D_AHL,3WWN_AB,4E6K_ABG,4EKD_AB,4FTV_ABCDE,4FZA_AB,4G0N_AB,4G2V_AB,4GNK_AB,4GXU_ABCDEFMN,4HRN_AD,4HSA_ABC,4J2L_ACD,4JEU_AB,4JFD_ABCDE,4JFE_ABCDE,4JFF_ABCDE,4JGH_ABCD,4K71_ABC,4KRL_AB,4KRO_AB,4KRP_AB,4L0P_AB,4L3E_ABCDE,4LRX_ABCD,4MNQ_ABCDE,4MYW_AB,4N8V_GABC,4NKQ_CAB,4NZW_AB,4O27_AB,4P23_CDAB,4P5T_CDAB,4PWX_ABCD,4RA0_AC,4RS1_AB,4U6H_ABE,4WND_AB,4X4M_ABE,4Y61_AB,4YEB_AB,4YFD_AB,4YH7_AB,4ZS6_HLA,5C6T_HLA,5CXB_AB,5CYK_AB,5E6P_AB,5K39_AB,5M2O_AB,5UFE_AB,5UFQ_AC	
8	1A22_AB,1A4Y_AB,1AHW_ABC,1AK4_AD,1B3S_AD,1B41_AB,1BD2_ABCDE,1BJ1_HLVW,1BP3_AB,1BRS_AD,1C1Y_AB,1C4Z_ABCD,1CBW_FGHI,1CHO_EFGI,1CSO_EI,1CT0_EI,1CT2_EI,1CT4_EI,1CZ8_HLVW,1DAN_HLUT,1DQJ_ABC,1DVF_ABCD,1E50_AB,1EAW_AB,1EFN_AB,1EMV_AB,1F47_AB,1F5R_AI,1FC2_CD,1FCC_AC,1FFW_AB,1FR2_AB,1FSS_AB,1GC1_GC,1GL0_EI,1GL1_AI,1GRN_AB,1GUA_AB,1HE8_AB,1IAR_AB,1JCK_AB,1JRH_LHI,1JTD_AB,1JTG_AB,1K8R_AB,1KAC_AB,1KIP_ABC,1KIQ_ABC,1KIR_ABC,1KNE_AP,1KTZ_AB,1LFD_AB,1M9E_AD,1MAH_AF,1MHP_HLA,1MI5_ABCDE,1MLC_ABE,1N8Z_ABC,1NMB_NLH,1OGA_ABCDE,1P69_AB,1P6A_AB,1PPF_EI,1QSE_ABCDE,1R0R_EI,1REW_ABC,1S0W_AC,1SBN_EI,1SGD_EI,1SGE_EI,1SGN_EI,1SGP_EI,1SGQ_EI,1SGY_EI,1SIB_EI,1TM1_EI,1TM3_EI,1TM4_EI,1TM5_EI,1TM7_EI,1TMG_EI,1TO1_EI,1U7F_BAC,1VFB_ABC,1X1W	1ACB_EI,1AO7_ABCDE,1B2S_AD,1B2U_AD,1CSE_EI,1E96_AB,1FY8_EI,1GCQ_ABC,1H9D_AB,1KBH_AB,1LP9_ABCEF,1N8O_ABCE,1NCA_NLH,1S1Q_AB,1SBB_AB,1SMF_EI,1UUZ_AD,1WQJ_IB,2AJF_AE,2AW2_AB,2B0U_ABC,2B2X_HLA,2B42_AB,2BTF_AP,2C0L_AB,2C5D_AC,2DSQ_IG,2DVW_AB,2GOX_AB,2HRK_AB,2J1K_CT,2KSO_AB,2O3B_AB,2OOB_AB,2PCB_AB,2PCC_AB,3B4V_ABC,3D3V_ABCDE,3F1S_AB,3HH2_ABC,3KBH_AE,3LB6_AC,3MZW_AB,3R9A_ACB,3SEK_BC,3SF4_AD,3UIG_AP,3VR6_ABCDEFGH,3W2D_AHL,3WWN_AB,4E6K_ABG,4FTV_ABCDE,4FZA_AB,4GNK_AB,4HRN_AD,4HSA_ABC,4J2L_ACD,4JEU_AB,4JFF_ABCDE,4K71_ABC,4L0P_AB,4L3E_ABCDE,4MYW_AB,4N8V_GABC,4NZW_AB,4O27_AB,4RA0_AC,4X4M_ABE,4YEB_AB,4YFD_AB,4YH7_AB,5CXB_AB,5CYK_AB,5E6P_AB,5K39_AB,5UFE_AB,5UFQ_AC

	_AD,1X1X_AD,1XD3_AB,1XGP_ABC,1XGQ_ABC,1XGR_ABC,1XGT_ABC,1XGU_ABC,1Y1K_EI,1Y33_EI,1Y34_EI,1Y3B_EI,1Y3C_EI,1Y3D_EI,1Y48_EI,1YCS_AB,1YQV_HLY,1YY9_CDA,1Z7X_WX,2A9K_AB,2AK4_ABCDE,2B0Z_AB,2B10_AB,2B11_AB,2B12_AB,2BDN_HLA,2BNQ_ABCDE,2BNR_ABCDE,2E7L_EQAD,2FTL_EI,2G2U_AB,2G2W_AB,2GYK_AB,2HLE_AB,2I26_NL,2J0T_AD,2J12_AB,2J8U_ABCEF,2JCC_ABCEF,2JEL_LHP,2NU0_EI,2NU1_EI,2NU2_EI,2NU4_EI,2NYY_DCA,2NZ9_DCA,2OI9_AQBC,2P5E_ABCDE,2REX_AB,2SGP_EI,2SGQ_EI,2SIC_EI,2UWE_ABCEF,2VIR_ABC,2VIS_ABC,2VLN_AB,2VLO_AB,2VLP_AB,2VLQ_AB,2VLR_ABCDE,2WPT_AB,3AAA_ABC,3BK3_AC,3BN9_BCD,3BP8_AC,3BT1_AU,3BTD_EI,3BTE_EI,3BTF_EI,3BTG_EI,3BTH_EI,3BTM_EI,3BTQ_EI,3BTT_EI,3BTW_EI,3BX1_AC,3C60_CDAB,3D5R_AC,3D5S_AC,3EG5_AB,3EQS_AB,3EQY_AC,3G6D_LHA,3H9S_ABCDE,3HFM_HLY,3KUD_AB,3L5X_AHL,3LNZ_AB,3LZF_ABHL,3M62_AB,3M63_AB,3MZG_AB,3N06_AB,3N0P_AB,3N4I_AB,3N85_ALH,3NCB_AB,3NCC_AB,3NGB_HLG,3NPS_ABC,3NVN_BA,3NVQ_BA,3PWP_ABCDE,3Q3J_AB,3Q8D_AE,3QDG_ABCDE,3QDJ_ABCDE,3QFJ_ABCDE,3QHY_AB,3QIB_ABPCD,3RF3_AC,3S9D_AB,3SE3_BA,3SE4_BA,3SE4_BC,3SE8_HLG,3SE9_HLG,3SGB_EI,3SZK_ABC,3TGK_EI,3U82_AB,3UII_AP,4B0M_ABM,4BFI_AB,4CPA_AI,4CVW_AC,4EKD_AB,4G0N_AB,4G2V_AB,4GXU_ABCDEFMN,4HFK_ABD,4I77_HLZ,4JFD_ABCDE,4JFE_ABCDE,4JGH_ABCD,4JPK_HLA,4KRL_AB,4KRO_AB,4KRP_AB,4LRX_ABCD,4MNQ_ABCDE,4NKQ_CAB,4NM8_ABCDEFHL,4OFY_AD,4OZG_ABJGH,4P23_CDAB,4P5T_CDAB,4PWX_ABCD,4RS1_AB,4U6H_ABE,4WND_AB,4	

	Y61_AB,4ZS6_HLA,5C6T_HLA,5E9D_ABCDE,5F4E_AB,5M2O_AB,5TAR_AB,5XCO_AB	
9	1A22_AB,1ACB_EI,1AHW_ABC,1AK4_AD,1AO7_ABCDE,1B2S_AD,1B2U_AD,1B41_AB,1BD2_ABCDE,1BJ1_HLVW,1BP3_AB,1BRS_AD,1C4Z_ABCD,1CBW_FGHI,1CHO_EFGI,1CSE_EI,1DAN_HLUT,1DQJ_ABC,1DVF_ABCD,1E50_AB,1E96_AB,1EAW_AB,1F47_AB,1FC2_CD,1FCC_AC,1FFW_AB,1FSS_AB,1FY8_EI,1GCQ_ABC,1H9D_AB,1IAR_AB,1JCK_AB,1JRH_LHI,1JTD_AB,1JTG_AB,1KBH_AB,1KIP_ABC,1KIQ_ABC,1KIR_ABC,1KTZ_AB,1LFD_AB,1LP9_ABCEF,1MAH_AF,1MHP_HLA,1MLC_ABE,1N8O_ABCE,1N8Z_ABC,1NCA_NLH,1NMB_NLH,1OGA_ABCDE,1PPF_EI,1R0R_EI,1S1Q_AB,1SBB_AB,1SMF_EI,1TM1_EI,1UUZ_AD,1VFB_ABC,1WQJ_IB,1XD3_AB,1XGP_ABC,1XGQ_ABC,1XGR_ABC,1XGT_ABC,1XGU_ABC,1YQV_HLY,1YY9_CDA,1Z7X_WX,2AJF_AE,2AK4_ABCDE,2AW2_AB,2B0U_ABC,2B2X_HLA,2B42_AB,2BDN_HLA,2BNR_ABCDE,2BTF_AP,2C0L_AB,2C5D_AC,2DSQ_IG,2DVW_AB,2FTL_EI,2G2U_AB,2GOX_AB,2HRK_AB,2J0T_AD,2J1K_CT,2JEL_LHP,2KSO_AB,2NYY_DCA,2NZ9_DCA,2O3B_AB,2OOB_AB,2PCB_AB,2PCC_AB,2REX_AB,2SIC_EI,2WPT_AB,3AAA_ABC,3B4V_ABC,3BK3_AC,3BN9_BCD,3BT1_AU,3C60_CDAB,3D3V_ABCDE,3EQS_AB,3EQY_AC,3F1S_AB,3HFM_HLY,3HH2_ABC,3KBH_AE,3LB6_AC,3M62_AB,3M63_AB,3MZG_AB,3MZW_AB,3N85_ALH,3NGB_HLG,3NPS_ABC,3Q8D_AE,3QDG_ABCDE,3QDJ_ABCDE,3QHY_AB,3QIB_ABPCD,3R9A_ACB,3S9D_AB,3SE3_BA,3SE8_HLG,3SE9_HLG,3SEK_BC,3SF4_AD,3SGB_EI,3U82_AB,3UIG_AP,3VR6_ABCDEFGH,3W2D_AHL,3WWN_AB,4B0M_ABM,4BFI_AB,4CPA_AI,4CVW_	1A4Y_AB,1B3S_AD,1C1Y_AB,1CSO_EI,1CT0_EI,1CT2_EI,1CT4_EI,1CZ8_HLVW,1EFN_AB,1EMV_AB,1F5R_AI,1FR2_AB,1GC1_GC,1GL0_EI,1GL1_AI,1GRN_AB,1GUA_AB,1HE8_AB,1K8R_AB,1KAC_AB,1KNE_AP,1M9E_AD,1MI5_ABCDE,1P69_AB,1P6A_AB,1QSE_ABCDE,1REW_ABC,1S0W_AC,1SBN_EI,1SGD_EI,1SGE_EI,1SGN_EI,1SGP_EI,1SGQ_EI,1SGY_EI,1SIB_EI,1TM3_EI,1TM4_EI,1TM5_EI,1TM7_EI,1TMG_EI,1TO1_EI,1U7F_BAC,1X1W_AD,1X1X_AD,1Y1K_EI,1Y33_EI,1Y34_EI,1Y3B_EI,1Y3C_EI,1Y3D_EI,1Y48_EI,1YCS_AB,2A9K_AB,2B0Z_AB,2B10_AB,2B11_AB,2B12_AB,2BNQ_ABCDE,2E7L_EQAD,2G2W_AB,2GYK_AB,2HLE_AB,2I26_NL,2J12_AB,2J8U_ABCEF,2JCC_ABCEF,2NU0_EI,2NU1_EI,2NU2_EI,2NU4_EI,2OI9_AQBC,2P5E_ABCDE,2SGP_EI,2SGQ_EI,2UWE_ABCEF,2VIR_ABC,2VIS_ABC,2VLN_AB,2VLO_AB,2VLP_AB,2VLQ_AB,2VLR_ABCDE,3BP8_AC,3BTD_EI,3BTE_EI,3BTF_EI,3BTG_EI,3BTH_EI,3BTM_EI,3BTQ_EI,3BTT_EI,3BTW_EI,3BX1_AC,3D5R_AC,3D5S_AC,3EG5_AB,3G6D_LHA,3H9S_ABCDE,3KUD_AB,3L5X_AHL,3LNZ_AB,3LZF_ABHL,3N06_AB,3N0P_AB,3N4I_AB,3NCB_AB,3NCC_AB,3NVN_BA,3NVQ_BA,3PWP_ABCDE,3Q3J_AB,3QFJ_ABCDE,3RF3_AC,3SE4_BA,3SE4_BC,3SZK_ABC,3TGK_EI,3UII_AP,4EKD_AB,4G0N_AB,4G2V_AB,4GXU_ABCDEFMN,4JFD_ABCDE,4JFE_ABCDE,4JGH_ABCD,4KRL_AB,4KRO_AB,4KRP_AB,4LRX_ABCD,4MNQ_ABCDE,4U6H_ABE,4WND_AB,4Y61_AB,4ZS6_HLA

	AC,4E6K_ABG,4FTV_ABCDE,4FZA_AB,4GNK_AB,4HFK_ABD,4HRN_AD,4HSA_ABC,4I77_HLZ,4J2L_ACD,4JEU_AB,4JFF_ABCDE,4JPK_HLA,4K71_ABC,4L0P_AB,4L3E_ABCDE,4MYW_AB,4N8V_GABC,4NKQ_CAB,4NM8_ABCDEFHL,4NZW_AB,4O27_AB,4OFY_AD,4OZG_ABJGH,4P23_CDAB,4P5T_CDAB,4PWX_ABCD,4RA0_AC,4RS1_AB,4X4M_ABE,4YEB_AB,4YFD_AB,4YH7_AB,5C6T_HLA,5CXB_AB,5CYK_AB,5E6P_AB,5E9D_ABCDE,5F4E_AB,5K39_AB,5M2O_AB,5TAR_AB,5UFE_AB,5UFQ_AC,5XCO_AB	

Table S6. Effects of different random seeds on baseline models

Seed	Rp	RMSE
2021	0.662	1.840
2022	0.665	1.837
2023	0.657	1.894
2024	0.653	1.913
2025	0.664	1.839

Table S7. Computational environment used for all baseline reproductions

Component	Specification
GPU	NVIDIA GeForce RTX 4090
CPU	13th Gen Intel® Core™ i9-13900K × 32
RAM	128GB
CUDA	12.6
OS	Ubuntu 22.04 LTS

5 Supplementary Figures

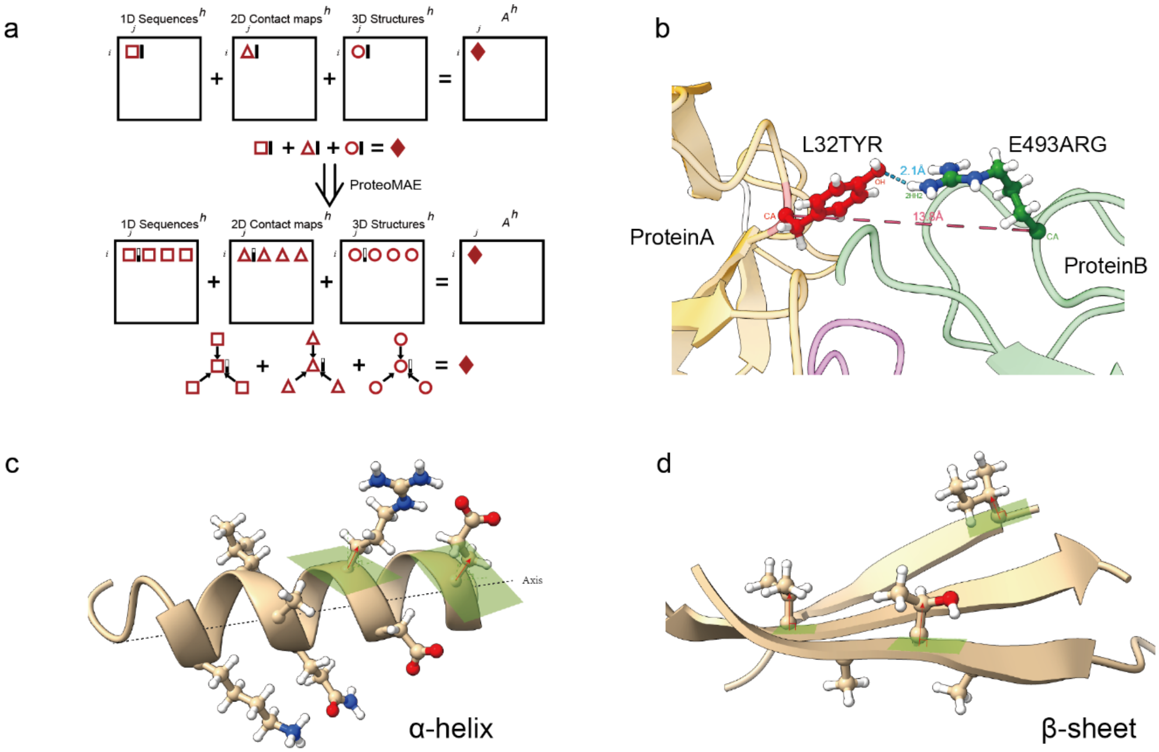

Figure S1. Details of the modules. a, Effect of the ProteoMAE module on modelling. ProteoMAE extends the model from the local receptive field domain to the global receptive field, while utilizing long-range information to selectively emphasize useful features and suppress less useful ones. b, Long-range interactions between residue pairs. In the complex of monoclonal antibody FAB D44.1 and chicken egg white lysozyme, the CA distance between L32TYR and E493ARG is 13.8. A strong interaction is formed as a hydrogen bond forms between the OH group of L32TYR and the 2HH2 group of E493ARG. c, CA and CB point to the center of the helix axis in the alpha helix structure. d, CA and CB in a beta fold are perpendicular to the fold plane.

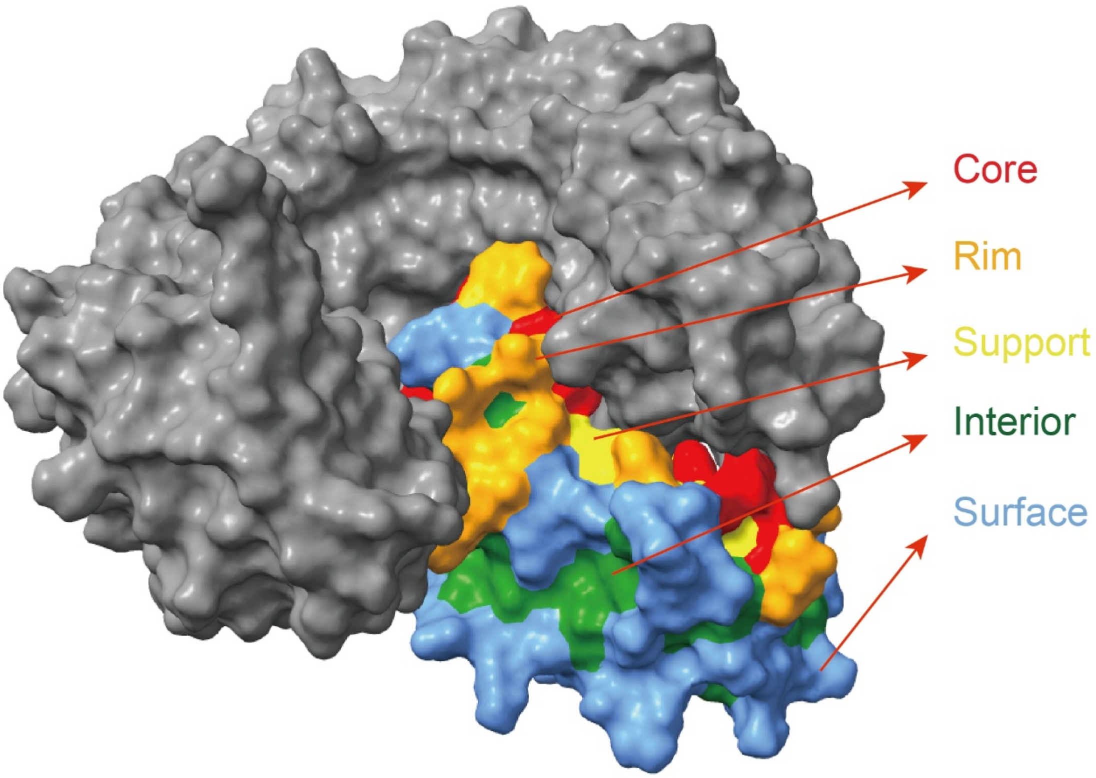

Figure S2. Region classification in protein complexes. Protein B in grey, colored areas are proteins. For protein A, red is core, orange is rim, yellow is support, green is interior, and blue is surface.

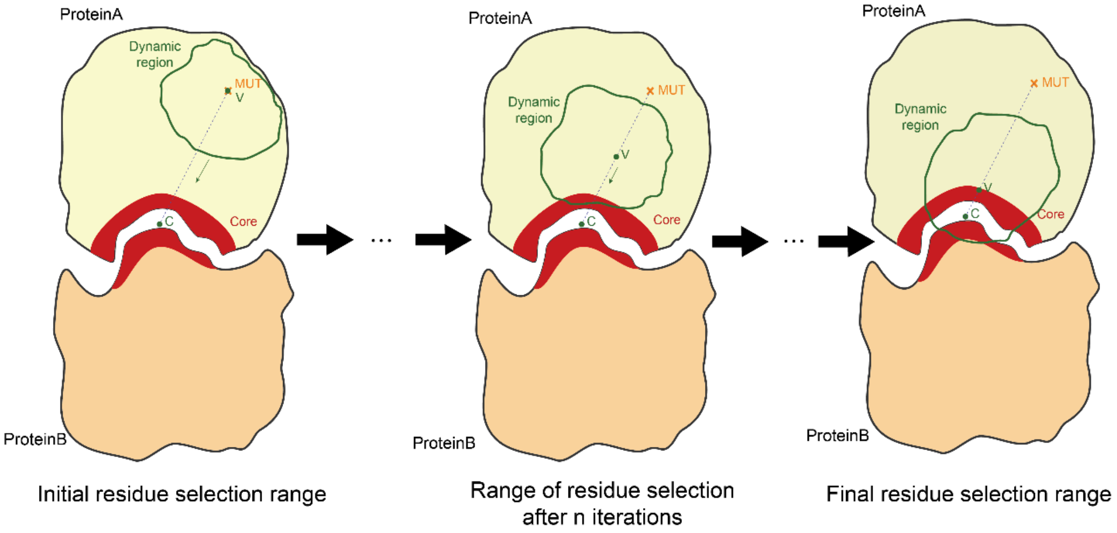

Figure S3. Dynamic residues selection. An iterative process for the optimization of Dynamic residue selection strategies. Taking single-point mutations as an example, the same principle applies to multi-point mutations.

6 SI References

Elnaggar, A., *et al.* Prottrans: Toward understanding the language of life through self-supervised learning. *IEEE transactions on pattern analysis and machine intelligence* 2021;44(10):7112-7127.

Jiang, Y., *et al.* DGCddG: Deep Graph Convolution for Predicting Protein-Protein Binding Affinity Changes Upon Mutations. *IEEE/ACM Transactions on Computational Biology and Bioinformatics* 2023;20(3):2089-2100.

Levy, E.D. A simple definition of structural regions in proteins and its use in analyzing interface evolution. *Journal of molecular biology* 2010;403(4):660-670.

Liu, X., *et al.* Deep geometric representations for modeling effects of mutations on protein-protein binding affinity. *PLOS Computational Biology* 2021;17(8):e1009284.

Miller, S., *et al.* Interior and surface of monomeric proteins. *Journal of molecular biology* 1987;196(3):641-656.

Ying, C., *et al.* Do Transformers Really Perform Bad for Graph Representation? 2021.

Yue, Y., *et al.* MpbPPI: a multi-task pre-training-based equivariant approach for the prediction of the effect of amino acid mutations on protein–protein interactions. *Briefings in Bioinformatics* 2023;24(5).
